# Metal–organic and covalent organic frameworks as single-site catalysts

**DOI:** 10.1039/c7cs00033b

**Published:** 2017-03-24

**Authors:** S. M. J. Rogge, A. Bavykina, J. Hajek, H. Garcia, A. I. Olivos-Suarez, A. Sepúlveda-Escribano, A. Vimont, G. Clet, P. Bazin, F. Kapteijn, M. Daturi, E. V. Ramos-Fernandez, F. X. Llabrés i Xamena, V. Van Speybroeck, J. Gascon

**Affiliations:** a Center for Molecular Modeling , Ghent University , Technologiepark 903 , 9052 Zwijnaarde , Belgium . Email: veronique.vanspeybroeck@ugent.be; b Delft University of Technology , Chemical Engineering Department , Catalysis Engineering , Van der Maasweg 9 , 2629 HZ Delft , The Netherlands . Email: j.gascon@tudelft.nl; c Instituto de Tecnología Química UPV-CSIC , Universitat Politècnica de Valencia , Consejo Superior de Investigaciones Científicas , Avda. de los Naranjos, s/n , 46022 , Valencia , Spain . Email: fllabres@itq.upv.es; d Inorganic Chemistry Department , University Institute of Materials , University of Alicante , Ctra. San Vicente-Alicante s/n , Alicante , Spain . Email: enrique.ramos@ua.es; e Normandie Université , ENSICAEN , UNICAEN , CNRS , Laboratoire Catalyse et Spectrochimie , 14000 Caen , France . Email: Marco.Daturi@ensicaen.fr

## Abstract

Heterogeneous single-site catalysts consist of isolated, well-defined, active sites that are spatially separated in a given solid and, ideally, structurally identical. In this review, the potential of metal–organic frameworks (MOFs) and covalent organic frameworks (COFs) as platforms for the development of heterogeneous single-site catalysts is reviewed thoroughly. In the first part of this article, synthetic strategies and progress in the implementation of such sites in these two classes of materials are discussed. Because these solids are excellent playgrounds to allow a better understanding of catalytic functions, we highlight the most important recent advances in the modelling and spectroscopic characterization of single-site catalysts based on these materials. Finally, we discuss the potential of MOFs as materials in which several single-site catalytic functions can be combined within one framework along with their potential as powerful enzyme-mimicking materials. The review is wrapped up with our personal vision on future research directions.

## Abstract 2

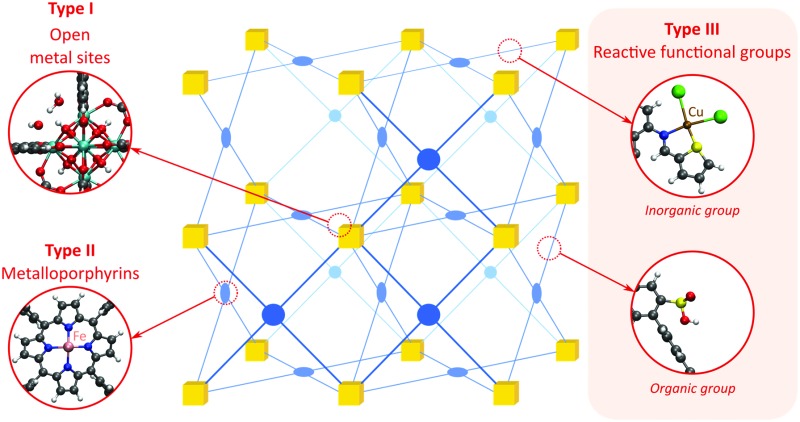
The potential of metal–organic frameworks (MOFs) and covalent organic frameworks (COFs) as platforms for the development of heterogeneous single-site catalysts is reviewed thoroughly.

## A. Introduction

In its classical definition, a catalyst is a substance that increases the rate of a reaction without being consumed considerably. The active site in the catalyst and its interaction with reactant(s), transition state(s), and product(s) define whether the desired reaction will proceed with a higher rate and selectivity at relatively mild conditions compared to the noncatalysed reaction. It is not surprising that the design of such active sites is one of the main targets of catalyst engineering. However, the nature of the active site is not always clear. In the case of homogeneous catalysts and enzymes, they can be easily identified, as discussed in 2005 by Thomas *et al.*:^1^ “*it is easy to comprehend what is meant by the structurally well-defined active site of a metalloenzyme (or any other enzyme) and also by the active site of members of the entire family of homogeneous (i.e. molecular) catalysts in which discrete molecular entities (encompassing the active site) are dispersed in a fluid phase, usually water. No intellectual or practical problems are encountered when these catalysts are referred to as being of the “single-site” variety*”. However, as comprehensively emphasised in Thomas' review,^1^ the description of active sites in the case of heterogeneous catalysts may become more controversial. A typical example is a metal nanoparticle, where the active sites – the metal atoms – may be located at the steps, kinks, terraces, *etc.*, each one of these sites bearing different properties.^2^–^5^ In this sense, one could easily argue that homogeneous catalysis is a much more powerful approach towards the design of better catalytic systems given the rather high level of predictability, design, and engineering of these systems, especially when compared to heterogeneous catalysts. Yet, issues related not only to the obvious challenge of recyclability but also to deactivation and the use of low concentrations of homogeneous catalysts have placed heterogeneous catalysts at the forefront of chemical industry.

The problems presented by both homogeneous and heterogeneous catalysts have triggered intense research over the last few decades in the quest for alternative systems that, ideally, would bridge the gap between these two subdisciplines of catalysis by implementing truly single catalytic sites at the surface of a solid catalyst. The challenge at hand is certainly not trivial: progress in this direction requires the discovery of new materials able to offer sufficient design possibilities as to allow for an exquisite control in the implementation of catalytic functions. This review focuses on, and stresses the advantages of, two relatively new classes of materials that have the potential to become the ideal homo–hetero bridge: metal–organic frameworks (MOFs) and covalent and porous organic frameworks.

MOFs, or more widely speaking, coordination polymers, are known from the late 1950s and early 1960s.^6^–^11^ The field of MOFs has been especially relevant after the seminal works by Robson and co-workers^12^,^13^ Kitagawa *et al.*,^14^,^15^ Yaghi and co-workers,^16^ Lee and Moore,^17^ and Férey and co-worker.^18^ MOFs are crystalline compounds consisting of infinite lattices comprised of inorganic secondary building units (SBUs, metal ions or clusters) and organic linkers, connected by coordination bonds of moderate strength. Distinct from traditional inorganic materials, MOFs can be synthesised from well-defined molecular building blocks and may therefore be understood as molecules arranged in a crystalline lattice.^19^

Porous organic frameworks (POFs) are another class of porous materials that, in contrast to MOFs, are constructed solely from organic building blocks.^20^–^22^ POFs can be classified into two groups depending on the crystallinity of the final solid. Covalent organic frameworks (COFs) are normally synthesised relying on reversible covalent bonds, resulting in highly crystalline materials with mild to low stability. In contrast, amorphous porous organic polymers (POPs) are constructed through irreversible covalent bonds (*e.g.*, C–C bonds). As a result, interpenetrated and non-crystalline structures are normally formed, which however display excellent stability. In both cases, these materials possess high surface area, tuneable pore size, and adjustable skeletons, which brings promise to a wide range of applications. In addition, POFs can be locally decorated with molecular catalysts that may acquire activities and selectivities comparable to their homogeneous analogues. In clear analogy to MOFs, the vast majority of POFs is synthesised in a modular fashion, making straightforward incorporation of functional groups easy and, therefore, opening a promising playground for using POFs as catalysts.

**Fig. 1:**
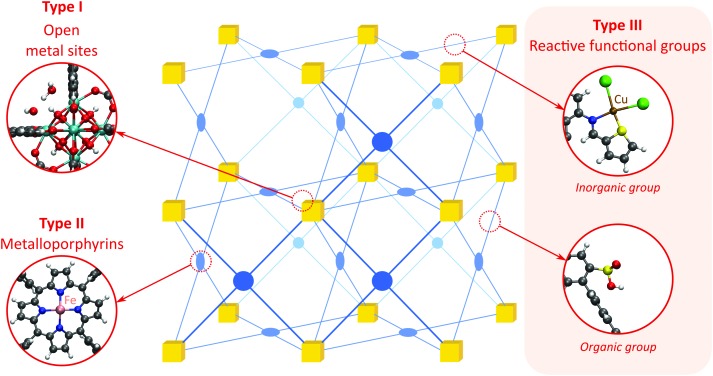
Classification of the different positions in porous framework materials where single-site catalytic reactions can take place. The inorganic nodes are indicated with yellow cubes, whereas the structure-defining ligands are indicated in blue. Possible terminating ligands at the inorganic nodes are not indicated, as they do not contribute to the topology of the material.

As discussed above, heterogeneous single-site catalysts are isolated, well-defined, active sites which are spatially separated in a given solid and, ideally, structurally identical. Conceptually different approaches have been applied to create catalytically active MOFs and POFs and this review is based on a classification into three types of active sites, which are schematically shown in Fig. 1. Within **type I** catalysts, active sites are created by using the structurally embedded metal nodes, which are geometrically undercoordinated – this is clearly only possible in the case of MOFs. These sites are commonly referred to as open metal sites (OMSs). Various strategies may be used to obtain a given degree of undercoordination, which will be further discussed in detail in this review. Within **type II** catalysts, a metal atom embedded in a porphyrin-base ligand acts as active site. Within **type III** catalysts, organic linkers are decorated with covalently anchored functional groups that introduce an active function onto the framework. Apart from the categories introduced here, active heterogeneous catalysts can also be fabricated by embedding nanosized metal clusters within the pores of the MOF or POF. We do not explicitly discuss this type of sites, since other dedicated reviews have already explored this topic.^23^,^24^ Also materials for which catalysis only occurs on the surface of the material or at grain boundaries are hardly touched upon in this review.^25^,^26^

In this article, we present a thorough review on the recent advances in the implementation of single catalytic sites on MOFs and POFs. We first discuss synthetic strategies and progress in the implementation of such sites in Sections B and C for MOFs and POFs, respectively. Because these materials are excellent playgrounds to allow for a better understanding of catalytic functions, we review the most important recent advances in the modelling of single-site catalysts based on these materials in Section D and their spectroscopic characterization in Section E. In Sections F and G, we go one step forward and discuss the potential of MOFs for the combination of several single-site catalytic functions within one framework along with their potential as powerful enzyme-mimicking materials. The review is wrapped up with our personal vision on future research directions. We would like to stress that the literature reviewed here does not cover all catalytic applications of MOFs and POFs. This is mostly because we do believe that the easy implementation of single-site catalytic functions makes both MOFs and POFs unique materials with a large potential for catalysis. For a wider overview on the topic of catalysis by MOFs and POFs, we recommend several recent reviews on the topic.^27^–^32^

## B. Opportunities for heterogeneous single-site catalysis in MOFs

### B.1. Open metal sites

**Fig. 2:**
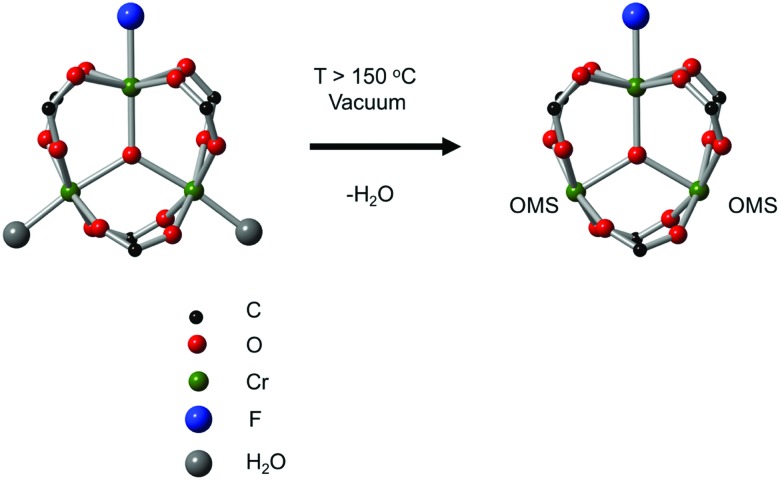
Generation of OMSs in the inorganic clusters of MIL-101(Cr).

Open metal sites (OMSs, also referred to as exposed metal centres, unsaturated metal sites, or coordinatively unsaturated metal sites) in MOFs were first exploited for catalysis by Chen *et al.*^33^ The authors synthesised a MOF in which copper paddlewheels are linked through 1,3,5,7-adamantanetetracarboxylate. Extraction of coordinating guest molecules led to undercoordinated copper sites that can be utilised as Lewis acid sites. After Chen's work, many new structures with open metal sites were prepared, the most famous ones being HKUST-1,^34^ MIL-100(Cr,Fe),^5^,^35^–^40^ MIL-101(Cr,Fe) (see Fig. 2),^41^–^46^ UiO-66,^47^–^53^ and CPO-27(Co,Fe,Mg,Ni),^54^–^58^ all named after the institutes who first synthesised these materials (HKUST = Hong Kong University of Science and Technology, MIL = Matériaux de l'Institut Lavoisier, UiO = Universitetet i Oslo, and CPO = Coordination Polymer of Oslo).

OMSs have been shown to display a certain reactivity and to behave as truly single sites with application not only in catalytic processes but also in other fields like gas adsorption. For example, Yildirim and co-workers demonstrated the importance of OMSs for hydrogen storage,^59^ while others showed that OMSs may play an important role in the separation of hydrocarbons.^60^–^64^

As shown over the last decade, OMSs in MOFs have been used as mild Lewis acids and in the oxidation of organic substrates. The group of Kaskel used HKUST-1 for the liquid phase cyanosilylation of benzaldehyde.^65^ Almost by the same time, Snejko and co-workers prepared a series of indium-based MOFs of medium stability, containing OMSs which were active in the acetalisation of aldehydes.^66^ They prepared four different 2D and 3D compounds. One of them did not contain OMSs, and its catalytic activity was one order of magnitude lower than that of the catalysts with OMSs. It was the first time that MOFs having the same chemical nature – *i.e.* the same metal and type of linker – could be prepared with and without OMSs, and formed a very elegant way to demonstrate the intrinsic catalytic activity of OMSs. Later, De Rosa *et al.* used HKUST-1 for the oxidation of wastewater pollutants, again making use of the OMSs in this structure.^67^ Llabrés i Xamena *et al.* used a palladium-based MOF for alcohol oxidation, Suzuki C–C coupling and olefin hydrogenation.^68^ They found for the first time shape/size selectivities in alkene hydrogenation using MOFs as catalysts. Thus, bulky molecules could not be hydrogenated because they were too large to enter the pores and reach the active sites. Some years later, Klemm and co-workers, performing long-term experiments using the same catalysts for the same reaction, found that the shape selectivity was lost at some point during the reaction due to the amorphisation of the MOF under reaction conditions.^69^

With the discovery of more stable MOFs such as the MIL-100 and MIL-101 solids, their application in catalysis became more feasible.^5^,^35^–^38^,^70^ These materials display exceptional stability and large pores, which are desired features for catalytic applications.^4^,^5^,^71^–^73^ Férey and co-workers published the first example of catalysis with the MIL-100 and MIL-101 families.^71^ In this case, they focused on the chromium-based material MIL-101(Cr) and its application in the catalytic oxidation of sulphides using hydrogen peroxide. Following this work, many other publications using these families of materials appeared in the field of hydrocarbon oxidation^74^,^75^ and Lewis acid catalysed reactions.^76^,^77^

Regarding oxidation reactions, it is important to remark that when only MOFs were used as catalysts and neither co-catalysts nor promoters were added, molecular oxygen could not be used as oxidant. Indeed, most OMSs are not able to activate dioxygen under mild reaction conditions.^75^,^78^,^79^ Very recently, however, Llabrés i Xamena and co-workers showed that MIL-100(Fe) treated under the appropriate conditions before reaction generates a redox pair, Fe^3+^/Fe^2+^, that assists with the generation of peroxides directly from oxygen.^5^,^80^ For more information about MOFs used in oxidation reactions, we refer the reader to the recent review by Dhakshinamoorthy *et al.* and to the extensive characterisation on the MIL-100 and MIL-101 families performed by Daturi and co-workers.^72^,^81^–^86^

As can be expected, the metal node on which the catalysis takes place has a clear influence on the Lewis acidity of the corresponding MOF. As reported by Mitchell *et al.*, MIL-100(Sc) outperformed the catalytic behaviour of OMSs-containing MIL-100(Cr), MIL-100(Fe), HKUST-1, and CPO-27(Ni) in several Lewis-catalysed reactions such as Friedel–Crafts and Michael addition reactions.^87^ In spite of lacking coordination vacancies in their ideal crystalline structure, dehydration of the [Zr_6_O_4_(OH)_4_]^12+^ SBUs to [Zr_6_O_6_]^12+^ creates μ_3_ vacancies that, together with the occurrence of crystalline defects associated to linker deficiencies, introduce highly desired Lewis acid properties.

**Fig. 3:**
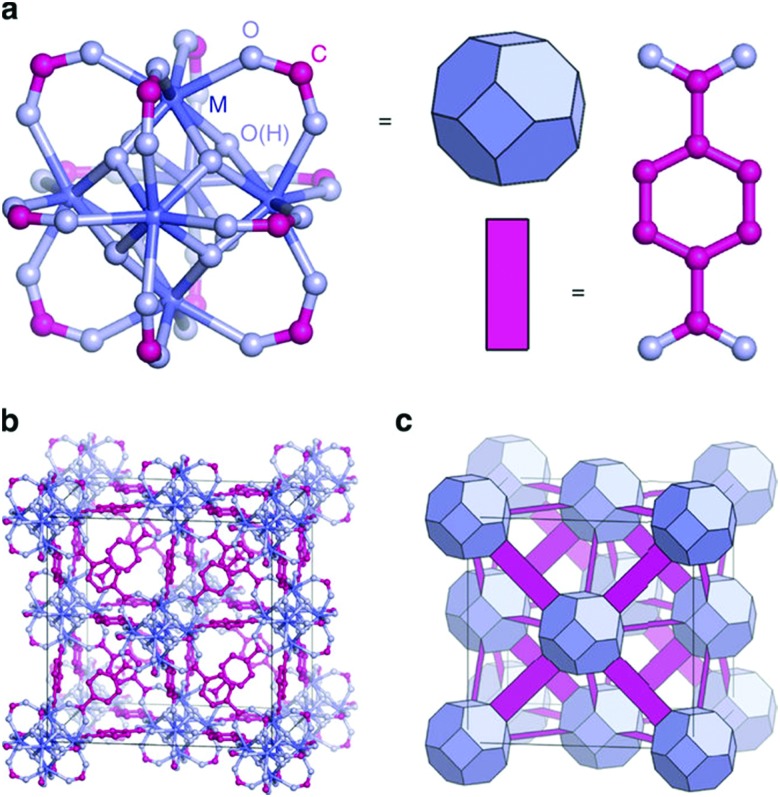
Structural description of UiO-66. (a) Metal cluster, (b) **fcu** topology, (c) simplified representation of the same topology. Reproduced from ref. 88 with permission from the Nature Publishing Group, copyright 2014.

The second breakthrough in the use of MOFs with OMSs was the discovery that UiO-66(Zr), depicted in Fig. 3, may contain a high density of OMSs depending on the synthetic procedures, while retaining its stability.^48^,^89^,^90^ Vermoortele *et al.* firstly used UiO-66 for the synthesis of jasminaldehyde through the condensation of heptanal and benzaldehyde, and the conversion was found to be directly related to the activation procedure.^91^ The authors observed a clear correlation between the degree of dehydration and the attained conversion levels. Following this work, the same authors reported a positive effect of the electron-withdrawing groups in the organic linker on the catalytic activity of the metal nodes during the cyclisation of citronellal.^92^ The reaction was strongly enhanced by incorporating electron-withdrawing groups (F,Cl,Br) in the linkers. A similar finding was published by Timofeeva *et al.* for the acetylisation of benzaldehyde.^93^

Cirujano *et al.* used UiO-66(Zr) and its amino-functionalised version as catalysts for biomass related esterification reactions,^94^ observing the activity of the amino-functionalised material to be higher than that of the nonfunctionalised UiO-66(Zr). They ascribed this unexpected behaviour to the role of the amino group in the activation of the alcohol, inferring a bifunctional acid–base mechanism to explain the improvement in the reaction rate. This reactivity trend was also found in CO_2_ cycloaddition and cross aldol condensation.^91^,^95^ The same authors reported that a direct correlation exists between the amount of missing linker defects and the catalytic activity of UiO-66 materials for the acid-catalysed esterification of levulinic acid with various alcohols, thus evidencing the importance of such type of defects in creating suitable OMSs to catalyse this reaction (*vide infra*).^94^

The UiO-66 structure was also synthesised with cerium as the metal species. Ebrahim *et al.* discovered in 2013 that UiO-66(Zr) could be doped with Ce(iii) atoms following a one-pot synthesis approach.^96^ The prepared materials were used for NO_2_ adsorption and they demonstrated the importance of the presence of Ce(iii) in the structure. A few years later, Nouar *et al.* prepared cerium-doped UiO-66(Zr) by post-synthetic metal exchange and Lammert *et al.* were able to prepare pure UiO-66(Ce).^97^,^98^ A thorough X-ray absorption near edge structure (XANES) analysis demonstrated a iv oxidation state of cerium in this MOF. When used in the aerobic oxidation of benzyl alcohol, the MOF was only active in the presence of a co-catalyst (TEMPO, (2,2,6,6-tetramethylpiperidin-1-yl)oxyl). The authors reported a large influence of the activation conditions on the catalytic performance, meaning that OMSs play an important role in this process. Recently, Janiak and co-workers have reported the use of UiO-66(Ce) for epoxidation reactions. Their results are in line with those discussed above. The MOF could not activate oxygen and, consequently, the oxidation did not take place, so *tert*-butylhydroperoxide (TBHP) was added as oxidant.^99^

### B.2. Metal nodes as anchoring sites of single-site catalysts

OMSs are electron-deficient centres, so they are prompt to interact with electron-rich substituents. In this way, Férey and co-workers used a grafting technique to functionalise MOFs with additional active species. The OMSs of MIL-101(Cr) were functionalised with ethylene diamine, on which palladium nanoparticles were immobilised to be applied in coupling reactions.^4^ Mondloch *et al.* used NU-1000 (NU = Northwestern University), which consists of Zr_6_ or Hf_6_ nodes [M_6_(μ_3_-O)_4_(μ_3_-OH)_4_(OH)_4_(H_2_O)_4_, M = Zr, Hf] and the tetracarboxylate linker 1,3,6,8-tetrakis(*p*-benzoate)pyrene (H_4_TBAPy), bearing –OH and –OH_2_ groups prone to immobilise active species.^100^ The authors immobilised an electrophilic organozirconium catalyst for the polymerisation of ethylene and 1-hexene. For these reactions, an acid co-catalyst or initiator is normally needed; however, the use of NU-1000(Hf) could obviate their presence. Density functional theory calculations showed that the active zirconium sites were highly polarised due to the interaction with the hafnium inorganic node (*vide infra*), resulting in very electrophilic zirconium sites able to coordinate, initiate, and propagate the polymerisation reaction.

Dincă and co-workers prepared a number of MFU exchanged MOFs with outstanding activity for the oligomerisation of ethylene by post-synthetic cation exchange.^101^ However, the use of an initiator was required in this case. Another excellent example of truly single-site catalysts making use of post-synthetic cation exchange by the group of Dincă is the immobilisation of Fe^2+^ in MOF-5(Zn) and its application in the disproportionation of nitric oxide.^102^

**Fig. 4:**
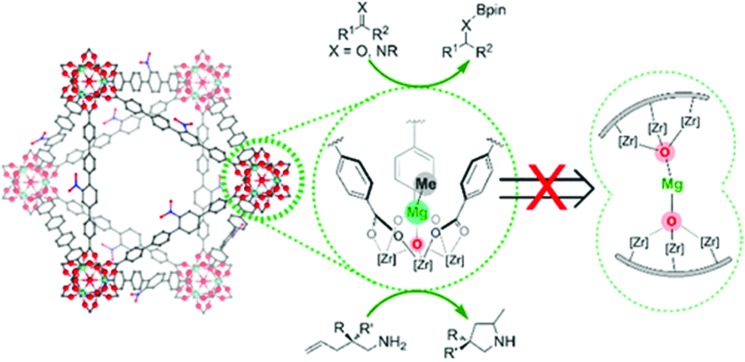
Representation of a magnesium alkyl complex immobilised on the inorganic cluster of a UiO-66-based MOF. Reproduced from ref. 103 with permission from the American Chemical Society, copyright 2016.

Manna *et al.* prepared UiO-68(Zr) and used the μ_3_-OH groups to attach a molecular catalyst.^103^ Firstly, they deprotonated the –OH groups using *n*BuLi, followed by reaction with CoCl_2_ or FeBr_2_·2THF (THF = tetrahydrofurane). The prepared catalysts were used in benzylic C–H silylation and benzylic C–H borylation. The authors used the extended X-ray absorption fine structure (EXAFS) technique to check the low coordination number of the cobalt complex immobilised in the MOF, and proved that it was isolated. The same group used a similar methodology to generate a single-site catalyst based on the UiO-66 topology and a magnesium alkyl complex, see Fig. 4.^103^ The resulting material showed high activity in the hydroboration of carbonyls and imines, the hydroamination of aminopentenes, and ketone hydroboration. They found that the low coordination of the immobilised metal generated extremely electrophilic centres that can activate molecules without the need of a co-catalyst.

A similar approach was followed by these authors to prepare cerium hydride single sites in MOF-808(Ce). The hydroxyl groups of the metal nodes were made to react with different organic reagents until they could prepare Ce(iii) hydride, the presence of Ce(iii) atoms being revealed by EXAFS. This material was used in several reactions. For example, they found a unique 1,4-regioselectivity for the hydroboration of pyridine, and traced back its origin to steric effects favoured by the MOF structure.^104^

One last powerful approach to anchor single sites to the metal nodes of different MOFs has been recently reported by the group of Farha and relies on the well-known atomic layer deposition (ALD) technique. Especially interesting is the generation of single nickel sites that were later used in the hydrogenation of ethylene. This catalyst behaved very similarly to nickel nanoparticles in terms of activation procedure and deactivation. However, the turnover frequency (TOF, expressed per atom of nickel present in the catalyst) was one order of magnitude higher than that found for nickel supported on ZrO_2_.^100^,^103^

### B.3. Catalysis on lattice defects

An infinite periodic repetition of identical groups of atoms in space does not exist, since real crystals are usually far from perfection. In the case of MOFs, these defects can be classified as (i) local defects (vacancies of either linkers or metal nodes), (ii) line defects (dislocations), (iii) planar defects (grain boundaries) and (iv) voids (empty spaces in the crystal). Along this line, Sholl *et al.* suggested a simpler classification by distinguishing point defects from extended defects.^105^ The first one is associated with simple vacancies in the crystal, while the second one defines two-dimensional imperfections all along the crystal. Recently, Fang *et al.* proposed another classification based on the origin of the defect: (i) inherent defects and (ii) engineered defects.^106^ The difference is that in the first case the presence of the defect cannot be avoided or controlled and is generated during the synthesis, while in the second case the defects are generated on purpose. Independent of the nature and location of these defects, they all can act as single catalytic sites.

#### B.3.1. Surface defects

Surface defects appear at the termination points of a crystal. One of the first examples of catalysis at these defects in a MOF was published in 2010 by Chizallet *et al.*^25^ These authors demonstrated that ZIF-8 (ZIF = zeolitic imidazolate framework) was active in the base-catalysed transesterification of fatty acids with alcohols, even when large molecules were involved. Such catalytic activity was explained based on a large number of surface-terminated imidazole groups. The catalytic activity correlated well with particle size, as demonstrated later by Schneider and co-workers.^107^

Another interesting approach to generate or modify the type of defects at the crystal surface is the one published by Chen *et al.*^108^ They prepared ZIF-8 with different morphologies, rhombic dodecahedra and nanocubes, and applied these materials in a Knoevenagel condensation. Nanocube-shaped ZIF-8 crystals surpassed the catalytic behaviour of rhombic dodecahedral particles. The authors ascribed these results to a higher density of Zn^2+^ on the faces of the crystal.

Wee *et al.* reported a protocol to produce hierarchical porosity in ZIF-8 crystals.^109^ In that way, more “external” surface of the ZIF-8 would be exposed to the reactants and the ZIF-8 material could be more efficiently utilised. Ramos-Fernandez *et al.* described the immobilisation of MIL-101(Cr) in cordierite monoliths, and the MOFs monoliths were used in selective oxidation.^43^ Aguado *et al.* reported the immobilisation of SIM-1, a substituted imidazolate-based MOF, on alumina beads and its application in a ketone transfer hydrogenation.^110^

#### B.3.2. Bulk defects

Defect engineering, as defined by the groups of Fischer and Farrusseng,^106^,^111^ is a powerful approach to maximise the amount of defects within a given MOF crystal. This can be done either directly during the synthesis of the material or by following different post-synthetic approaches.

##### Defects created during the synthesis

A simple way to generate defects was proposed by Ravon *et al.* in 2008.^112^ This method involves the use of a synthetic approach that allows the MOF precursors to react very fast. This rapid nucleation induces a number of defects in the lattice, such as missing linkers, leading to a high concentration of unsaturated metal centres with acid properties, similar to OMSs. Llabrés i Xamena *et al.* used the same concept to prepare IRMOF-3 (IRMOF = isoreticular MOF) with improved activity in Knoevenagel condensations, originating in part from the inclusion of small ZnO impurities during the synthesis of the MOF which contributed to the observed catalytic activity.^113^ A second approach followed by Ravon *et al.* was the addition of monodentate linkers (“*dummy linkers*”) to the synthesis of MOFs made from polydentate linkers, producing local defects (“*truncated missing linkers*”) at the metal clusters.^112^

Vermoortele *et al.* used the same approach to obtain defected UiO-66(Zr) by using trifluoroacetic acid (TFA) to modulate the synthesis.^114^ The addition of TFA produced a large number of defects, since part of the terephthalic acid linkers were replaced by the monodentate modulator. After thermal activation, the modulator was removed and defects were generated. They finally used the defected UiO-66(Zr) for catalysing the Meerwein reduction of 4-*tert*-butylcyclohexanone with isopropanol and the citronellal cyclisation. They found that the catalytic activity increased when TFA was removed, since extra Lewis acid sites were formed. In both reactions, they found that TFA addition strongly benefits the catalytic activity. While regular UiO-66 reaches a conversion of 34% after 10 h, the defected UiO-66 converted almost 75% of citronellal after the same time. Even more pronounced was the effect of TFA addition when the defected UiO-66 was used in the Meerwin reduction. While the undefected MOF only reached a 5% conversion after 24 hours of reaction, the UiO-66 modified with TFA reached a 93% conversion.

Kozachuk *et al.* further improved the strategy of Farrusseng, and, instead of using a “*dummy linker*” having one linking carboxylate moiety less than the proper linker, they used a linker in which one of the carboxylic groups was replaced by another coordination site (*e.g.* a pyridine group instead of a carboxylic one).^115^ This produced a change in the coordination number of the metal cluster, hence creating a defect that modified its activity. Marx *et al.* used this approach to produce defected HKUST-1 where some of the trimesic acid linkers were substituted by 2,5-pyridinedicarboxylate (PyDC).^116^ This substitution produced a decrease in the coordination number of the copper atoms in the clusters, which generated a redox Cu^2+^/Cu^+^ pair in the paddlewheel nodes. When this material was used in the oxidation of toluene, a conversion of 3% could be reached, while regular HKUST-1 only reached a conversion of 0.3%. Even though the achieved activities were not overwhelming, this approach to introduce redox functionalities in a MOF is noteworthy. The ruthenium version of the same MOF was prepared with a similar methodology.^115^ A very similar behaviour was obtained: when trimesic acid linkers were substituted by PyDC linkers, the coordination number of the ruthenium atoms was also decreased, producing a Ru^+^/Ru pair, which is well-known as an efficient catalyst in hydrogenation reactions.

##### Post-synthetic defect generation

Post-synthetic defect generation involves the introduction of defect sites after the construction of the MOF. One of the first examples of this approach was reported by Rosseinsky and co-workers.^117^ They prepared amino-acids open frameworks based on l-aspartate, 4,4′-dipyridyl, and nickel clusters. After the synthesis, they treated the material with HCl solutions to protonate the structure. Once the MOF was protonated, it was used as a catalyst in the methanolysis of *cis*-2,3-epoxybutane showing activity and an enantiomeric excess (ee) of 10.

Vermoortele *et al.* developed a post-synthetic route to damage MOFs in a controlled way, generating both Lewis and Brønsted acid sites.^76^ MIL-100(Fe) is well known for its high number of OMSs and its activity in Lewis acid catalysed reactions as well as oxidation reactions. In order to generate Brønsted acid sites, these authors treated MIL-100(Fe) with TFA and perchloric acid. They found that this treatment produced a modification of the iron oxo-cluster. A new coordination site was opened in the iron octahedron, and a carboxylic group was also liberated, resulting in MOFs with two isolated single sites in close proximity. The resulting material was used in the isomerisation of α-pinene oxide and the cyclisation of citronellal. A clear correlation was observed between the number of defects and the catalytic activity. Finally, the catalyst was also used in Diels–Alder reactions between different dienophiles and 1,3-cyclohexadiene.

## C. Single-site catalysis in covalent organic frameworks and porous organic polymers

### C.1. Nomenclature

As discussed above, the term porous organic framework (POF) involves a number of porous solids based only on organic constituents, encompassing covalent organic frameworks (COFs) and porous organic polymers (POPs). POFs possess high surface areas, tuneable pore sizes, and adjustable skeletons that offer unprecedented possibilities for the design of single-site catalysts. If the organic constituents are aromatic, the term porous aromatic framework (PAF) is adopted.

COFs were pioneered by the group of Yaghi and are highly crystalline solids, originally synthesised *via* the reversible formation of boroxine rings. The simplest example of this class of materials is COF-1, obtained by the self-condensation of benzene-1,4-diboronic acid.^118^ It has a Brunauer–Emmett–Teller (BET) surface area of 711 m^2^ g^–1^ and an average pore size of 0.7 nm. COFs can also be constructed *via* the co-condensation of two or more building blocks. This allows constructing COFs with different properties and functions. However, the application of the aforementioned COFs is often limited since these COFs based on boroxine rings are not stable in water.^119^ It has to be noted that the term COF is currently being used not only for materials containing boroxine rings, but also to describe every crystalline porous organic framework irrespective of its building units. For instance, the vast majority of imine-linked polymers, prepared by the co-condensation of aromatic aldehydes with amines, are amorphous networks. However, by varying the synthetic conditions, the crystalline form of the material can be obtained. Along this line, the group of Yaghi reported the synthesis of COF-300, a crystalline imine-linked polymer prepared by the co-condensation of the tetrahedral building block tetra-(4-anilyl)methane with the linear terephthaldehyde linking unit.^120^ The group of Dichtel further explored the crystallisation of amorphous imine-linked polymer networks to generate 2D COFs.^121^ It was shown that COF formation occurs through the initial rapid precipitation of an amorphous imine-linked network with a low surface area that crystallises into a COF over days under conditions facilitating imine exchange. Hence, reversible condensation reactions are essential in COF synthesis.

**Fig. 5:**
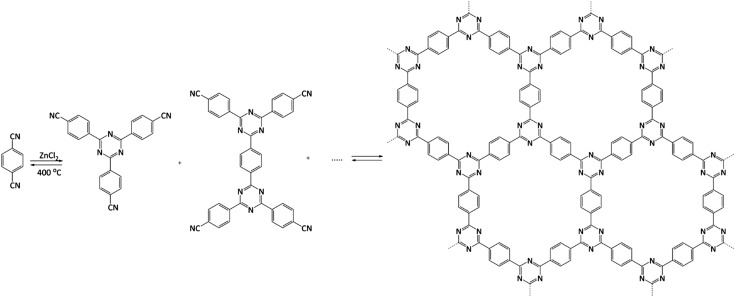
Structure of CTF-1. Adapted from ref. 122.

From the catalytic point of view, covalent triazine frameworks (CTFs) are more interesting materials. CTFs are porous aromatic frameworks made upon the trimerisation of aromatic nitriles. The first reported triazine framework, CTF-1 (Fig. 5), was prepared from 1,4-dicyanobenzene, and the structure is isoelectronic to COF-1. However, CTF-1 outperforms COF-1 in terms of both thermal and chemical stability.^122^ CTFs are normally prepared using an excess of molten ZnCl_2_ as both solvent and catalyst for the polymerisation. However, Ren *et al.* reported an alternative synthetic procedure using triflic acid as a catalyst during room-temperature and microwave-assisted synthesis.^123^

Networks containing triazine rings can be synthesised by other methods as well. For instance, the group of Müllen reported on the synthesis of porous polymers through Schiff base chemistry by the condensation of melamine with different di- and trialdehydes.^124^ Another example was reported by Grate *et al.* and consists of the conversion of cyanuric chloride to side-chain functionalised polymers.^125^

Another interesting class of POFs are the so-called hypercrosslinked polymers (HCPs). This is a large class of polymers firstly introduced by Davankov in 1969.^126^ HCPs are typically synthesised from linear or low cross-linked polyarylates or polysulphones using a post-crosslinking agent *via* the Friedel–Crafts reaction.^127^,^128^

Conjugated microporous polymers (CMPs) are networks built from multiple carbon–carbon bonds and aromatic rings in a π-conjugated fashion. The conditions required for their synthesis are milder than in the case of HCPs or CTFs, which allows the inclusion of a wide range of functionalities. CMPs are obtained *via* different types of carbon–carbon coupling reactions such as Sonogashira coupling,^129^ Yamamoto coupling,^130^ Suzuki–Miyaura coupling,^131^ or cobalt-132 or palladium-catalysed^133^ homocoupling of di- or tri-alkynes. In 2008, the group of Cooper reported on the synthesis of several CMPs obtained *via* Sonogashira–Hagihara coupling.^133^ In 1994, Wang *et al.* reported three-dimensional organic networks with zeolitic properties by replacing carbon atoms within the framework by silicon and tin.^134^ Later, Kaskel and co-workers introduced the term elemental organic framework (EOF), a type of CMPs containing Si, Sn, Sb, or Bi.^135^,^136^

A last class of POFs are the so-called polymers of intrinsic microporosity (PIMs), pioneered by McKeown and Budd.^137^,^138^ PIMs are polymers possessing a rigid backbone that prohibits any free rotation around itself and are made *via* non-reversible condensations, which result in infective packing of the polymer. Porosity in PIMs stems from bent monomers possessing a so-called “site of contortion”, usually a tetrahedral carbon atom. In other words, PIMs do not require a network of covalent bonds to exhibit microporosity; appropriate free volume is trapped within the network due to their irregular, twisted backbones.^139^

### C.2. Bottom-up POF-based catalysts

This pre-synthetic strategy is often preferred, since it allows for the distribution of a high loading of functional groups or catalytic sites in a very homogeneous manner over the framework.

#### C.2.1. Bottom-up metal-containing POF-based catalysts

In 2010, the group of Jiang described the synthesis of CMPs using an iron metalloporphyrin building block *via* Suzuki coupling.^140^ The obtained FeP-CMP catalyst was employed for the activation of molecular oxygen under ambient conditions to convert sulphide to sulphoxide. The catalysts showed activity with a broad range of substrates showing a large turnover number (TON of 97 320 for the oxidation of thioanisole after 40 h) and up to 99% conversion. Three years later, the same group described the synthesis of a CuP-SQ catalyst, a crystalline porous polymer obtained *via* the condensation of squaric acid (SQ) and copper(ii) 5,10,15,20-tetrakis(4-aminophenyl)porphyrin.^141^ The CuP-SQ COF was tested as a photocatalyst in the oxygen evolution reaction. The extended π-conjugation, due to the presence of squarine building blocks, improved the light harvesting capacity and lowered the band gap compared to the porphyrin monomer.

The group of Chang presented COF-366-Co and COF-367-Co as catalysts for the electrochemical reduction of CO_2_ to CO in water.^142^ The frameworks are built by imine-condensation of 5,10,15,20-tetrakis(4-aminophenyl)porphinato cobalt and 1,4-benzenedicarboxaldehyde or 4,4′-biphenyldicarbaldehyde. The catalyst exhibited a high faradaic efficiency (90%) and turnover numbers up to 290 000. Singh *et al.* described the synthesis and application of another porphyrin-containing network prepared *via* the condensation of 5,10,15,20-tetrakis(4-aminophenyl) iron or manganese porphyrin with perylene-3,4,9,10-tetracarboxylic dianhydride.^143^ These materials were employed in the selective oxidation of alkanes and alkenes with *tert*-butyl hydroperoxide.

Jiang *et al.* prepared CMP-based catalysts where bipyridine and phenylpyridine complexes of rhenium, rhodium, and iridium were incorporated into a framework *via* Sonogashira–Hagihara cross-coupling.^144^ Two different metal–organic conjugated microporous polymers (MOP-CMPs) were synthesised from two different preformed metal–organic monomers – bi- and tetra-functional with respect to the Sonagashira–Hagihara reaction. Bonding patterns in this case resemble those of MOFs, where the metal atoms are integral nodes in the network structure.

Li *et al.* described the synthesis of metallosalen microporous organic polymers (MsMOP-1) with palladium–salen building blocks.^145^ The framework was employed as a catalyst (Pd loading of 5.01%) in the Suzuki–Miyaura and Heck coupling for a range of substrates; it showed high activity and good recyclability: the model reaction using iodobenzene and phenylboronic acid showed a yield of 99% and was repeated five times without any significant loss of activity. Another example of using a salen complex as a building block was reported by the group of Deng.^146^,^147^ They prepared Co- and Al-coordinated CMPs capable of capturing and converting CO_2_ to propylene carbonate at room temperature and atmospheric pressure.^146^ When co-catalysed with tetra-*n*-butylammonium bromide (TBAB), a quaternary ammonium salt, Co-CMP and Al-CMP showed a superior catalytic activity compared to the corresponding homogeneous catalyst – with a homogeneous salen–Co-OAc TONs of 158 were obtained, while Co- and Al-CMP showed TONs of 201 and 187, respectively. The higher activity of the heterogeneous system was explained by the enriched CO_2_ capture ability of Co(Al)-CMP and, therefore, the higher local concentration of CO_2_ within the polymer. Co-CMP was recycled 22 times without a significant loss of activity, while with Al-CMP the reaction yields dropped from 78.2% to 51.3% after only three times. Trace water in the system may have formed inactive Al species due to the highly hygroscopic nature of salen–Al.^146^ Later, they synthesised the chromium implanted network Cr-CMP, which was used to capture CO_2_ and consequently catalyse its cycloaddition to epoxides forming cyclic carbonates.^147^ The catalyst showed a superior activity compared to its homogeneous counterpart (TOFs of 224 h^–1^ for Cr-CMP *versus* 167 h^–1^ for the homogeneous salen–Cr–Cl) and was reused more than ten times without a significant loss in activity.

Wang *et al.* reported a series of porous organic polymers prepared *via* Sonogashira chemistry from N-heterocyclic carbine gold(i) and alkynes of different chain length.^148^ These frameworks were tested in the alkyne hydration reaction for a range of substrates.

The Kaskel group presented EOFs based on tin (EOF-3), antimony (EOF-4), and bismuth (EOF-5) atoms as heterogeneous catalysts for the cyanosilylation of benzaldehyde.^149^ All three networks exhibited a good stability and catalytic activity. The heterogeneity of the reaction was proven by filtration tests. Wee *et al.* also used the Sn-EOF, this time as a catalyst for the esterification of oleic acid with glycerol.^150^ It outperformed several MOFs, which were also tested under the same conditions, in terms of stability and catalytic performance, achieving >98% selectivity towards monoglyceride and a conversion of 40%.

#### C.2.2. Bottom-up metal-free POF-based catalysts

Du *et al.* described the synthesis of a microporous polymer containing a covalently bonded Tröger's base.^151^ The network was constructed *via* the Sonogashira–Hagihara coupling reaction and has a BET surface area of 750 m^2^ g^–1^. The material was tested as a catalyst for the addition of diethylzinc to 4-chlorobenzaldehyde. The catalyst showed a comparable activity to homogeneous Tröger's-base derivatives with no appreciable decrease in activity after three runs.

**Fig. 6:**
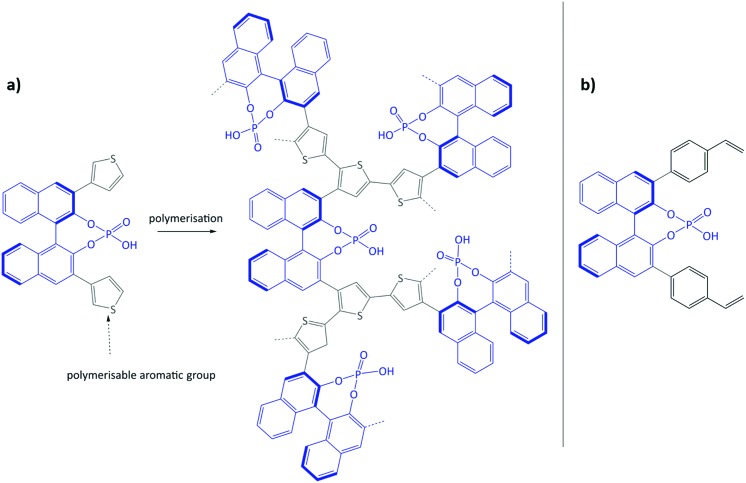
(a) Concept of immobilisation of BINOL-derived phosphoric acid *via* the oxidative coupling of thiophenes; (b) BINOL building block for copolymerisation with styrene and divinylbenzene. Figure adapted from ref. 152 and 153.

Using the same bottom-up approach, Thomas and co-workers introduced chirality into a fully organic framework.^152^ A chiral 1,1′-bi-2-naphthol scaffold (BINOL) was used as tecton in order to introduce enantioselectivity into a desired catalyst (see Fig. 6). BINOL was chosen because of its structure-directing function and, on top of that, its corresponding phosphoric acid is well-known as an important organocatalyst. The catalyst was applied in the transfer hydrogenation of dihydro-2*H*-benzoxazine. It showed an increased enantioselectivity in comparison to the homogeneous reaction, from 34% to 56% ee. Recycling of the catalyst showed no indication of leaching. In the follow-up work, the same catalyst was tested for the asymmetric hydrogenation of 3-phenyl-2*H*-1,4-benzoxaine, a set of 2-aryl quinolones, and the asymmetric Friedel–Crafts alkylation of pyrrole, showing a high activity and selectivity in all cases.^154^ The group of Theissmann also employed a BINOL building block to build an organic network using a different approach, where the precursor was copolymerised with styrene and divinylbenzene.^153^

Cho *et al.* described the preparation of a tube-shaped microporous organic network bearing imidazolium salt (T-IM), prepared through the Sonogashira coupling of tetrakis(4-ethylphenyl)methane and a diiodoimidazolium salt.^155^ Rose *et al.* used a similar imidazolium linker to prepare a cross-linked EOF by Suzuki–Miyaura coupling.^136^ The carbon- and silica-based EOFs were tested in the conjugate umpolung of α,β-unsaturated cinnamaldehyde and trifluoroacetophenone. The catalysts showed similar results compared to molecular species used as homogeneous catalysts.

Suresh *et al.* reported the synthesis of an amide-functionalised microporous organic polymer (Am-MOP) constructed from trimesic acid and *p*-phenylenediamine.^156^ The framework allowed for a highly selective CO_2_ uptake over other gases, since its pore surface is very polar. It also showed a catalytic activity in the Knoevenagel condensation of aldehydes and methylene compounds. The group of Zhao recently described the synthesis of porous polymers bearing functional quaternary ammonium salts by the copolymerisation of divinylbenzene and hydroxyl-functionalised quaternary ammonium salts, displaying excellent catalytic performance in the synthesis of cyclic carbonates from epoxides and CO_2_.^157^

Wang *et al.* presented a robust chiral porous polymer (JH-CPP) with embedded Jørgensen–Hayashi catalysts.^158^ JH-CPP was synthesised by the Co_2_(CO)_8_-mediated trimerisation of tetrahedrally structured building blocks and showed a high activity in catalysing the asymmetric Michael addition of aldehydes to nitroalkenes, achieving a good to excellent yield (67–99%), high enantioselectivity (93–99% ee), and high diastereoselectivity (diastereomeric ratio of 74 : 26 to 97 : 3). The catalyst was reused four times without loss of selectivity.

A sulphonated crystalline network, TFP-DABA, was reported by Peng *et al.*^159^ The framework was prepared *via* the Schiff base condensation of 1,3,5-triformylphloroglucinol and 1,5-diaminobenzenesulphonic acid, followed by irreversible enol-to-keto tautomerisation. TFF-DABA was studied as an acid catalyst in the dehydration of fructose to 5-hydroxymethylfurfural (HMF) and, if KBr was employed as a co-catalyst, to 2,5-diformylfuran (DFF). It exhibited 97% and 65% yield for HMF and DFF respectively, combined with a good chemoselectivity.

Saptal *et al.* reported the synthesis of two catechol porphyrin COF catalysts for the chemical fixation of carbon dioxide *via* cyclic carbonates and oxazolidinones. The COFs were synthesised *via* a Schiff base reaction using 2,3-dihydroxyterephthalaldehyde (2,3-DhaTph) or 2,3-dimethoxyterephthalaldehyde (2,3-DmaTph) units.^160^

### C.3. Top-down POF-based catalysts

#### C.3.1. Top-down metal-containing POF-based catalysts

In 2011, the group of Wang described the application of an imine-linked COF (COF-LZU1, LZU = Lanzhou University) as a support for palladium complexes.^161^ Simple post-treatment of COF-LZU1 resulted in catalysts with robustly incorporated Pd(OAc)_2_ with a palladium content of 7.1 ± 0.5 wt%. The crystallinity of the framework was fully preserved after the post-functionalisation, and the coordination of the palladium to the nitrogen atoms of the framework was confirmed by XPS (X-ray photoelectron spectroscopy) and ^13^C CPMAS NMR (cross polarization magic angle spinning nuclear magnetic resonance). The catalyst exhibited a high activity in the Suzuki–Miyaura coupling of a broad range of aryl-halides with phenylboronic acid, showing excellent yields and a high stability – when the catalyst was tested in the cross-coupling of *p*-nitrobromobenzene and phenylboronic acid, the yield remained 97% after the fourth cycle. The tolerance of COF-LZU1 to relatively harsh conditions was also verified.

Li *et al.* prepared a microporous knitting aryl network (KAP) with a high surface area *via* the knitting of triphenylphosphine (PPh_3_) with benzene.^158^ Further binding of the PPh_3_ groups with PdCl_2_ produced KAPs(Ph-PPh_3_)-Pd catalysts with 0.6 mol% of palladium. The frameworks enabled the efficient dispersion of palladium within its structure. The presence of PPh_3_ functional groups and the incorporation of palladium was confirmed by FTIR (Fourier transform infrared spectroscopy), solid state ^13^CPMAS and ^31^P HPDEC NMR (high power decoupling NMR), and XPS techniques. KAPs(Ph-PPh_3_)-Pd exhibited excellent activity and selectivity in the Suzuki–Miyaura cross-coupling reaction of aryl chlorides. Later, the same group reported a cost-effective approach to prepare microporous organic polymers *via* the Scholl reaction.^162^ The approach involves the elimination of two aryl-bonded hydrogen atoms accompanied by the formation of a new aryl–aryl bond in the presence of a Friedel–Crafts catalyst. A series of polymers was prepared by varying the starting building blocks. The frameworks named SMP-8a and SMP-9a (SMP = Sholl-coupling microporous polymer), both prepared from *sym*-PhPh_3_, PPh_3_, and bipyridine, were analysed as catalyst supports. The SMP-8b catalyst (Pd loading of 1.2 wt%), obtained by treating the SMP-8a framework with PdCl_2_, showed a high activity for the Suzuki–Miyaura coupling reaction (TOFs up to 59 400 h^–1^) using water–ethanol mixture as a solvent. The superior activity of the SMPs-8b catalyst was attributed to its unique microporous structure and to the abundance of highly dispersed PPh_3_ groups stabilizing the palladium species and preventing aggregation.

Wang and co-workers described the synthesis of two urea-based porous organic frameworks, UOF-1 and UOF-2, synthesised *via* the condensation of 1,3,5-benzenetriisocyanate with 1,4-diaminobenzene and benzidine, respectively.^163^ The palladium-containing catalysts, Pd^II^/UOF-1 and Pd^II^/UOF-2 (16.87 and 16.83 wt% of Pd, respectively), were obtained by treating the pristine polymers with [Pd(OAc)_2_]. The coordination of the Pd^II^ species was confirmed with ^13^C CPMAS NMR and XPS. Both catalysts showed a high catalytic activity in Suzuki–Miyaura coupling in water for a large range of substrates. Pd^II^/UOF-1 showed a slight loss in catalytic activity in the fourth reaction run, whereas the reactivity of Pd^II^/UOF-2 decreased after the third run. Both Pd^II^/UOF-1 and Pd^II^/UOF-2 were also tested for the reduction of nitroarenes. Pd^II^/UOF-1 did not show any drop in catalytic activity through four reaction runs, but the selectivity had dropped. However, the activity and selectivity of Pd^II^/UOF-2 dropped only in the fifth catalytic run; Pd^II^/UOF-2 was tested for a range of nitro compounds, its superior activity over Pd^II^/UOF-1 was not investigated. TEM (transmission electron microscopy) and XPS analysis of the spent catalysts demonstrated that Pd^II^ was partially reduced to Pd^0^ and well-dispersed metal nanoparticles were formed after the first run of a reaction.

The group of Iglesias synthesised functionalised porous polyimides (PPI-*n*) prepared by the condensation of aromatic amines with pyromellitic dianhydride.^164^ The frameworks were functionalised with amino groups (PPI-*n*-NH_2_). First, the nitration was performed, followed by the reduction of the nitro groups by SnCl_2_·2H_2_O in THF. The incorporation of palladium was performed in two steps. First, the amino-functionalised frameworks reacted with picolinaldehyde to yield the iminopyridine ligands (PPI-*n*-NPy). After, these derivatives reacted with bis(benzonitrile)palladium(ii)chloride. The catalysts (Pd loading of 3.42 and 1.76%) were tested in Suzuki coupling in pure water and showed a high activity for a range of substrates, while their heterogeneous nature was confirmed by hot filtration tests. However, ICP (inductively coupled plasma) analysis for one of the reused palladium-functionalised frameworks demonstrated that 20% of the palladium was lost after seven runs.

Hou *et al.* presented a nitrogen-rich COF built up from 5,10,15,20-tetra(*p*-amino-phenyl)porphyrin and 4,4′-biphenyldialdehyde.^165^ The periodically distributed nitrogen atoms allowed to uniformly disperse palladium ions. To prepare the catalyst, Pd(OAc)_2_ was used; the palladium loading was found to be 12.87% and its coordination was confirmed by XPS and ^13^C CP/TOSS NMR (TOSS = total suppression of spinning sidebands). The catalyst showed a high activity in Suzuki–Miyaura coupling reactions with good selectivity and yields. The hot filtration test indicated the heterogeneous nature of the catalyst. TEM analysis of a spent catalyst did not reveal any obvious aggregates or change in morphology. Leaching of palladium was below the detection limit of ICP.

Bruijnincx and co-workers developed a series of 4,4′-biphenyl/phosphine-based amorphous frameworks.^166^ Palladium coordination to phosphorous atoms was achieved by using the Pd(acac)_2_ precursor, while Pd(dba)_2_ led to the formation of Pd^0^. The coordination was confirmed with ^31^P NMR and DRIFTS spectroscopy. The catalyst was tested in the telomerisation of 1,3-butadiene with phenol (catalysts with 0.02–0.16 wt% of Pd were studied) and glycerol (the employed catalysts had 0.056–0.115 wt% of Pd). A high activity and selectivity were obtained under solvent- and base-free conditions, and in the case of glycerol telomerisation, the catalyst outperformed its homogeneous analogue PPh_3_. It was possible to increase the selectivity by increasing the ligand-to-metal ratio, which also reduced the metal leaching.

**Fig. 7:**
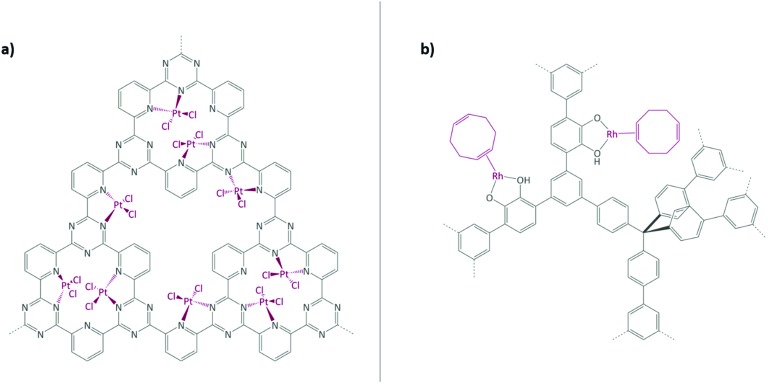
(a) Periana catalyst immobilised on CTF; (b) Rh(i) complex immobilised on a catechol-containing POP. Figures adapted from ref. 167 and 170.

Schüth and co-workers have reported one of the most impressive catalysis to date in a COF paper by immobilising the well-known Periana catalyst using a CTF as support (Fig. 7(a)).^167^–^169^ K_2_[PtCl_4_] was chosen as the platinum precursor and its successful coordination to the bipyridine moieties within the CTF was confirmed by XPS. Catalysed methane to methanol oxidation was conducted in an oleum medium at high temperature and pressure (215 °C and 40 bar). The catalyst showed a remarkable activity (albeit still lower than its homogeneous counterpart) and stability in such harsh conditions.

Kamiya *et al.* also employed platinum and a triazine framework to develop a methanol–tolerant oxygen reduction electrocatalyst.^171^ To improve the poor electrical conductivity of CTFs, carbon nanoparticles were introduced during its polymerisation process. Platinum from K_2_[PtCl_4_] was successfully coordinated to the resulted material. The catalyst showed a clear electrocatalytic activity for oxygen evolution in acidic media. Almost no activity for methanol oxidation was observed, in contrast to commercial carbon-supported platinum.

Rhodium complexes were also extensively employed to obtain porous heterogeneous catalysts. Fritsch *et al.* employed the aforementioned phosphorous-based framework EOF-17 to coordinate a Wilkinson catalyst to phosphorous-containing ligands.^135^ In 2012, Weston *et al.* reported the synthesis of a catechol-containing POP using a cobalt-catalysed acetylene trimerisation strategy.^172^ It was shown that post-metalation can be readily carried out with a wide range of metal precursors, such as Cu^II^, Mg^II^, and Mn^II^ salts and complexes. In 2014, together with Hock, the same catechol-containing POP was used to immobilise a Rh(i) complex (Fig. 7(b)).^170^ The coordination was confirmed by CP-NMR, EXAFS, and XANES. The obtained metalated POP was tested in vapour-phase plug-flow hydrogenation of propylene to propane. The catalyst showed a TOF of 22.5 h^–1^ at room temperature, while the oxidation state of rhodium remained unchanged. Rh(i) was proven to be the active catalytic site. When the catalyst was explored in toluene hydrogenation under the same conditions as propylene, it did not show any activity. A high temperature reduction of the Rh(i) metal centres to nanoparticles was performed; the obtained Rh(NP)(CAT-POP) converted toluene to methylcyclohexadiene (the ratio of H_2_ to toluene was approximately 1 : 1) quantitatively at 25 °C (TOF of 9.3 × 10^–3^ mol g^–1^ h^–1^).

Bavykina *et al.* immobilised an IrCp* (Cp* = pentamethylcyclopentadienyl) complex employing the bipyridine units of a CTF.^173^ The employed framework was made by the trimerisation of two building blocks – pyridine units introduced bipyridine moieties, while biphenyl units brought mesoporosity to the CTF. The successful coordination of Ir^III^ from [IrCl_2_Cp*]_2_ was confirmed by XPS. Chloride ions were removed by washing the solid in dimethylformamide (DMF). The catalyst was tested in hydrogen production from formic acid. The CTF worked not only as a support for the iridium complex, but also behaved like a non-innocent ligand – pyridine units were able to deprotonate formic acid, hence launching the catalytic cycle and avoiding the use of an external base. The catalyst exhibited the record activity for this reaction for a heterogeneous catalyst compared to nanoparticle-based and molecular heterogenised catalysts (initial TOFs of 27 000 h^–1^ were obtained). The catalyst also showed a remarkable stability – TONs of more than one million in continuous operation were obtained. The same group, in an attempt to bring the use of CTF-based molecular catalysts a step closer to industrial reality, reported a one-step approach for the production of porous, mechanically rigid, and easy-to-handle CTF-based spheres prepared by a phase inversion method using the polyimide Matrimid® as a binder.^174^ After obtaining the spheres, Ir^III^Cp* was coordinated to the bipyridine moieties of a CTF in a similar way as in previously mentioned works to obtain an efficient catalyst. Both powder and shaped catalyst were tried in the hydrogenation of carbon dioxide to formic acid. Spherically shaped composites showed a lower total activity than the powder, but any iridium loss related to handling, washing, or filtering the powder was fully eliminated. Yoon and co-workers employed the same approach for this reaction.^175^ A year later, the same group tested a heptazine-based organic framework instead. This catalyst showed a TON of 6400, the highest reported value for a heterogeneous system for carbon dioxide reduction to formic acid.^176^

A porous polymer catalyst for the same purpose of formic acid decomposition was reported by Hausoul *et al.*^177^ They employed a phosphorous-based polymer to coordinate the [RuCl_2_(*p*-cymene)] complex. The catalyst showed a high activity under base-free conditions, and recycling tests revealed a low level of leaching and only a minor yet gradual decrease in activity after seven catalytic runs. The catalyst was proposed to be applied in the facile removal of formic acid, which is a by-product of the conversion of cellulose to levulinic acid. Islam and co-workers described a facile *in situ* radical polymerisation of 2,4,6-triallyloxy-1,3,5-triazine in an aqueous medium in the presence of an anionic surfactant (sodium dodecyl sulphate) as a template.^178^ Ruthenium chloride was successfully coordinated to the obtained network; by XPS analysis it was shown that the oxidation state of ruthenium was II. The catalyst was tested in the Suzuki–Miyaura coupling of aryl halides and the transfer hydrogenation of carbonyl compounds. The catalyst showed a high activity and was recycled several times without appreciable loss of activity. The group of Xiao reported the preparation of a chiral catalyst (Ru/PCP-BINAP), a porous coordination polymer (PCP) obtained from the copolymerisation of divinylbenzene and chiral 2,2′-bis(diphenylphosphino)-1,1′-binaphthyl (BINAP) ligands.^179^ The obtained framework was coordinated with RuCl_2_(benzene); the coordination was confirmed by an obvious shift of UV-vis (ultraviolet-visible) spectra between PCP-BINAP and Ru/PCP-BINAP. To evaluate the catalyst efficiency, asymmetric hydrogenation of β-keto esters was performed. With a substrate/catalyst ratio of 2000, the highest reported enantioselectivity (for such ratio) was reported (94.6% ee). Even with the ratio increased to 5000, methyl-3-hydroxybutyrate was completely reacted with 90.1% ee. Such high enantioselectivity was a consequence of the incorporation of the BINAP ligands into the polymer backbone rather than grafting them into the framework. Also, the nature of the ruthenium coordination to BINAP is quite similar to the homogeneous catalyst.

This year, Rozhko *et al.* reported the utilisation of different POFs (covalent triazine and imine-linked frameworks) bearing free nitrogen atoms as supports for a nickel-based ethylene oligomerisation catalyst.^180^ These new catalysts displayed an activity comparable to that of their homogeneous counterparts and up to a fivefold higher selectivity to C_6_^+^ olefins, depending on the textural properties of the support. Accumulation of long chain hydrocarbons within the porosity of the COFs leads to reversible deactivation, but the full activity and selectivity of the best catalysts could be recovered upon washing with 1,2-dichlorobenzene.

Zhang *et al.* synthesised a microporous polyisocyanurate (PICU) *via* the cyclotrimerisation of diisocyanate using N-heterocyclic carbine as a catalyst.^181^ Fe/PICU was prepared by suspending PICU in a hot solution of FeCl_2_ in DMF and was tested for the oxidation of benzyl alcohol with hydrogen peroxide. Shultz *et al.* synthesised a POP containing a free-base porphyrin subunit by the condensation of bis(pttalic acid)porphyrin with tetra(4-aminophenyl)methane (Fb-PPOP).^182^ Post-metalation was performed using FeCl_2_ or MnCl_2_·4H_2_O, achieving Fe- and Mn-PPOP respectively. Epoxidation of styrene was examined, where both catalysts showed better stability than the homogeneous porphyrin analogues. Saha *et al.* also employed a porphyrin-based framework to support iron.^183^ In this case, though, Fe^III^-POP-1 was obtained *via* a one-pot synthesis by reacting pyrrole with terephthaldehyde in the presence of FeCl_3_. Electron paramagnetic resonance (EPR) analysis confirmed that iron was in the oxidation state III after the coordination and remained in this oxidation state after several catalytic runs. Fe^III^-POP-1 was tested in the aerobic oxidation of 5-hydroxymethylfurfural (HMF) to 2,5-furandicarboxylic acid (FDCA). The catalyst showed a high activity and its heterogeneity was proven by hot filtration tests. Kraft *et al.* coordinated iron to a catecholate-containing porous organic polymer, in a fashion similar to the previously described rhodium coordination.^170^ Fe[N(SiMe_3_)_3_]_2_ was chosen as the iron source to obtain the Et_2_OFe(CAT-POP) catalyst. It was tried in the hydrosilylation reaction of aldehydes and ketones with phenylsilane. The catalyst is fully reusable, recyclable for three catalytic cycles, and shows high thermal stability.^184^ In a separate work by the same group, Et_2_OFe(CAT-POP) was extensively characterised by *in situ* X-ray absorption spectroscopy (XAS) under a variety of conditions and used as a catalyst in the hydrogenation of different olefins.^185^

The use of carbon nanoparticles/CTF (CTF/CPs) composites discussed above was further extended to obtain non-noble metal electrocatalysts for oxygen reduction reactions (ORRs).^171^ The copper version of this system was prepared by the coordination of CTF/CPs with CuCl_2_.^186^ The resulting Cu-CTF/CPs was reported to be a very efficient electrocatalyst for the ORR in neutral solutions. The same catalyst was also found to be efficient in the electrochemical reduction of nitrate to nitrous oxide.^187^

Iglesias and co-workers described the synthesis of two imine-linked POFs with different geometries.^188^ C3v-POF and Th-POF were obtained by combining 1,4-benzenedicarbaldehyde with 1,3,5-tris(4-aminophenyl)benzene and tetra-(4-aminophenyl)methane, respectively. Th-POF exhibited a higher BET area and a higher metal uptake after post-synthetic metalation of the framework than C3v-POF. Therefore, only Th-POF was employed as a catalyst support. When used as a catalyst in the cyclopropanation of alkenes, the Cu(i)-based catalysts showed good conversion and diastereoselectivity (51% and 79% respectively, 5–10 wt% of Cu). The Ir-Th-POF compound was explored for the hydrogenation of alkenes. In the case of hydrogenation of 1-octene, a conversion of 100% was obtained with TOFs of 5880 h^–1^ (Ir loading is 0.1 mol%).

Puthiaraj *et al.* described the synthesis of a mesoporous covalent imine polymer (MCIP-1) *via* the Schiff-base condensation of 2,4,6-tris(*p*-formylphenoxy)-1,3,5-triazine and mesitylene.^189^ Post-metalation was performed by stirring the polymer with copper acetate in CH_2_Cl_2_. The obtained catalyst, Cu/MCIP-1, was used in the Chan–Lam cross-coupling *N*-arylation under mild conditions. Roy *et al.* anchored Cu^II^ to a nitrogen-rich imine network to obtain the Cu^II^-CIN-1 (CIN = nitrogen-rich porous covalent imine) catalyst for the synthesis of asymmetrical organoselenides from aryl boronic acids.^190^ The coordination of the copper species was confirmed by EPR, XPS, and UV-vis DRS (diffuse reflection spectroscopy) analyses.

The group of Nguyen has extensively studied metal catalysts supported on catecholate-based frameworks. In this review, rhodium- and iron-containing catalysts were already discussed, while this approach was extended to other metals.^170^,^182^ As a result, lanthanum was successfully coordinated to the catecholate-functionalised POF.^193^ The catalyst was employed in the solvolytic and hydrolytic degradation of the toxic organophosphate compound methyl paraoxon, a simulant for nerve agents. Ta^V^ trialkyl was stabilised in the same framework and tested for the hydrogenation of cyclohexene, showing an enhanced activity compared to its homogeneous analogue.^194^ In a separate work, five different species – V^III^, Cr^III^, Mn^II^, Co^II^ and Ni^II^ – were incorporated into the catecholate-based framework.^195^ A similar approach to bind a metal *via* its coordination to hydroxyl groups was reported by the Lin group.^196^ Five chiral cross-linked polymers (CCPs) based on 1,1′-binaphthyl were prepared *via* the trimerisation of terminal alkyne groups by the Co_2_(CO)_8_ catalyst. The CCPs were treated with Ti(O^i^Pr)_4_ to generate chiral Lewis acid catalysts for the asymmetric addition of diethylzinc to aldehydes. The catalysts were reused ten times without any loss of conversion or enantioselectivity (from 55% to 81% ee for different frameworks). An *et al.* synthesised an α,α,α′,α′-tetraaryl-1,3-dioxolane-4,5-dimethanol-based (TADDOL) chiral porous polymer, TADDOL-CPP.^197^ Using Ti(O^i^Pr)_4_, TADDOL-CPP/Ti was also tested in the asymmetric addition of diethylzinc to aldehydes, and presented an excellent enantioselective control to a variety of aldehydes.

**Fig. 8:**
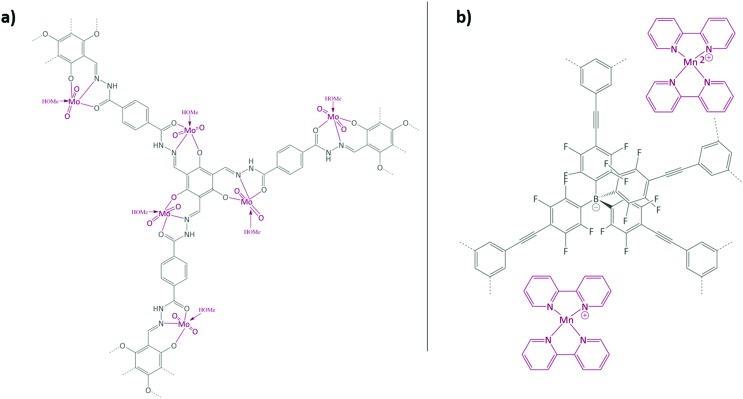
(a) Molybdenum supported on a POF catalyst; (b) manganese supported on an ionic framework catalyst. Figures adapted from ref. 191 and 192.

Aiyappa *et al.* developed a Co-TpBpy catalyst for water electro-oxidation. A bipyridine-containing framework was used as a support for Co^II^ catalysts. The obtained catalysts exhibited an exceptional stability: even after 1000 cycles and 24 h of oxygen evolution reaction activity in a phosphate buffer under neutral pH conditions with an overpotential of 400 mV at a current density of 1 mA cm^–2^, the material retained 94% of its activity with a TOF of 0.23 s^–1^ and a faradaic efficiency of 95%.^198^ Mackintosch *et al.* developed phthalocyanine- and porphyrin-based PIMs. Cobalt was incorporated into the phthalocyanine framework and the obtained solid was tested in H_2_O_2_ decomposition, cyclohexene oxidation, and hydroquinone oxidation.^199^ Similarly, iron was introduced into this porphyrin-based PIM. The iron catalyst showed a superior activity for hydroquinone oxidation. Zhang *et al.* synthesised a molybdenum-doped framework linked by a hydrazine linkage.^191^ Molybdenum species were introduced into the framework from a MoO_2_(acac)_2_ source to obtain a catalyst (Fig. 8(a)) for the epoxidation of different alkenes.

Thomas and co-workers reported the synthesis of an anionic microporous polymer network, prepared by using lithium tetrakis(4-bromo-2,3,5,6-tetrafluorophenyl)borate as a tecton *via* Sonogashira coupling.^192^ The Li^+^ cations were exchanged for Mn^2+^ cations, which were further coordinated with bipyridine to obtain a catalyst for the oxidation of styrene (Fig. 8(b)). The solid is recyclable and stable at least during three runs, and hot filtration tests confirmed the heterogeneity of the catalyst.

#### C.3.2. Top-down metal-free POF-based catalysts

Modak *et al.* designed a cross-linked organic polymer, COP-M, from 2,4,6-tris(bromomethyl)mesitylene and 4,4′-bis(bromomethyl)-1,1′-biphenyl *via* Friedel–Crafts alkylation.^200^ COP-A, bearing acidic –COOH groups, was obtained from alkaline KMnO_4_ oxidation of methyl-functionalised COP-M. COP-A showed an unprecedented catalytic activity in indole C–H activation at room temperature.

Xu *et al.* constructed a mesoporous imine-linked porphyrin COF as a scaffold in which the porphyrin units are located at the vertices and the phenyl groups occupy the edges of tetragonal polygon frameworks.^201^ The COF catalyst showed a significantly higher catalytic activity in a Michael addition reaction than the monomeric catalyst, while retaining the stereoselectivity.

Gascon and co-workers reported the synthesis, characterisation, sulphonation, and catalytic performance of two new PAFs obtained by the Suzuki–Miyaura cross-coupling of the commercially available precursors 1,3,5-tris(4-bromophenyl)benzene or tris(4-bromophenyl)-amine and benzene-1,4-diboronic acid.^131^ Post-synthetic treatment in sulphuric acid led to the sulphonation of approximately 65% of the benzene rings in the polymers. The sulphonated materials displayed an excellent catalytic performance in the acid-catalysed esterification of *n*-butanol and acetic acid. The catalysts have a similar or even superior performance over multiple catalytic cycles to that of the state-of-the-art catalyst Amberlyst-15. The obtained TOFs for the first reaction run were 1.06 min^–1^ in the case of the porous polymer, while the test with Amberlyst-15 resulted in a TOF of only 0.7 min^–1^. The higher activity of the porous polymer was explained by its higher sulphonic acid content.

## D. Modelling heterogeneous single-site catalysts

Molecular modelling of heterogeneous catalysis is a field of its own. Theoretical studies may assist in understanding the function of the active sites, opening perspectives to design heterogeneous catalysts. However, a plethora of techniques is available and many decisions need to be made, which may have a drastic influence on the outcome of the modelling procedure. Hereafter, we will give an overview of how modelling can assist in the characterisation and understanding of the function of heterogeneous single-site catalysts. Computational modelling of active sites yields molecular insight in complex chemical transformations, which are sometimes difficult to track at the molecular level from an experimental point of view.

This section is organised as follows: first of all, a brief overview of general modelling concepts necessary for modelling heterogeneous single-site catalysts is given. In the second part, we illustrate by means of selected case studies how such modelling strategies may be used to obtain molecular insight into the reactivity of a diverse set of active sites within the framework.

### D.1. General modelling principles

**Fig. 9:**
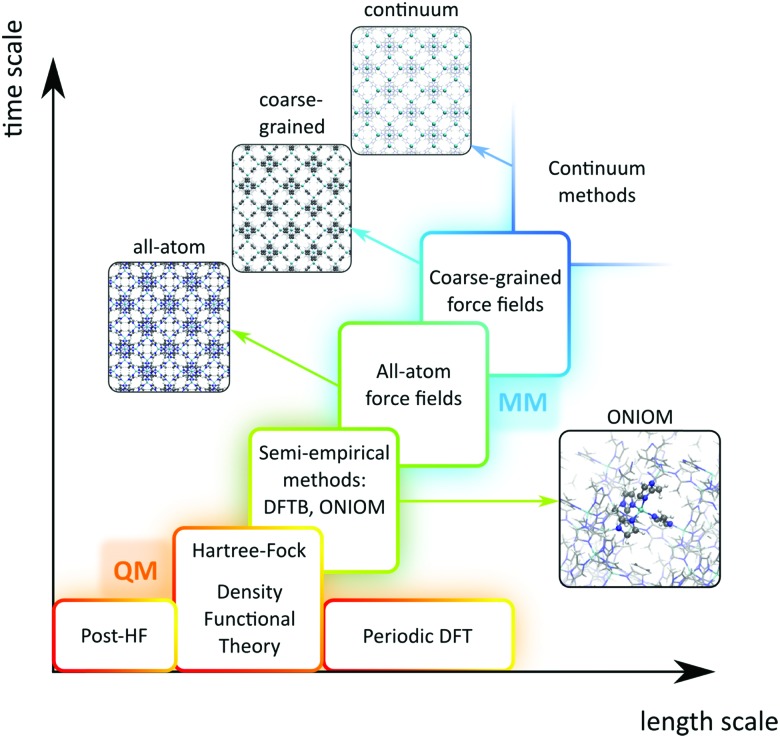
Schematic overview of length and time scales of methods going from purely quantum mechanical (QM) based methods to force field (MM) and coarse-grained methods.

A plethora of modelling techniques is available, which are often categorised into various length and time scales, as schematically shown in Fig. 9. For large systems or long simulations, force-field based methods, which efficiently describe the internuclear interactions based on classical potentials, are ubiquitously used. Within the field of MOFs, various protocols have been set up to derive force fields from first principles such as MOF-FF,^202^ BTW-FF,^203^ QuickFF,^204^,^205^ among others.^206^,^207^ However, since force fields require a predefined connectivity between the atoms, these molecular mechanics (MM) techniques cannot model the reactivity of heterogeneous single-site catalysts. Rather, the electronic structure needs to be described from first principles to account for the formation and breaking of bonds, using quantum mechanical (QM) methods. Hence, all modelling approaches in this review are based on schemes in which at least the reactive part of the system is described using QM methods. Also hybrid methods exist which inherit the advantages of both QM and MM methods. In these QM/MM methods, part of the system, typically the subsystem that participates actively in the chemical reaction, is modelled quantum mechanically, whereas the rest is treated at a lower level of theory with for example a force field. An example of such approach may be found in the work of Yadnum *et al.* where the Mukaiyama aldol reaction is studied using an ONIOM-based (Our own N-layered Integrated Molecular Orbital and Molecular Mechanics) approach.^208^,^209^ The layered approach has been extended for some systems by using two quantum mechanical based methods. In such case, the inner part is treated using a high-level electronic structure method, while the outer part is treated at a lower, computationally more attractive level of theory.

For catalytic processes in framework materials, one needs to choose an appropriate structural model to represent the extended periodic environment of the material. Various methods accounting for the topology of the material are discussed in the next part of this section. Afterwards, the influence of choosing different electronic structure methods to model adsorption and reactivity in nanoporous materials will be introduced. We conclude by discussing how free energy profiles at the true reaction conditions can be obtained.

#### D.1.1. Modelling the topology of the framework

**Fig. 10:**
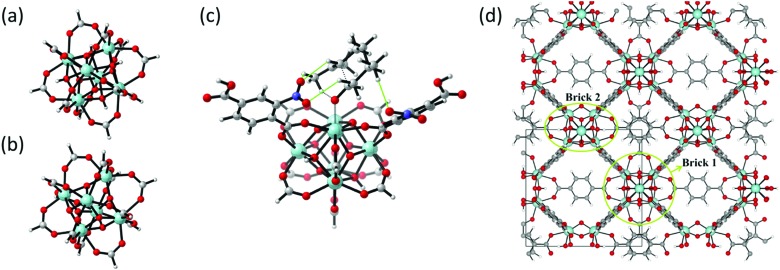
(a) and (b) The formate-terminated inorganic zirconium nodes of UiO-66, in their hydrated Zr_6_O_4_(OH)_4_(OOCH)_12_, (a) and dehydrated Zr_6_O_6_(OOCH)_12_, (b) form; (c) cluster model of the transition state of the cyclisation from citronellal to isopulegol with two explicit nitro-functionalised BDC linkers; (d) periodic model of UiO-66 comprised of fully coordinated inorganic nodes (brick 1) and defect-containing inorganic nodes (brick 2), with indication of the periodic unit cell. Panels (c) and (d) reproduced from ref. 76 and 216 with permission of Wiley and Elsevier, copyright 2012 and 2015.

The topology of the material can either be modelled using an extended cluster model in which a representative part of the material is considered or using a periodic model where the full unit cell of the material is taken into account using periodic boundary conditions. Fig. 10 illustrates a UiO-66 active site within these two models.

Earlier computational studies on catalysis in MOFs used cluster models to represent the catalytically active sites, as they are computationally very efficient. These numerical algorithms, implemented in programs such as Gaussian,^210^ Turbomole,^211^ or Jaguar,^212^ are typically better suited to localize transition states than those implemented in periodic structure codes. Another advantage of the small cluster approach is the ability to use very accurate electronic structure methods, which is inherently linked to the small number of atoms contained in these clusters.^213^–^215^ While these calculations may be used for benchmark purposes, they are inadequate for reactions involving larger species, as the molecular environment is completely neglected. Moreover, the selection of the cluster and its termination may affect the results substantially. Furthermore, by cutting molecular clusters out of the periodic system, one often creates highly charged clusters which need to be compensated by cations or anions to reach charge neutrality.^214^ Systematic studies on the impact of the size of the cluster model are not yet readily available within the fields of MOFs and COFs, in sharp contrast to the field of zeolites. Based on some selected examples, however, we will illustrate the pros and cons of cluster models for modelling catalytic reactions within MOFs.

As discussed above, one of the materials that received considerable attention within the field of MOF catalysis is UiO-66 (see Fig. 3), possessing a high thermal and chemical stability and a good resistance toward water and several alcohols.^217^,^218^ This material is a showcase example where modelling and experimental efforts give a complementary understanding on the nature of the active sites. In one of the earlier studies on the citronellal cyclisation in UiO-66, some of the present authors initially built small cluster models to unravel the nature of the active site. In this case, all 1,4-benzenedicarboxylate (BDC) linkers were replaced by formate ligands (HCOO^–^), yielding the Zr_6_O_4_(OH)_4_(HCOO)_12_ and Zr_6_O_6_(HCOO)_12_ models of Fig. 10(a) and (b) as representations of the hydrated and dehydrated nodes of the material. These early calculations were very instructive as they immediately indicated the need for introducing OMSs to obtain catalytically active sites. Indeed, the combined theoretical and experimental efforts showed that structurally missing linkers were necessary to activate the material towards catalysis. The electronic modulation effects observed by Vermoortele *et al.* were rationalised by theoretical calculations of the rate constants on extended cluster models bearing two functionalised BDC linkers, as indicated in Fig. 10(c).^76^ It was observed that nitro groups increased the conversion as they allowed for a stronger adsorption on the Lewis acid sites but also provided additional stabilisation effects in the reactant and transition states due to specific interactions with the linkers and their substituents.

**Fig. 11:**
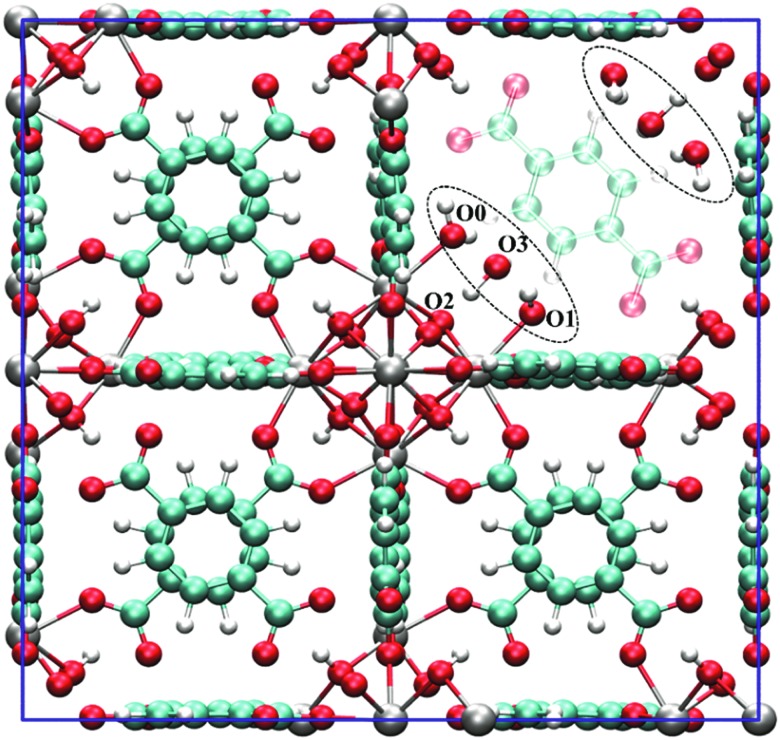
The unit cell of hydrated UiO-66 with one missing BDC linker. In the most stable configuration, the defect site is surrounded by three water molecules. Reprinted from ref. 225 with permission of the Royal Society of Chemistry, copyright 2016.

Despite these early successes of cluster-based calculations, our improved understanding of the catalytic active site forced us to go beyond the cluster model approach. Whereas initially catalysis on undercoordinated active sites focused primarily on the Lewis acidity, more evidence was presented recently that cooperative effects between the Lewis acid site and neighbouring Brønsted base sites might occur.^216^,^219^ Moreover, the catalytic activity of the material may be modulated by the presence of other species in the pores of the material, such as water. Such complexity necessitates to go beyond the cluster-based approximation. For UiO-66-type materials, an intensive debate on the chemical nature of missing linkers can be found in literature. Upon removal of a charged BDC linker, various charge-balancing species such as formate, chlorate, and hydroxide have been suggested to terminate the inorganic nodes at linker vacancies.^50^,^220^–^224^ Recent static and dynamic first principles studies, accounting for the full periodic environment of the material, revealed a dynamic and labile acid centre that may even be tuned for catalytic applications (Fig. 11).^225^ In this most stable defect configuration, one undercoordinated zirconium atom is coordinated to a neutral water molecule, whereas the other undercoordinated zirconium atom is coordinated with a hydroxide anion. The latter is further stabilised by interaction with the μ_3_-OH group present in the inorganic node. Ling and Slater also performed first principles molecular dynamics simulations at several temperatures to investigate the proton mobility of the various defect structures, thus accounting for the dynamic state of the water environment. At higher temperatures, some of the physisorbed water molecules diffused away from the zirconium sites into the pores of the material. This clearly shows that simple cluster-based calculations are insufficient to study this dynamic behaviour, and more advanced models are necessary to account for the nature of the active site at operating conditions. The use of such dynamic methods to study the reactivity itself has not been used so far within the field of MOFs, but would open very interesting perspectives for future modelling studies (*vide infra*).

Periodic calculations are becoming more and more common within the field of MOFs. Unlike cluster models, periodic models consider either the full unit cell or a supercell of the material consisting of several repetitions of the unit cell. This is the most natural approach to represent the framework material, but comes at a serious computational cost. Periodic models have the advantage that all factors depending on the structure of the nanoporous material – *e.g.* the location of the active sites in the framework, the shape of the pores and channels, and the flexibility of the porous material – are taken into account in a more natural way. We refer to the review of Odoh *et al.* for a more extensive discussion on the choice of basis sets, pseudopotentials, and other technicalities.^226^ The codes used for these calculations, *e.g.* VASP,^227^–^229^ CRYSTAL,^230^ FHI-aims,^231^ and CP2K,^232^ typically originate from the solid-state community and one needs to be careful when transferring the procedures applicable for rigid solids to (flexible) nanoporous materials. For the latter, structures are preferably optimised by constructing an equation of state to locate the optimal volume.^233^ Following such a procedure, Hajek *et al.* studied the aldol condensation in UiO-66-type MOFs both by periodic and extended cluster calculations.^216^ While the reaction mechanism remained qualitatively the same in both approaches, the reactants were more strongly adsorbed by about 20 kJ mol^–1^ when properly accounting for the confinement effects using the periodic model.

**Fig. 12:**
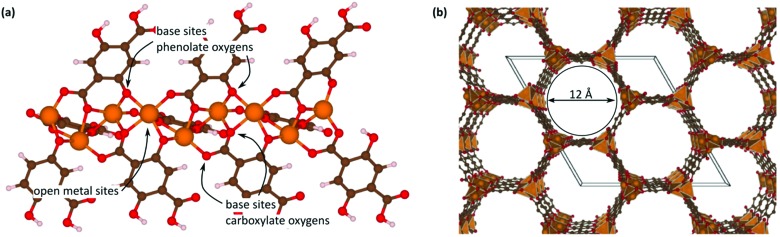
(a) Representation of the optimised cluster model of MOF-74 with indication of the potential base sites and open metal sites in the framework; (b) periodic structure of MOF-74 with indication of the 1.2 nm pores. Figure adapted from ref. 219 with permission of Elsevier, copyright 2014.

Some properties, such as proton affinities or deprotonation energies of periodic materials, may not be well represented by a periodic model due to the charge induced in the unit cell when adding a proton to or removing a proton from the framework. In these cases, cluster models may be very valuable to give complementary insight. For instance, the active sites of MOF-74 were explored for classical base-catalysed reactions such as Knoevenagel condensations and Michael additions.^219^ In Fig. 12(a), the potential active sites in this material are shown. The structure consists of metal oxide chains connected by 2,5-dioxidoterephthalate (DOBDC) linkers and possesses both coordinatively unsaturated sites and possible base sites. While the carboxylate oxygen atoms are only weakly basic, the phenolate oxygens are the conjugate bases to the weakly acidic phenol and are hence expected to be much more basic. This was confirmed by theoretically calculating proton affinities of both the phenolate and carboxylate oxygens for different metals, using cluster models cut from this periodic structure.

#### D.1.2. Choice of appropriate electronic structure methods for adsorption and reactivity

**Fig. 13:**
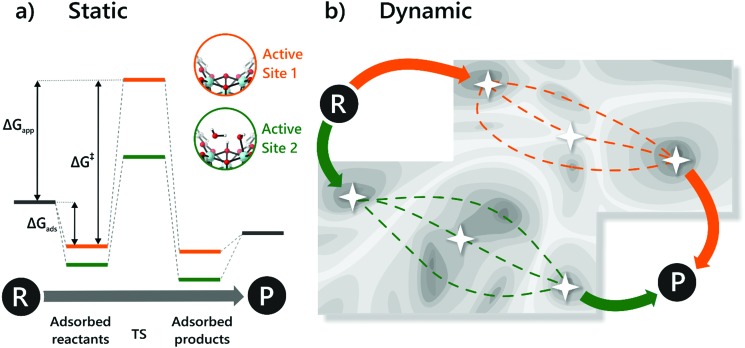
(a) 1D free energy profiles for a given reaction on two different active sites in UiO-66 (insets), indicating the adsorbed initial and final states and the localised transition state for this reaction. The adsorption free energy, intrinsic and apparent barriers are obtained by static calculations and indicated by Δ*G*_ads_, Δ*G*^‡^, and Δ*G*_app_, respectively; (b) possible 2D representation of the given reaction on the two active sites as obtained using advanced dynamic techniques, indicating the three critical points on the potential energy surface.

Within this review, the attention goes to catalytic processes taking place at a single active site. A schematic illustration of the reaction profile for such a heterogeneously catalysed reaction is shown in Fig. 13(a). This profile is typically composed of adsorption steps and activation steps, and is obtained by determining the electronic energy at critical points along the reaction profile, *e.g.*, the reactants, intermediates, products, and transition states. While the electronic energy is only one of the ingredients to determine the final thermodynamic quantities, it is crucial since it determines to a great extent the accuracy of the final free energy profile at the true reaction conditions (see Section D.1.3). It is also by far the computationally most expensive part of the calculation.

Any heterogeneously catalysed reaction starts with the adsorption of the various reactants. A thorough understanding of this adsorption step is hence crucial to understand the kinetics of catalytic processes. There are various methods available to describe the adsorption of guest species within the pores of a nanoporous material, and MOFs have attracted a lot of attention in this field.^234^,^235^ To describe the thermodynamics of adsorption in nanoporous materials, grand canonical Monte Carlo (GCMC) simulations are ubiquitously used. They are typically performed with classical force fields that do not explicitly take into account the electronic structure of the host–guest interactions. Thus, the quality of the results depends on the quality of the classical potentials describing the host–guest interactions. Particularly for the interactions with the OMS this might be problematic and hybrid approaches that also incorporate some QM information may provide a substantial improvement of the adsorption mechanism.^236^ From these GCMC simulations, adsorption isotherms and insight into the most probable adsorption sites can be obtained, which may then be investigated more deeply with advanced electronic structure methods.^237^

In general, a theoretical description of adsorption in nanoporous materials is very challenging since the accuracy depends on the treatment of the noncovalent interactions, which include both electrostatic and dispersion interactions. Dispersion interactions, which result from many-particle electron correlation effects, result in long-range attractive forces that may act between separated molecules even when no permanent multipole moments are present. Only very advanced first-principles methods based on correlated wave functions may capture these effects, but are restrictively expensive from a computational point of view and hence not routinely applicable to the extended systems at hand.^238^–^240^ To describe an overall catalytic cycle from first principles, it is necessary to calculate the adsorption steps and reactive events consistently with the same quantum mechanical based method. Thus, all levels depicted in Fig. 13(a) have to be described at the same level of theory to obtain consistent free energies. A large variety of electronic structure methods are available to determine the energy of the system for a given atomic configuration. In the review of Odoh *et al.*, many of these theoretical methods are introduced with illustrations of their applicability to calculate a variety of properties such as ground-state structural properties, spectroscopic signals, and band gaps, among others.^226^

The method of choice to describe the electronic properties of the system is based on Density Functional Theory (DFT), which is computationally very attractive even for large systems. Within the MOF field, only a very limited number of studies are available that go beyond DFT, using computationally more expensive post-Hartree Fock methods based on configuration interaction (CI) or coupled clusters (CC).^241^–^243^ However, the accuracy of DFT depends on the choice of the exchange–correlation functional and most of the commonly applied local functionals fail to accurately describe the long-range dispersion interactions. Various pragmatic solutions have been suggested to remedy this deficiency in modern DFT approaches, such as the addition of a parametrised damped dispersion term to standard functionals such as the Perdew–Burke–Ernzerhof (PBE) or the Becke-three-parameter-Lee–Yang–Parr (B3LYP) functionals.^244^–^246^ Tkatchenko and Scheffler introduced a parameter-free method to derive the interatomic coefficients entering the dispersion term.^247^,^248^ This deficiency in describing long-range dispersion interactions can also be remedied by constructing a non-local van der Waals functional that accounts for the long-range electronic correlations.^249^–^251^ Grajciar *et al.* proposed a combined DFT/CC method that does not simply include a parametrised functional term to add the missing dispersion term, but attempts to correct the DFT error in a systematic way.^252^

**Fig. 14:**
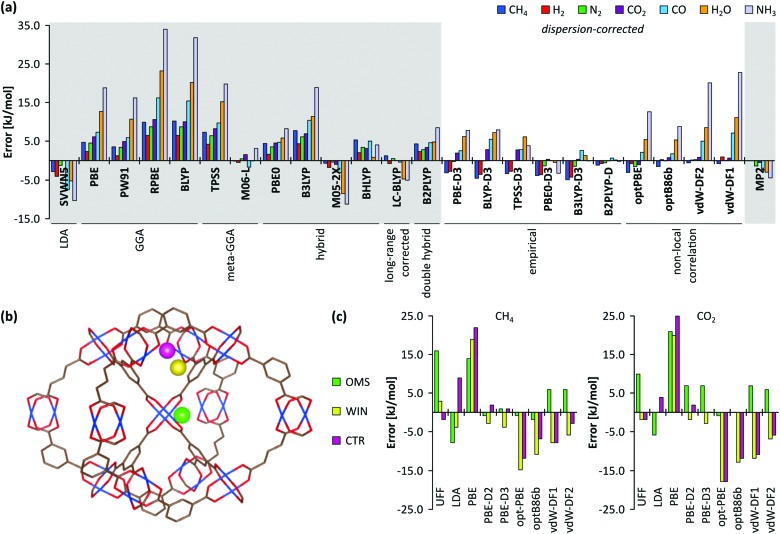
(a) Errors in the interaction energies of adsorbate-Cu(HCOO)_2_ calculated with respect to CCSD(T)/CBS level of theory; (b) the CuBTC supercage with indication of an open metal site (OMS, green), a tetrahedral cage-window site (WIN, yellow) and a tetrahedral cage-centre site (CTR, magenta) as the three main adsorption sites; (c) errors in the interaction energies calculated for CH_4_ and CO_2_ at the three main adsorption sites with respect to the DFT/CC reference level of theory. Figure adapted from ref. 253 with permission from the American Chemical Society, copyright 2015.

To illustrate the level of accuracy one can obtain with currently available adsorption methods, it is interesting to discuss some of the results obtained by another study of Grajciar *et al.*^253^ They performed a comprehensive study on the adsorption of a series of small molecules (CH_4_, H_2_, N_2_, CO_2_, CO, H_2_O, NH_3_) on HKUST-1 using an extensive set of DFT-based methods, including both cluster and periodic approaches, and applying different dispersion models. Furthermore, calculations were performed on Cu^2+^ and Fe^3+^ containing OMSs to investigate the influence of the metal. Coupled-cluster calculations obtained within a cluster model and extrapolated to the complete basis limit were used as reference data. By considering various guest molecules, the study reveals interesting aspects on the different types of adsorbent–adsorbate interactions such as dispersion, electrostatic, and partially covalent bonding. The investigation indicates that there is no universal method that suits all systems and underlines the difficulty in describing the adsorption of small molecules at OMSs from a theoretical point of view. Some of the results are shown in Fig. 14. When using cluster models with functionals that do not explicitly add a dispersion correction, the interaction energies are generally strongly underestimated with respect to the high-level benchmark values. Some functionals, such as M06-L (the local Minnesota '06 functional), perform better since they have been parametrised towards a dataset that includes some of the nonlocal correlation effects. Adding dispersion interactions by including an empirical correction term significantly improves the results as can be seen from Fig. 14. In HKUST-1, it is possible to distinguish between three major adsorption sites. The adsorption at the OMS is dominated by electrostatic interactions with formation of a partial dative bond, whereas adsorption at the cage-centre (CTR) and cage-window (WIN) sites is governed by the interaction with the organic linkers and dominated by dispersion interactions. The results obtained for the adsorption of CH_4_ and CO_2_ on each of these sites using periodic models are shown in Fig. 14(c). The methods that do not include explicit dispersion corrections have prohibitively large errors. For the PBE functional, the inclusion of Grimme dispersion corrections (D2 or D3) improves the results significantly. However, an accurate description of CO_2_ adsorption at the OMS remains very challenging.

**Fig. 15:**
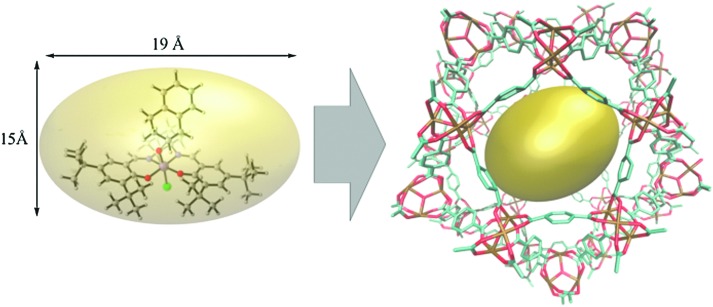
Schematic representation of the transition state contained in the small MIL-101 cage. Hydrogens and amine groups are omitted for clarity, the inner ONIOM layer is represented by the ellipsoid. Reprinted from ref. 254 with permission of the Royal Society of Chemistry, copyright 2013.

Also for modelling transition states it might be very challenging to select a proper electronic structure method. Pragmatic solutions have been proposed by comparing DFT results with high-level theoretical methods. This procedure was followed for the theoretical description of the manganese–salen complex that is used for the enantioselective epoxidation of nonfunctionalised olefins. Recently, this complex was entrapped in MIL-101 showing the same selectivity as the homogeneous analogue.^254^ The selectivity was unravelled by DFT calculations, yet required the use of a proper DFT functional to yield the correct ordering of the spin states.^255^ OPBE was selected from a broad range of exchange–correlation functionals, as it gave the right ordering of the spin states compared to benchmark DMRG (density matrix renormalization group) calculations. Finally, ONIOM calculations were performed to assess the influence of the confinement in the cage. Here, the manganese complex was modelled using the selected DFT method and the rest of the cage with a universal force field as schematically shown in Fig. 15. The latter case study shows how an ingenious combination of various modelling tools may assist in unravelling the reaction profile.

#### D.1.3. Free energy profiles at reaction conditions

Once the electronic energy is determined at the critical points along the reaction profile, thermodynamic quantities, such as enthalpy, entropy, and free energy, must be evaluated to allow for a comparison with experimental data. This comparison is based on the principles of statistical physics and more precisely the determination of the molecular partition function.^256^ Within a static approach, *i.e.* when assessing the reactivity based on a limited number of points on the free energy surface, thermal corrections and entropy contributions are generally determined using a harmonic oscillator approximation, in which all anharmonic motions such as internal rotations or translations of adsorbates relative to the framework are neglected. However, soft, low-frequency modes are typically present for molecule–surface interactions, which are very hard to determine accurately and which moreover have a substantial impact on the final entropic contributions.^257^,^258^ In this low-frequency region, anharmonic corrections should be used. Some models have been proposed in the literature, but so far they have not been used for reactions taking place in MOFs.^257^,^259^ Within other fields, such as zeolite catalysis, more progress has been made on the methodological side and it has been shown that accurate enthalpy barriers and rate constants can be predicted with chemical accuracy for reactions taking place at a single active site.^258^–^261^ At true operating conditions, the scene at the nanoscale level is often far more complex, and it might become necessary to determine macroscopic thermodynamic and kinetic quantities from more advanced techniques (see Section H).

### D.2. Modelling the reactivity on single active sites: selected case studies

Herein, we illustrate various modelling principles for a selected number of case studies, based on the division introduced in Fig. 1. For **type I** heterogeneous catalysts, catalysis is achieved on the structurally embedded metal nodes that form secondary building units in the framework. As discussed in Section B, while some MOFs comprised of fully coordinated inorganic nodes lack active sites at first sight, several strategies were implemented to intentionally create active sites by introducing defects.^106^**Type I** catalysts are hence characterised by open metal sites and encompass active sites created by structural defects, active sites due to catalytically active terminating ligands, and active sites due to the framework topology prohibiting the complete saturation of the coordination sphere of the metals in the inorganic node. In **type II** catalysts, metalloporphyrins form the catalytically active sites. In these metalloligands, the catalysis takes place at the metal atom embedded in the porphyrin ligand. The nature of the catalytic site may be tuned by post-synthetically altering the metal atom at the centre of the porphyrin ligand. **Type III** actives sites are based on covalently anchored functional groups on the organic linker, which are catalytically active. These functional groups, already present during synthesis or added post-synthetically, are terminating groups that do not connect different building blocks of the framework material, differentiating them from the metalloporphyrins. The inclusion of organic and inorganic functional groups onto the framework, the latter often termed metalation, will be discussed separately due to its different catalytic nature.

The classification used here is not directly applicable to POFs, since they do not contain inorganic nodes. Hence, only the discussion on **type II** or **type III** active sites may also apply to POFs. Indeed, POFs may be active for catalysis by incorporating metalloporphyrins or by anchoring active complexes to the organic building units by means of postfunctionalisation. Modelling studies on POFs are making their entrance into the field, but are still more restricted than the available literature for MOFs and, for catalytic purposes, are focused on the band gap dispersion. Due to the absence of heavy atoms, POFs are promising photocatalysts, and modelling studies hence concentrate on unveiling the electronic and optical properties of these materials. For 2D COFs, a density functional based tight binding (DFTB) computational study by Lukose *et al.* indicated that the stacking of the monolayers in these materials does not affect their electronic structure significantly.^262^ However, the concentration of nitrogen atoms was shown to influence the optical and electronic properties for several test systems.^263^–^265^ For 3D COFs, Yang *et al.* consistently investigated boron-based COFs sharing the MOF-5 topology using the PBE functional within the DFT paradigm. They showed that the mechanical, optical, and electronic properties of these COFs can be tuned systematically by varying the atoms contained in one of the secondary building blocks.^266^ However, Meunier and co-workers revealed by comparing GW calculations with regular DFT calculations that many-body effects lead to a non-negligible increase in the band gap.^267^,^268^ Hence, in this early stage of theoretical research in COFs as catalysts, benchmark studies comparing the accuracy of different levels of theory are a prerequisite.

#### D.2.1. Type I: catalysis at open metal sites (OMSs)

**Fig. 16:**
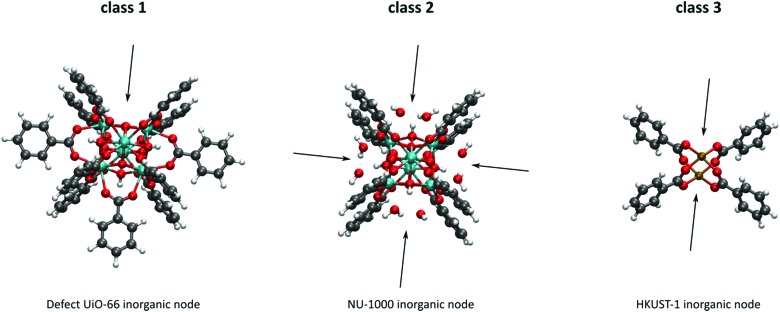
Schematic representation of the three types of single sites available for catalysis at the inorganic node: UiO-66 (class 1), NU-1000 (class 2) and HKUST-1 (class 3).

Several OMS classes are illustrated in Fig. 16 for the UiO-66, the NU-1000, and the HKUST-1 frameworks and are thoroughly discussed below. A molecular understanding of OMSs present at the inorganic nodes of the catalyst is quintessential to develop predictable heterogeneous catalysts engineered for particular applications. This computational modelling, however, does not only involve the study of the actual catalytic reaction. It can also be applied to study the sequence of steps leading to the formation of catalytically active sites in the framework material, starting from investigating the influence of synthetic and post-synthetic conditions on the formation of the catalytic site, to modelling the adsorption of the reactive species on the active site, as indicated below.

##### Class 1: OMSs due to structural defects

**Fig. 17:**
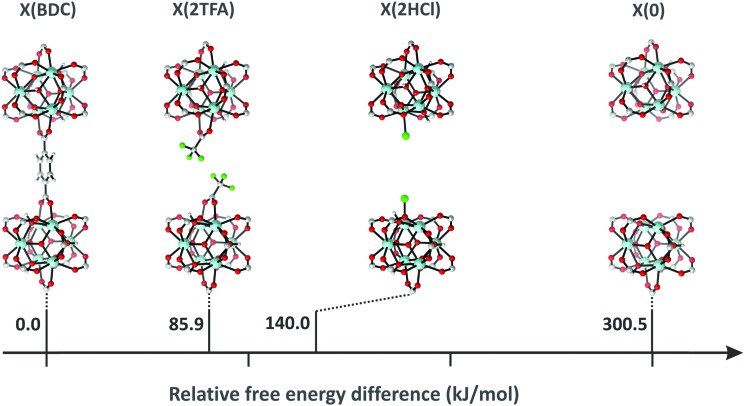
Schematic representation of the calculated structures with different capping species. Adapted from ref. 90 with permission of the Royal Society of Chemistry, copyright 2015.

Arguably the best-known example of this class is UiO-66.^50^,^89^ In an extensive study by Shearer *et al.*, the effect of the type and amount of modulator added during synthesis was observed to determine the degree to which structural defects are present.^269^ From a theoretical point of view, the effect of modulators was investigated by Vandichel *et al.* in an attempt to obtain more insight into the formation of different active sites.^90^ In this study, periodic DFT-D geometry optimisations were carried out in VASP, using the PBE exchange–correlation functional with Grimme D3 dispersion corrections.^270^ One BDC ligand was removed, and charge neutrality was obtained by capping the OMSs with trifluoroacetate, chloride, and/or hydroxide, or by deprotonating one of the hydroxo-groups present in the inorganic node, hence converting it to an oxo-atom. While hydroxide is present in the reaction mixture, trifluoroacetate and chloride stem from different modulators, TFA and chloric acid, respectively. A subsequent partial Hessian vibrational analysis (PHVA)^271^,^272^ was carried out on a relevant part of the unit cell using TAMkin^273^ to calculate the free energies associated with the different capping mechanisms, showing that all of them were more favourable than removing one of the protons from the inorganic node. Moreover, two trifluoroacetate molecules were determined to be the most favourable capping mechanism (see Fig. 17).

In the same class of materials, a mechanistic investigation of aldol condensation has been undertaken, performing both periodic and extended cluster calculations on UiO-66 and UiO-66-NH_2_ to unravel the reaction mechanism at the OMSs.^216^ The extended cluster of the UiO-66 active site has been selected based on an optimised supercell with one BDC linker out of twelve missing. The model consisted of two adjacent open zirconium sites that were surrounded by four BDC linkers while the remaining seven ligands were substituted by formate. The calculations have been performed using the B3LYP exchange–correlation functional in the Gaussian09 package. In order to complement and properly account for the topology of the material, periodic DFT calculations were also performed using VASP. This study revealed that the Zr–O–Zr bridge has a bifunctional character in which the open zirconium sites act as Lewis acid sites and the bridging oxo-atoms act as strong Brønsted basic sites, capturing a proton during the reaction.

Structural linker vacancies do not only play a role in UiO-66-type materials, but may also influence the catalytic properties of MOFs comprising copper paddlewheels. In a 2012 study, St. Petkov *et al.* investigated the infrared (IR) spectrum of CO adsorbed in HKUST-1 or CuBTC, illustrating how computational spectroscopy may help in unravelling the nature of the active site.^274^ The IR spectrum revealed two CO bands, one of which could be assigned to the CO bound to the regular Cu^2+^ species, and one that seemed to stem from CO bound to irregular Cu^+^ species. St. Petkov *et al.* suggested that, when one out of four 1,3,5-benzenetricarboxylate (BTC) ligands connected to a copper dimer were to be removed, the excessive positive charge of the Cu^2+^ species would be compensated by Cu^+^. To validate that this linker vacancy indeed leads to the observed IR spectrum, DFT calculations on dimer cluster models with a different amount of BTC ligands were carried out using the B3LYP exchange–correlation functional. These quantum mechanical calculations unequivocally indicated that the experimentally observed IR spectrum can be explained by the adsorption of the mixed Cu^+^/Cu^2+^ dimer, exhibiting an electronic doublet state, and, moreover, that these reduced dimers are favourable adsorption sites for CO. This observation paves the way for employing the active site created in this fashion.

##### Class 2: OMSs with catalytically active terminating ligands

The inorganic nodes of the zirconium-based NU-1000, [Zr_6_O_4_(OH)_4_]^12+^ (hydrated) or [Zr_6_O_6_]^12+^ (dehydrated), are identical to those of UiO-66. However, instead of twelve structurally-defining BDC ligands, only eight of the twelve possible points of extensions are occupied by tetradentate TBAPy ligands, whereas the remaining four, located on the equatorial positions, are occupied by water/hydroxyl groups (see Fig. 16).^100^ This material was studied by Beyzavi *et al.* to elucidate the reaction mechanism of epoxide ring opening.^275^ However, they opted to exchange the Zr^4+^ cations with Hf^4+^, since the dissociation enthalpies of typical Hf–O *versus* Zr–O bonds indicate that hafnium is more oxophylic than zirconium and hence should function as a stronger Brønsted acid site. Periodic DFT calculations on both NU-1000(Zr) and NU-1000(Hf) were carried out using the PBE generalised gradient approximation exchange–correlation functional as implemented in VASP. To account for the solvation with trimethylsilyl azide (TMS-N_3_) during the reaction, the continuum solvent model was applied. The reaction mechanism was further studied on a NU-1000(Hf) cluster model in which two of the terminating phenyl groups of the original ligand were retained to preserve a good representation of the first coordination sphere of the Hf_6_ oxo-metallate node. It has been found that epoxide ring-opening on NU-1000(Hf) is Brønsted acid catalysed in which styrene oxide forms an association complex with the inorganic Hf_6_ node *via* hydrogen bonding derived from the terminating hydroxyl ligand, and that the binding energy of styrene oxide is stronger due to the π-stacking interaction with the organic ligands.

In a study by Kathalikkattil *et al.*, a zinc-based bio-MOF was successfully synthesised in the rare **lcy** topology.^276^ The zinc-glutamate (ZnGlu) MOF consists of zinc atoms that are fivefold coordinated with the structurally-defining glutamate ligands, whereas the sixth position in the zinc coordination sphere is occupied by coordinated water. Upon removal of this water molecule, a penta-coordinated zinc Lewis acid site is formed which allowed for the further interaction with propylene oxide for the cycloaddition of carbon dioxide. The full epoxidation mechanism was studied on a cluster model applying quantum mechanical calculations in the Jaguar code using the M06 functional.

A special subclass of MOFs consists of frameworks comprised of 1D inorganic chains, such as the [V=O]_∞_ chains in MIL-47(V) or the [M–OH]_∞_ chains in MIL-53(M),^277^,^278^ which are in both cases connected by BDC ligands. While these materials may be catalytically active when linker defects are created, the small μ_2_-oxo or μ_2_-hydroxo groups connecting the metal atoms in the inorganic chain may themselves also be catalytically active.^279^–^281^ Moreover, these materials, that often exhibit flexible behaviour under various stimuli, are characterised by 1D channels parallel to the 1D chains, which allows relatively large molecules to diffuse through the material and reach possible catalytically active centres. Ravon *et al.* investigated the acid strength of the catalytic centres in MIL-53(Al) and MIL-53(Ga), the latter material also named IM-19.^282^ For this purpose, the authors identified the nature of the intermediate in the Friedel–Crafts alkylation of different monosubstituted benzenes with *tert*-butylchloride and biphenyl and characterised the acid centres using DFT studies on the periodic model. The simulated adsorption IR peak positions and shifts of the CO probe molecule on the catalysts were in very good agreement with experimental data for the *ν*(OH) bands. The molecular modelling in the OH region of the material clearly confirmed the Brønsted acidity of MIL-53(Ga), although of mild strength. It has been proposed that the absence of catalytic activity for Friedel–Crafts alkylation in MIL-53(Al) may not only be attributed to the very low acidity, but could also be realised because of the location of the –OH groups in the inorganic chain, which are ordered in a straight fashion. In contrast, the tilted –OH groups in the MIL-53(Ga) inorganic chains induce a much stronger stabilisation of the passively charged intermediates, leading to a higher turnover. In the same class of materials, Vandichel *et al.* also investigated the creation of catalytically active open vanadium sites for the oxidation of cyclohexene using TBHP/water as oxidants. This catalyst showed a TON of 150, which was only slightly lower than the TON reported for the VO(acac)_2_ homogeneous catalyst (TON of 169).^280^,^281^

##### Class 3: OMSs due to topological restrictions

In this class of materials, OMSs are created because the topology of the material prohibits the complete saturation of the coordination sphere of the inorganic node. This inorganic node is in many cases the Cu_2_(CO_2_)_4_ paddlewheel or one of its isometallic analogues. Indeed, in these inorganic nodes, the metal centres adopt a square pyramidal geometry, and the metals are kept rigidly in the square. However, since many of these metal centres prefer a sixfold coordination, the two axial positions are often occupied by labile but coordinated ligands, or remain open.

**Fig. 18:**
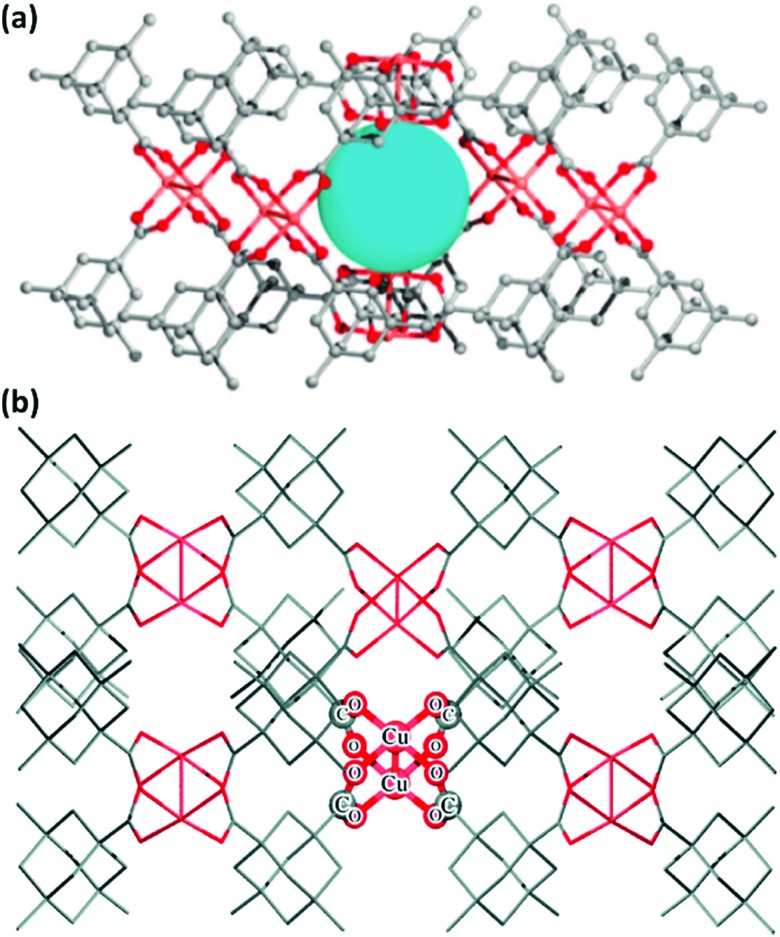
(a) MOF-11 model used in the work of Choomwattana *et al.*; (b) layers of the ONIOM model: high level (ball-and-stick model) and low level (line model). Figure adapted from ref. 283 with permission of the American Chemical Society, copyright 2008.

In MOF-11, the two labile ligands are typically water molecules which can easily be removed upon heating.^33^ Choomwattana *et al.* demonstrated that the open copper sites may act as a Lewis acid catalyst for the carbonyl-ene reaction between formaldehyde and propylene.^283^ The model of the MOF-11 catalyst has been described using the ONIOM method to account for the role of the whole framework in the adsorption of reactants.^209^ The copper paddlewheel, forming the active site for this type of catalysis, was described as an inner ONIOM layer and treated quantum mechanically (QM) with the B3LYP hybrid functional as indicated in Fig. 18. In contrast, the framework environment, which gives mostly van der Waals interactions due to confinement of the adsorbed species in the nanoporous material, was defined as the outer ONIOM layer and considered with molecular mechanics (MM) using the universal force field (UFF).^284^ This hybrid QM/MM approach is an effective trade-off between the desired accuracy and available computing resources. In the performed calculations, only the actively involved part of the copper paddlewheel and reacting molecules were optimised, while the remainder of the framework was kept at the crystallographic positions. For the carbonyl-ene reaction between formaldehyde and propylene, the authors proposed the concerted mechanism that comprises five steps. The role of the Lewis acidity of copper in MOF-11 was elucidated already in the first step by a high adsorption energy of formaldehyde which was –51.6 kJ mol^–1^ compared to –13.8 kJ mol^–1^ on a bare Cu^+^ model. Similar conclusions for the Cu-MOF-505 were drawn by Yadnum *et al.*^208^ By applying an analogous computational methodology as described above for the Mukaiyama aldol reaction, the authors proposed the Cu^+^ cation as the Lewis acid site responsible for the adsorption and activation of reacting molecules.

Another example of a carbonyl-ene type reaction, the citronellal cyclisation, was studied using HKUST-1 as the heterogeneous catalyst. Also this MOF consists of the same copper paddlewheel units as MOF-11 and Cu-MOF-505. Vandichel *et al.* used an ONIOM approach for HKUST-1,^285^ similar to the approach of Choomwattana *et al.* for MOF-11.^283^ However, Vandichel *et al.* applied first-principles calculations both in the high-level and low-level ONIOM layers, using the cluster-based Gaussian09 code, rather than applying force fields in the low-level layer. The clusters were extracted from periodic VASP calculations. This study unequivocally indicated that open copper sites act as Lewis acid sites, binding with the carbonyl oxygen from the citronellal molecule.

The efficiency of HKUST-1 has also been investigated for the Friedländer reaction,^286^ showing a higher activity than conventional zeolites or mesoporous materials such as H-BEA and (Al)SBA-15.^287^,^288^ This increase in catalytic activity has been assigned to a higher number of available undercoordinated metal ions in the paddlewheel units. To obtain insight in the reaction mechanism, DFT calculations have been conducted on both cluster and periodic models. Periodic calculations employing the PBE exchange–correlation functional as implemented in VASP were performed for the primitive cell containing 12 Cu^2+^ ions. The authors studied three cluster models by means of the paddlewheel model, the single copper site model, and the model accounting for two adjacent OMSs. To allow for the energy comparison with periodic simulations, also the cluster calculations have been carried out using both the B3LYP and PBE functionals in the Gaussian09 program. The mechanism mediating the Friedländer reaction depends on the catalyst character. Two different active sites were characterised, namely the Lewis acid (Cu^2+^ OMS in HKUST-1) and the Brønsted acid sites formed by the protons. The concerted effect of neighbouring copper sites was shown to be fundamental for the efficient catalysis of the rate determining reaction step.

**Fig. 19:**
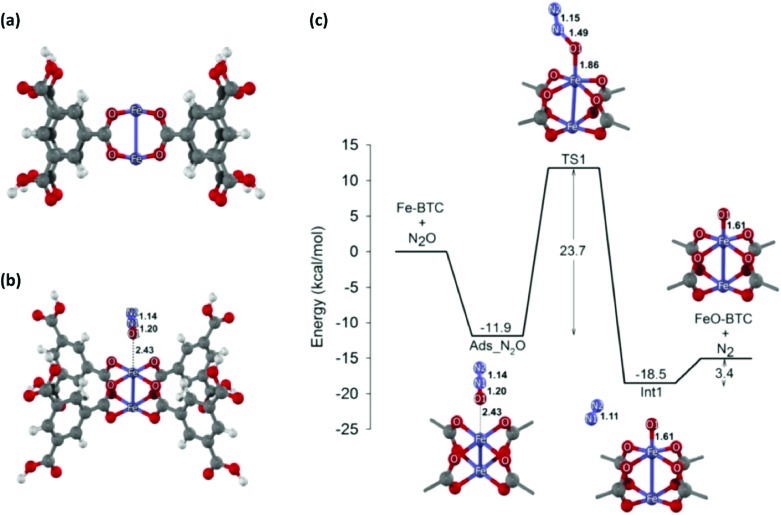
(a) and (b) Optimised structures of the (a) FeBTC model and (b) its interaction with nitrous oxide; (c) energy profile and some geometric parameters of reactants, intermediate, and transitions state involved in the decomposition of N_2_O over FeBTC (distances in Å). Figure adapted from ref. 289 with permission of Wiley, copyright 2016.

The iron exchanged analogue of HKUST-1, FeBTC, was studied by Maihom *et al.* for the catalytic ethylene epoxidation with nitrous oxide.^289^ By performing DFT calculations with the M06-L local functional as implemented in Gaussian09, they unveiled the reaction mechanism and underlying energy profile of the N_2_O decomposition and the subsequent ethylene epoxidation (Fig. 19). The authors concluded that the reaction is initiated by the decomposition of N_2_O to generate the active oxygen atom residing on the coordinatively unsaturated iron sites in the paddlewheel units. This is followed by the reaction of the ethylene molecule with this site, leading to the formation of the ethylenoxy intermediate, which can then form the final ethylene oxide. Moreover, they also showed that the activation barrier for acetaldehyde formation from the ethylenoxy intermediate is larger, leading to a preferential epoxidation reaction.

**Fig. 20:**
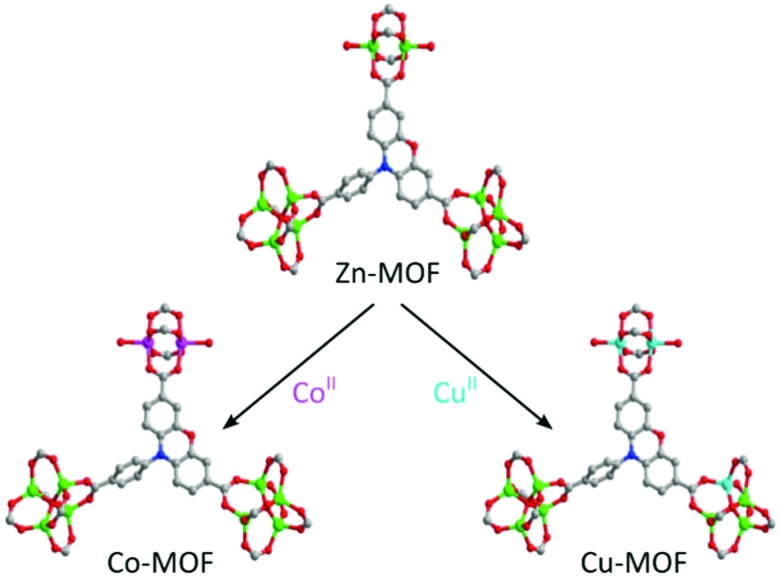
Illustration of the fragmental cluster change *via* metal cation exchange in a zinc paddlewheel MOF. Reproduced from ref. 290 with permission of Wiley, copyright 2016.

To further separate the paddlewheel units as active centres, bimetallic MOFs may be synthesised. Zou *et al.* synthesised the bimetallic MOF of Fig. 20, which is constructed of two types of inorganic nodes: zinc paddlewheels, Zn_2_(CO_2_)_4_, and tetrameric Zn_4_O(CO_2_)_6_ nodes, which are connected through a tricarboxylate ligand.^290^ Moreover, using selective post-synthetic metal exchange, the zinc centres in the paddlewheel units were exchanged with either copper or cobalt, while the zinc centres in the tetrameric nodes were largely preserved during the process. The performance of the so-obtained bimetallic MOFs for the chemical fixation of CO_2_ on epoxy propane to yield propylene carbonate was then assessed experimentally. From a theoretical point of view, this catalytic reaction is limited by the energetic mismatch of the highest occupied molecular orbital (HOMO) of the epoxy propane and the lowest unoccupied molecular orbital (LUMO) of CO_2_. DFT molecular dynamic simulations were conducted to explain the distinct catalytic performance of the bimetallic MOFs. By calculating the orbital energies using the LDA-PWC functional, the energy gap between the HOMO of the epoxy propane and the LUMO of CO_2_ was determined. The original zinc MOF represented the first MOF with open metal sites for the cycloaddition reaction due to the open zinc Lewis acid sites.

While this type of catalysis is often encountered in paddlewheel MOFs, other topologies may also give rise to topologically restricted OMSs. In MOF-74(M) or CPO-27(M), a 1D helical inorganic chain or rod is formed with composition [[O_2_M_2_](CO_2_)_2_]_∞_, where the metal centre M = Ni, Co, Zn, Mg, or Mn is sixfold coordinated, one of the coordination sites again being occupied by a labile molecule such as DMF which can easily be removed upon heating.^291^,^292^ The 1D chains are connected through tetradentate DOBDC linkers. The open metal sites, obtained when heating the material, result in a MOF with intrinsic framework basicity, as reported by Valvekens *et al.*^219^ MOF-74(M) or CPO-27(M) was considered as a catalyst in the Knoevenagel condensation and Michael conjugated addition reactions. It has been found that, among a large set of studied metals in MOF-74, the nickel-based material showed one of the strongest Lewis interactions, which was confirmed theoretically by calculated proton affinities (*vide supra*). In another work of Valvekens *et al.*, the base catalytic activity of the alkaline earth MOFs M_2_(BTC)(NO_3_)(DMF) with M = Ba or Sr has been studied.^293^ The defect structure shows a strong basicity indicating that alkaline earth ions are closely involved in the base catalytic activity. The basicity of the proposed Ba^2+^–O–Ba^2+^ motifs generated in the structures is close to those of the edge sites in BaO, as proven by calculating proton affinities on the generated active sites. In this case, Ba–MOF clusters were cut from the periodically optimised structures and the outer carboxylic units of the linkers were kept fixed during the cluster optimisation to mimic the rigidity of the framework. The iron analogue of this MOF, MOF-74(Fe) or CPO-27(Fe), was the subject of further study by Xiao *et al.*^294^ DFT and CASPT2 (Complete Active Space with Second-order Perturbation Theory) calculations were performed on a truncated model of the MOF to study the electronic structure of the cluster model. It has been proposed that the Fe(ii) centres can activate N_2_O, which then acts as a Lewis acid catalyst which may further activate the C–H bonds of ethane and convert it into ethanol and acetaldehyde using N_2_O as the terminal oxidant.

In the paddlewheel unit discussed above, the planar geometry results in the creation of OMSs. This geometry is not unique to the paddlewheel units, however, and is also found in the copper MOFs Cu(2-pymo)_2_ and Cu(im)_2_, in which the Cu^2+^ centres are bridged *via* the nitrogen atoms of azaheterocyclic compounds: respectively pyrimidine (pymo) and imidazole (im).^295^,^296^ Luz *et al.* have carried out first-principles DFT calculations to investigate the interaction between these MOFs and cumene-hydroperoxide.^74^ In order to get a deep insight into the structural and electronic properties of the two catalysts, the cluster models were selected consisting of a central Cu^2+^ cation surrounded by either four imidazole or four 2-hydroxypyrimidine molecules. Although these two MOFs have different abilities to decompose the hydroperoxide, for both MOFs the reaction takes place on a copper coordination vacancy that forms a Lewis acid site. Similar Lewis acid sites were identified in cobalt-based MOFs by Tonigold *et al.*^297^ and Tuci *et al.*,^298^ for which periodic DFT calculations have been performed probing their effective oxygen uptake. Both studies pointed towards the presence of oxo-species at the cobalt centre, indicating the Lewis acid nature of this metal.

#### D.2.2. Type II: catalysis at ligands: metalloporphyrins

Porphyrin rings and their structural analogues are attractive moieties to include in framework materials because of their versatility for catalytic purposes.^299^ The four nitrogen atoms at the centre of this ring may coordinate metal atoms such as titanium, chromium, cobalt, nickel, copper, zinc, or iron, the latter also termed heme. In all these cases, the metal atoms are coordinated by the ring in a square planar fashion, such that their axial positions are unsaturated which can be exploited for catalysis (Fig. 1, **type II**). While many porphyrin-based framework materials, including both MOFs^300^–^302^ and COFs,^142^,^303^ have been synthesised, theoretical studies on these materials are currently limited because of the necessarily extended size of porphyrin-based ligands, which may contain about a hundred atoms and are often fourfold coordinated. Therefore, the active site for catalysis is often reduced to a few tens of atoms, and approximate schemes are applied to study these catalytic sites. However, thanks to the increasing computational possibilities, we envisage a gargantuan body of theoretical work on these materials in the very near future.

**Fig. 21:**
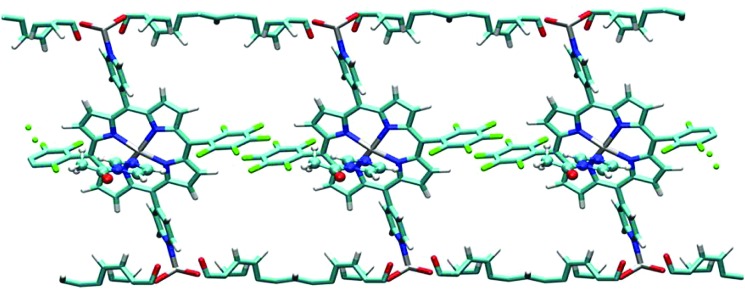
Representation of *N*-acetylimidazole molecules at active sites of ZnPO-MOF. Reprinted from ref. 304 with permission of the American Chemical Society, copyright 2012.

As early as 2012, Roy *et al.* studied the acyl-transfer reaction between 3-pyridylcarbinol and *N*-acetylimidazole on ZnPO-MOF,^304^ a MOF consisting of zinc dimers which are either linked by the tetradentate 1,2,4,5-tetrakis(4-carboxyphenyl)benzene ligand or a bidentate porphyrin-based ligand, embedding a catalytically active zinc atom in the latter (Fig. 21).^305^ Note that, while the porphyrin ligand is normally fourfold coordinated, two of those coordinating species are terminating pentafluorophenyl moieties, limiting the size of the MOF's unit cell. The authors proposed a three-step approach to calculate the rate enhancement of this reaction due to preconcentration of reactants at the zinc-porphyrin active sites. In the first step, the binding energies of solvent, reactants, and products to the active site of ZnPO-MOF were calculated using a DFT-based approach. In the next step, these results were used to obtain the equilibrium constants for the binding of the reactant and the dissociation of the product at the active site. Lastly, the authors derived a kinetic model to calculate the importance of reactant preconcentration in this system. The DFT investigation with the PBE functional of the framework-promoted reaction at the active metal site has been performed using the full atomic structure of the ZnPO-MOF in the VASP code, which is possible thanks to the reduced twofold coordination of the porphyrin.

When a higher level of theory is needed, the porphyrin linker needs to be terminated as a cluster to ensure the calculations are computationally feasible. Maitarad *et al.* employed such a porphyrin cluster model to study the adsorption of N_2_O over metalloporphyrins with different metals.^306^ Among the plethora of studied metals, titanium-porphyrin in the triplet ground state was the most active for N_2_O adsorption. This material was further assessed for a direct decomposition of N_2_O to N_2_ and O_2_. An overall reaction mechanism involving three N_2_O molecules on titanium-porphyrin was proposed, demonstrating that titanium acts as a Lewis acid site and is coordinated by the oxygen atom. All of the electronic structure calculations were carried out using the Gaussian09 program employing DFT with the M06-L local functional.

**Fig. 22:**
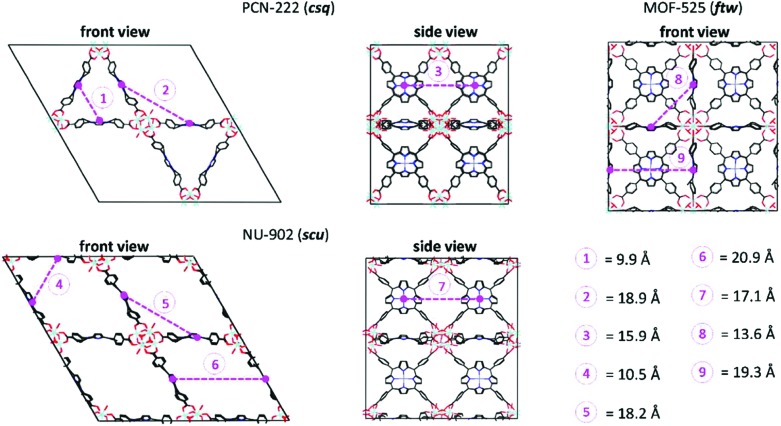
The nine different pairs of porphyrin sites with their respective centre-to-centre Zn–Zn distances for pairs in PCN-222 (1–3), NU-902 (4–7), and MOF-525 (8–9). Figure reproduced from ref. 307 with permission of the American Chemical Society, copyright 2016.

While the aforementioned studies focus on the presence of one porphyrin-based ligand, Deria *et al.* envisaged to determine how the topology of a MOF, and hence the relative orientation of the porphyrin-based ligands, may alter the catalytic properties of a MOF.^307^ For this study, three MOFs were selected which are all formed by connecting zirconium-based clusters with zinc-embedded porphyrin ligands: PCN-222 (PCN = porous coordination network) or MOF-545,^308^,^309^ MOF-525,^309^ and the newly synthesised NU-902,^307^ which synthesise respectively in the **csq**, **ftw**, and **scu** topologies. The authors postulated that the MOFs' performance in the Lewis acid catalysed acyl transfer reaction between pyridylcarbinol and *N*-acylimidazole depends on the relative spatial organisation of the zinc-containing porphyrin ligands, since the pairs of porphyrin sites should position the acyl-group donor and acceptor species at suitable distances and relative orientations to facilitate the formation of the transition state. At first instance, the authors determined the nine configurations in these three MOFs for which the zinc atoms embedded in the porphyrin ligands were the closest, which are shown in Fig. 22. Molecular mechanics simulations using UFF were applied to calculate the strain energy resulting from positioning the isolated intermediate in between the porphyrin ligands, and the two distinct configurations with the smallest strain energy were selected for further study. The porphyrin cores of these two configurations were then optimised using the B3LYP exchange–correlation functional in the DFT paradigm, accurately determining that the configuration of porphyrin sites in MOF-525 optimises the acyl transfer reaction under study.

#### D.2.3. Type III: catalysis at reactive functional groups

Within the third type of single active sites, functional groups are covalently bound to the linker. Organic groups can be bound both during synthesis or post-synthetically, but the inorganic groups can only be added post-synthetically, a procedure which is termed post-synthetic metalation.^310^ Due to the different catalytic nature of metal centres and organic groups, the discussion will be subdivided in these two subclasses.

##### Class 1: inorganic groups

As any post-synthetic functionalisation, post-synthetic metalation requires the framework to be sufficiently stable to retain its porosity and crystallinity after the process. As a result, post-synthetic metalation is often carried out on the most stable MOFs, such as the zirconium-based UiO-66 and NU-1000 and their hafnium analogues, as well as on the prototypical MOF-5.^311^

While pristine MOF-5 is not catalytically active, Maihom *et al.* were guided by experimental findings when they added a copper alkoxide group to the BDC linker to construct a single active site for the production of formic acid from H_2_ and CO_2_.^312^ The selected cluster model consisted of two Zn_4_O inorganic nodes connected by one copper-alkoxide functionalised linker, the simplest model still capturing the linker modification and its direct environment. The choice for cluster calculations was motivated by the large size of the MOF-5 cell. In all DFT simulations, the M06-L local functional was used. Further, the authors examined both the concerted and stepwise mechanism of the carbon dioxide hydrogenation to formic acid over the copper-alkoxide functionalised MOF-5. They revealed that the reaction proceeds through the stepwise mechanism and that the activity of the reaction increases with the electron-donating group substitution, as verified by substituting the two nonfunctionalised positions of the functionalised linker by nitro or amino groups.

While the metalation above was carried out on the organic building block of the MOF, metal complexes can also be supported by the inorganic nodes. The inorganic nodes of NU-1000 and UiO-66 were identified as essential catalyst supports for metal complexes such as Ir(CO)_2_ in the work of Yang *et al.*^313^ These complexes were subsequently studied for the catalytic hydrogenation and dimerisation of ethylene. The zirconium nodes of these MOFs were modelled as finite clusters that were extracted from the periodic unit cells after optimisation at the PBE level of theory. To determine the structure of the iridium-supporting sites, cluster DFT calculations employing the M06-L local functional were applied and various calculated bands have been compared with the ones observed experimentally. It has been noted that the nature of the MOF support influences the transition states and activation energies for the various catalytic reactions. The authors explored different competing reaction mechanisms for ethylene dimerisation and proposed the one in which chemisorption of ethane takes place on the iridium sites. Another iridium complex was also computationally embedded on UiO-66, UiO-67, and NU-1000 by the same authors to study ethylene conversion.^314^ For the UiO-66 type materials, one linker was removed and the open zirconium metal sites were terminated by –OH and –OH_2_ groups. For the optimisation, the same computational methodology as in the previous work has been applied. Once more it has been proven that DFT calculations provided detailed insights into the structure of the catalyst, the reactivity, and the catalytic properties of the iridium centre bonded to the MOF supports.

The accuracy of different levels of theory on the zirconium-based NU-1000 was studied by Bernales *et al.*^315^ The authors applied both DFT with the M06-L local functional as well as higher-level multireference simulations to study the atomic layer deposition of nickel and cobalt and the subsequent ethylene dimerisation mechanism on the inorganic nodes of NU-1000. The inorganic NU-1000 nodes contain reactive –OH and –OH_2_ groups on which the transition metals were deposited. The extended cluster model consisting of benzoate and formate groups was selected and optimised using the M06-L functional as implemented in Gaussian09. The key reaction intermediates identified by DFT were further characterised by applying the complete active space self-consistent field (CASSCF) level of theory, followed by second-order perturbation theory (CASPT2) calculations in the MOLCAS package.^316^ The reaction mechanism proceeds *via* an ethyl-nickel/cobalt intermediate and the formation of the ethyl–metal bond is the rate-determining step in both cases. The highest catalytic activity was found for the nickel-containing NU-1000. A similar embedded procedure has been used by Klet *et al.* to study the single-site, highly electrophilic organozirconium ZrBn_4_ (Bn = benzyl) catalyst supported on the hafnium analogue of NU-1000.^317^ A zirconium–monobenzyl species has been discovered as the lowest product on the reaction pathway. This site was further investigated as catalytically active for olefin polymerisation. The periodic structure and the cluster model calculations were performed employing the PBE and M06-L functionals, respectively.

In a subsequent study by the same authors on the zirconium-based NU-1000, the accuracy of different DFT functionals for acceptorless alcohol dehydrogenation was assessed.^318^ This reaction was catalysed by depositing different first-row transition metals on the NU-1000 inorganic nodes. A neutral cluster model involving one inorganic node and eight organic linkers was used and the simulations were carried out using the M06-L local functional as benchmark functional, comparing the results with other functionals such as PBE, B3PW91, and B3LYP with and without dispersion corrections.^270^ The incorporation of a metal on the inorganic support was achieved by removing two acid hydrogens belonging to the –OH and –OH_2_ groups. A three-step mechanism for the acceptorless alcohol dehydrogenation was proposed, comprising of a proton transfer, a β-hydride elimination, and a H–H bond formation. It has been concluded that the metal embedded on the inorganic node acts as a Lewis acid site on which two reacting cyclohexanol molecules can be adsorbed. Furthermore, the Brønsted catalytic activity of the support was confirmed for the proton transfer and the H–H bond formation step.

While the attention of the aforementioned studies is aimed at describing the metalation process itself, leaching, *i.e.* the removal of the inorganic group as a result of competitive reactions at the support site, may still pose an important problem. In a study by Noh *et al.*, a molybdenum oxide was deposited on the zirconium inorganic nodes of NU-1000 using a solvothermal deposition method.^319^ By using the molybdenum-containing inorganic node terminated by benzoate groups as a cluster model for DFT calculations using the M06-L local functional as implemented in Gaussian09, the authors showed that the regeneration of the inorganic node is strongly endergonic, confirming the stability of this catalyst towards leaching.

##### Class 2: organic groups

Functionalisation of MOFs with smaller organic groups, such as amino or nitro groups, can be achieved by adding the appropriate carboxylic acids to the synthetic mixture. However, larger organic functional groups are often added post-synthetically *via* click reactions.

An example of a MOF with small functional groups, IRMOF-3, was discussed by Gascon *et al.*^320^ and Cortese *et al.*^321^ IRMOF-3 is the MOF-5 analogue in which each BDC ligand is functionalised with one amino group. Cortese and colleagues studied the structural and electronic properties of IRMOF-3 during the catalysed Knoevenagel condensation of benzaldehyde and ethyl-cyanoacetate. To this end, they applied both purely quantum mechanical calculations using the DFT paradigm with the BP86, B3LYP and MPWB1K functionals as implemented in Gaussian03, as well as the ONIOM approach with semi-empirical AM1 or PM3 methods at the lower layer and the aforementioned DFT functionals at the higher layer. Second-order Møller–Plesset (MP2) perturbation theory calculations were also carried out to determine the proton affinity of the amine derivative models. To unravel the reaction mechanism, a cluster model consisting of two Zn_4_O inorganic bricks bridged together with the amino-functionalised linker was applied for the high-level calculations, a cluster analogous to the MOF-5 cluster used by Maihom *et al.*^312^ In the mechanism proposed by the authors, the reaction is base-catalysed and occurs on the amino group present on the linker, revealing imines as important intermediates.

**Fig. 23:**
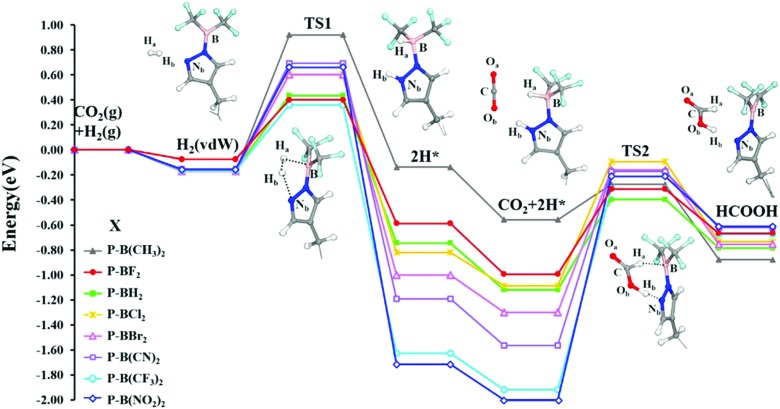
Potential energy profiles (0 K, no ZPE correction) for CO_2_ hydrogenation in UiO-66-X. Key structures are shown in the diagram for the P-B(CF_3_)_2_ functional group as examples. Figure reproduced from ref. 322 with permission from the American Chemical Society, copyright 2015.

For the second class of functional groups, requiring post-synthetic modification of the linker, Ye *et al.* proposed a possible pathway for the creation of UiO-66 with different extended organic groups containing Lewis pair moieties.^317^ In this work and the subsequent large-scale screening of functional groups,^322^ the authors investigated the performance of eight functional groups based on 1-(difluoroboranyl)-4-methylpyrazole, containing both Lewis acid and base sites, for the catalytic hydrogenation of CO_2_. All the considered functional groups are composed of boron as the Lewis acid site and nitrogen as the Lewis base site. The periodic DFT calculations were performed with the PBE functional as implemented in CP2K, and evidenced that the reaction mechanism always proceeds through a two-step mechanism based on the heterolytic dissociation of H_2_ on the Lewis pairs (Fig. 23). This screening study revealed energetic barriers which are among the lowest reported for the nonelectrolytic reduction of CO_2_ with H_2_. Moreover, the authors proposed two Brønsted–Evans–Polanyi relationships for this reaction, one relating to the barrier for the concerted addition of a hydride and a proton to CO_2_, and another for the recombination of the hydride and the proton to produce H_2_.

Finally, MOFs exist for which the organic functional group acts as a second active site, complementing an already existing active site in the nonfunctionalised MOF. For instance, Lescouet *et al.* studied the activity of the amino-functionalised MIL-68(In), for which the indium metal centres act as Brønsted acid sites, while the amino groups are Lewis basic sites.^323^ MIL-68(In) is the indium analogue of the vanadium-containing MIL-68, which is comprised of 1D inorganic [V(OH)]_∞_ chains connected *via* BDC ligands.^324^ The performance of MIL-68(In)-NH_2_ as a heterogeneous catalyst for the synthesis of styrene carbonate from styrene oxide and CO_2_ was studied both experimentally and theoretically. For the theoretical study, periodic structures of MIL-68(In)-NH_2_ were optimised with DFT using the PBE functional as implemented in VASP, including dispersion corrections following the DFT-D2 scheme of Grimme.^245^ The authors concluded that the acidity of the amino-functionalised MIL-68(In) is larger than its nonfunctionalised counterpart by calculating the adsorption energies of the basic probe molecules CO and NH_3_, a synergetic effect that is also encountered in other studies mentioned in this review.^76^,^216^

## E. Single site analysis by IR spectroscopy in MOFs

One of the major challenges in single site heterogeneous catalysis resides on the actual demonstration of the existence of such active sites. In this sense, advanced characterisation techniques, especially IR spectroscopy, are instrumental. As already mentioned, the accessible OMSs of MOFs can provide interesting centres for interactions with different molecules and represent the active sites where the chemical properties of such materials reside.^38^,^325^ Therefore, their precise investigation is of paramount importance, and among the different available techniques for this kind of materials, IR spectroscopy has a key role for unravelling their characteristics. Being sensitive to the molecular vibrations, IR spectroscopy can provide valuable and pertinent information on the adsorption sites and modes on a surface, either directly or *via* the adsorption of adapted probe molecules.^326^ Physical–chemical properties of the sites can be described and ranked *versus* reference compounds, drawing a picture of acidity, basicity, and redox properties in terms of strength and site concentrations.^327^ To characterise these features, a limited number of molecular probes have been used. We will classify them following the criteria they have been used for.

### E.1. CUS typology and acidity characterisation

CO is the typical example of a probe that, due to its small size and interaction sensitivity with a cationic or a metallic site, can provide a set of information about the host material. MIL-100^38^ and HKUST-1^328^,^329^ were the first MOFs to be investigated by this methodology. CO adsorption on MIL-100/101(Cr) is an example of the IR contribution to the identification of the potential active sites in MOFs.^4^,^38^,^330^ Thanks to the presence of three *ν*(CO) bands (observed at 2207, 2200 and 2193 cm^–1^), it was shown that the Cr^3+^ sites are heterogeneous, due to 2, 1 or no residual (from synthesis) fluoride ions on the metallic trimers, respectively, in the neighborhood of each Cr^3+^ OMS.^38^ The quantitative analysis also provided the number of expected free Cr^3+^ sites in the activated compound (3.5 mmol g^–1^), considering that one corner of the three octahedra is occupied by one anion. In MIL-101(Cr), this methodology allowed identifying these sites as the grafting centres of catalytically active sites.^4^ In some cases, the weak interaction of CO with the substrate needs to be enhanced by adsorption at low temperature. For example, in the case of HKUST-1, a larger amount of coordinate carbonyl species was observed at liquid nitrogen temperature than at room temperature, depending on the CO partial pressure on both Cu^I^ and Cu^II^ sites.^331^ With the assistance of an isotopic ^12^CO/^13^CO mixture, it was possible to conclude that part of the Cu^2+^ ions in a similar Basolite C300 sample are highly coordinatively unsaturated and can adsorb more than one CO molecule, which is important for both a correct quantification of the exposed sites and for rightly evaluating their hosting properties towards guest molecules.^332^ CO was found particularly useful for the characterisation of divalent and trivalent iron sites in MIL-100(Fe): at room temperature, CO does not interact strongly with Fe^3+^, providing only a weak band at 2190 cm^–1^, whereas Fe^2+^ leads to the appearance of two bands at 2182 and 2173 cm^–1^.^5^ At 100 K, these bands shift to 2179 and 2170 cm^–1^ for Fe^2+^, and between 2192 and 2173 cm^–1^ for Fe^3+^ sites, allowing a quantitative analysis of the site concentration, with relative intensities depending on the activation treatment.^81^ In CPO-27(Ni), CO adsorption gave rise to a very intense band at 2178 cm^–1^ due to the formation of Ni^2+^···CO complexes, persisting at room temperature, but being reversibly desorbed upon prolonged evacuation.^333^ A similar band was also observed after CO introduction on MOF-74(Mg).^334^ These studies also supported the assignments for CO adsorption in the mixed MIL-127(Fe,Ni).^335^ Thanks to CO gas sorption analysis combined with adsorption microcalorimetry, the much higher sorption capacities of the mixed metal compound were highlighted with respect to the pure iron sample, as well as a higher tendency to form Fe(ii) sites. Again, in agreement with previous experiences and calculations,^336^ CO could evidence the differences in the electronegativity of divalent cations substituted in the polycarboxylate structure, giving rise to an affinity ranking for the probe towards the various divalent species with the trend Ni^2+^ > Co^2+^ > Fe^2+^ > Mg^2+^,^335^ in agreement with the differences in the enthalpies of adsorption that lie from about –30 to –50 kJ mol^–1^ at low coverage. Combining data obtained by CO or ^15^N_2_ adsorption recently proved to be useful to distinguish free and H-bonded hydroxyls in the case of MIL-53(Al) and NH_2_-MIL-53(Al).^337^

Pyridine is in general the molecule widely used to probe the Lewis acid strength on oxide surfaces, through the wavenumbers of its *ν*_8a_ and *ν*_19b_ bands. Most of the time, these modes are useless for MOFs, being overlapped by very strong bands due to carboxylate and ring vibrations. Nevertheless, other bands can be used in the 1000–1100 cm^–1^ range. For example, pyridine adsorption on MIL-100(Fe) activated at different temperatures gave rise to a set of bands at 1008–1014, 1043 and 1070 cm^–1^, which were assigned to *ν*_1_, *ν*_12_ and *ν*_18a_ ring modes of the probe. They present a significant blue shift with respect to the liquid phase and can be correlated with the site acidity.^81^ It is worth to remark that the use of this probe molecule has allowed evidencing the presence of additional Brønsted acid sites in Basolite F300 with respect to MIL-100, responsible for a different catalytic activity of these two solids.^82^

Another valuable probe for the characterisation of acidity in porous materials is acetonitrile, properly used in its deuterated form CD_3_CN. For example, the acidity of the Ln^3+^ sites in MIL-103(Ln) was investigated by this probe. The position of the *ν*(CN) mode of the coordinated acetonitrile (2283, 2287 and 2290 cm^–1^ for Ln = La, Eu, and Dy, respectively) was directly related to the strength of the lanthanide–nitrile bond, proportional to the polarising power of the cation.^338^ Acetonitrile was used to probe and quantify the acidity of zirconium-based MOFs.^114^,^339^,^340^ In the case of UiO-66(Zr) and UiO-66(Zr)-(COOH)_*x*_, acetonitrile allows distinguishing the presence of Lewis and Brønsted (associated with hydroxyls) acid sites through the *ν*(CN) vibration bands observed at 2304 and 2275 cm^–1^, respectively, and also provides the concentration and the strength of the sites *versus* the dehydration process and the presence of the functional groups.^340^ In a similar way, acetonitrile probed the presence of Lewis acid sites in the aluminium fumarate A520, having a strength intermediate between that of MIL-100(Al) and those of MIL-100(Cr) or MIL-100(Fe). On the counterpart, CO adsorption at low temperature demonstrates that the Brønsted sites present a milder acidity with respect to those in MIL-100(Cr).^341^ It is worth remarking that sometimes probes may present a different behaviour on the sites due to their specific interactions. This is the case, for example, in MIL-100(Al), where CD_3_CN indicates a strong acidity of aluminium OMSs, close to that reported for silica–alumina, whereas CO reveals a medium acid strength, as that reported for unsaturated sites in alumina. This contradictory result is particular to the MOF: the small diameter of the Al^3+^ cation increases the shielding effect of the neighbouring carboxyl oxygen atoms inducing a lower Al^3+^ OMS accessibility.^85^ This phenomenon shows that it is important to use and compare different probe molecules and compare their results when investigating a property of a material by IR spectroscopy, to avoid biased interpretations. When the spectroscopic study is related to the catalytic properties of the material, the best probe remains the reactant itself.

### E.2. Cationic species and their oxidation degree

Complementary information on MOF sites can be provided by NO, but band assignment is not always straightforward. In the case of HKUST-1, for example, the formation of Cu^2+^···NO adducts was indicated upon NO addition by a band at 1887 cm^–1^. In spite of a strong interaction energy, the nitrosyls appeared at a position relatively close to the gas phase due to the competitive effects of electrostatic polarisation and σ donation on the one hand, and the opposite π-back-donation on the other hand, as for CO adducts on Ni^2+^.^329^ In the case of CPO-27(Ni), both IR and Raman indicated the formation of linear/tilted Ni(ii)···NO species thanks to the appearance of a very strong and stable band at 1847 cm^–1^.^56^ A thorough characterisation of the presence and interchange between Cu^2+^/Cu^+^ sites (redox properties) in HKUST-1 was possible by coadsorbing CO and NO, then complementing these evidences by CO_2_, NO_2_, and methanol probes.^342^ Conversely, NO was fundamental as a probe to quantify the amount of Fe^3+^ and Fe^2+^ sites in iron carboxylates such as MIL-88.^343^ In MIL-100(Fe), nitrosyls were characterised by a band at 1901 cm^–1^ on Fe^III^ OMSs and by a doublet at 1842 and 1828 cm^–1^ on Fe^II^ OMSs.^72^ The stronger adsorption of NO with respect to CO on the divalent OMSs allowed for a precise measure of the Fe^2+^/Fe^3+^ ratio, as well as their role in the propene/propane separation.^56^ A similar approach permitted the quantitative analysis of accessible iron and nickel sites in MIL-127(Fe,Ni) as well.^335^ NO was also used to characterise the vanadium oxidation degrees in MIL-100(V), a material with promising redox catalytic properties.^83^

We have already evoked above the use of methanol as a probe molecule to ascertain the oxidation state of copper in HKUST-1. The invaluable importance of such a probe is highlighted in a study of UiO-66(Zr,Ce), where methanol clearly demonstrated the insertion of cerium in the hosting structure, as well as the presence of low amounts of Ce^3+^.^97^ Moreover, methanol behaved also as a reacting agent, undergoing a catalytic decomposition to CO_2_, hence demonstrating the unique properties of the new material due to a combination of defects and redox activity upon cerium substitution.^97^

### E.3. CO_2_-specific interactions

CO_2_ is an amphoteric molecule which can be used to probe both acidity and basicity,^327^ but in the case of MOFs carbon dioxide is a complementary probe to characterise acidic sites. The most interesting results on CO_2_ adsorption were found in MIL-100(Cr) and MIL-101(Cr), where the formation of CO_2_-coordinated species on Lewis acid sites gave rise to strong *ν*_3_ bands situated at 2351 and 2348 cm^–1^, respectively. The lower wavenumber position for the adsorbates in the latter compounds indicated a weaker interaction, as confirmed by calorimetric studies, and hence also for an easier reactivation of the material after carbon dioxide sequestration. This property was considered extremely important, being associated with an amount of CO_2_ filling in MIL-101(Cr) in mild conditions that was found the highest for all the known materials, so that it could be considered as a target solid in pressure swing adsorption (PSA) applications, being furthermore stable after several adsorption/desorption cycles and in the presence of moisture, as underlined by IR spectra.^84^ In another structure, such as the porous chromium(iii) terephthalate MIL-53(Cr), the adsorption mode of CO_2_ at low coverage was identified using IR spectroscopy: the red shift of the *ν*_3_ band and the splitting of the *ν*_2_ mode of CO_2_ in addition to the shifts of the *ν*(OH) and *δ*(OH) bands of the MIL-53(Cr) hydroxyl groups provided evidence that carbon dioxide acts as an electron-acceptor *via* the interaction of its carbon atom with the oxygen atoms of the framework's Cr(OH) inorganic chain. That was the first example of such an interaction between CO_2_ and bridged –OH groups in a solid.^344^ When CO_2_ was adsorbed at room temperature in MIL-53(Cr), an additional phenomenon was observed: the gas isothermal uptake proceeded *via* a plateau while the desorption occurred with hysteresis. *In situ* X-ray diffraction (XRD) and IR experiments demonstrated that this unusual effect was associated to a breathing behaviour of the hybrid structure.^345^ Interestingly, in the case of the MIL-53(Fe)-X functionalised MOFs, it is observed that the carbon atom of the CO_2_ molecule interacts preferentially with the oxygen atom of the carboxylate group, contrarily with what is predicted for the non-modified MIL-53(Fe), hence revealing that the functionalisation does not provide an expected additional X···CO_2_ interaction but rather a modulation of the interaction with the preferential μ_2_-OH adsorption sites.^346^ Similar studies on CO_2_ complexes on MIL-53(Al) and MIL-53(Al)-NH_2_ were also performed by Mihaylov and co-workers^332^ Also the linear chain coordination polymer Ni-DBM-BPY presented CO_2_ and CH_4_ adsorption behaviours typically associated with flexible MOFs. A detailed correlation between the structural phase transition in the material and the sorption of carbon dioxide was performed by monitoring *in situ* the adsorption of the gas *via* ATR-FTIR spectroscopy (attenuated total reflectance Fourier transform infrared spectroscopy), using the methodology described above.^347^

### E.4. Basicity

Propyne was used to probe the basicity of sites in MOFs: in the case of MIL-100(Fe), independently from the oxidation state of iron, only weak interactions of the molecule with the adsorption sites were observed, witnessing for a very weak basicity of the oxygen atoms around the metal sites.^81^ In the case of flexible MIL-53(Fe)–X MOFs, the interactions are very diversified, but once more IR spectroscopy (with the help of DFT calculations) can draw a view of the propyne molecule adsorbed *via* the C≡C bond or the hydrogen bonding of the terminal proton.^348^ In the same way, acetylene was used on CPO-27 to probe the basicity of the oxygen atoms.^349^

### E.5. Hydrogen as a probe for cations and ligands

Hydrogen is also an interesting probe having specific properties. Upon dihydrogen adsorption on HKUST-1 at 20 K, IR spectra showed two main bands at 4097 and 4090 cm^–1^. After time, the two bands shifted and interconverted (isosbestic point), and were replaced by other components (4137, 4140 and 4148 cm^–1^). This was interpreted as one of the few examples of *in situ* evidence of a single site catalysed *ortho*–*para* conversion of H_2_ in the adsorbed state, indicating that Cu^2+^ in HKUST-1 acts as a spin catalyst.^329^ On MOF-5, this *ortho*–*para* conversion started at higher temperatures, and diffuse reflectance infrared spectroscopy revealed at least three distinct binding sites upon adsorption at low temperature, with site-specific energies ranging from 2.5 to 4 kJ mol^–1^.^350^ In CPO-27(Ni), H_2_ adsorption started to be observed already at 180 K, giving rise to bands in two distinct regions (4010–4040 and 4110–4150 cm^–1^), the first associated with hydrogen interacting with Ni^2+^, while the second region was assigned to H_2_ adsorbed on ligands. In particular, the interaction of hydrogen with Ni^2+^ sites produced a doublet at 4035 and 4028 cm^–1^, due to Ni^2+^···H_2_ complexes belonging to the heterogeneity in the first coordination sphere of the oxygen atoms around nickel.^351^ Chavan *et al.* extended the study to the isostructural CPO-27(M) (M = Mg, Mn, Co, Zn). The strongest perturbation of the H_2_ vibrational frequency was shown to be due to the interaction with an OMS, and a direct correlation between the ionic radii of the metal cation and the H_2_ interaction energy was found in MOFs of the same topology.^352^ Drenchev *et al.* specified the infrared signature of hydrogen adsorbed on CPO-27(Ni) and the site competition with CO.^353^ Nijem *et al.* performed hydrogen adsorption at 300 K and high pressures (27–55 bar), followed by IR spectroscopy, on several MOF prototypes: Zn, Ni or Cu(BDC)(TED)_0.5_, Mn or Ni_3_(HCOO)_6_, Zn_2_(BPDC)_2_(BPEE) (TED = triethylenediamine, BPDC = 4,4′-biphenyldicarboxylate, BPEE = 1,2-(bipyridyl)ethane). These experiments highlighted the relevance of IR spectroscopy to determine the type and arrangement of ligands in the structure of MOFs.^354^ A thorough description of the structural and thermodynamic aspects of H_2_ adsorption at the strongest binding sites in Mn–, Fe–, and Cu–BTT (BTT = 1,3,5-benzenetristetrazolate) samples was obtained by combining IR experiments with a detailed DFT study.^355^ The effect of substitutions at the metal cluster (metal ion and anion within the tetranuclear cluster) was discussed, showing that the configuration of this unit indeed plays an important role in determining the affinity of the framework toward H_2_. This study highlighted the importance of a combined experimental and theoretical approach to the design and synthesis of new frameworks for H_2_ storage applications.

Other kinds of probe molecules have been used to characterise the OMSs of MOFs by IR spectroscopy, adapting the type of the guest with respect to the quality of the host and the researched interaction. But we cannot be exhaustive in this kind of review. What is important to underline is that IR spectroscopy of probe molecule adsorption constitutes a unique way for characterising qualitatively and quantitatively the OMSs in the structures, as well as their physical–chemical properties.

## F. Cooperative single-site catalysis with MOFs: one-pot tandem and multicomponent coupling reactions

In our recent perspective article, we envisaged the use of MOFs as (multifunctional) catalysts for sequential tandem reactions – including multicomponent coupling reactions – as one potential niche of application for this family of compounds in which MOFs have the potential to display interesting advantages with respect to other potential competitors such as homogeneous catalysts, zeolites, and other inorganic materials.^32^ Advancements in this field have been the subject of several reviews recently.^356^,^357^ Probably the type of multifunctional MOF-based catalysts studied most extensively so far consists of metal nanoparticles encapsulated in MOFs. Strategies for preparing these systems usually rely on techniques such as chemical vapour infiltration^358^ or impregnation^359^ of suitable metallic precursors followed by reduction, or either on nucleated synthesis in which a MOF is formed around pre-formed metal nanoparticles.^360^ A bunch of examples exist in which the catalytic activity of the encapsulated nanoparticles is then combined with active sites located at the MOF nodes or linkers, yielding multifunctional catalytic systems that can be successfully applied to sequential tandem reactions.^77^,^361^,^362^ However, given the scope of this review, only examples on (multifunctional) MOF catalysts based on single-site engineering are discussed here, thus excluding examples in which the catalytic activity arises from metal nanoparticles encapsulated inside the MOF pores. For a more general view of this type of compounds, the reader is referred to some recent reviews dealing with this type of composite materials.^363^,^364^

When dealing with single-site MOFs, various situations are possible, depending on whether the active centres are located at the metallic nodes, at the organic linkers, or a combination of both, as depicted in Fig. 1. Therefore, recent advances on the use of these compounds are reviewed based on two main scenarios: one-pot multicomponent coupling reactions and one-pot multistep sequential (or tandem) reactions. In spite of being conceptually different, both types of catalytic schemes represent a process intensification strategy, aiming at reducing the number of separation/purification steps of intermediate products, thereby reducing the energy consumption and the amount of solvents used and by-products generated in the synthesis of the target compounds. This leads to a significant and highly desirable improvement of the atom and process economies while minimising its environmental impact.

### F.1. One-pot tandem reactions

#### F.1.1. Acid–base bifunctional catalysts

**Scheme 1:**
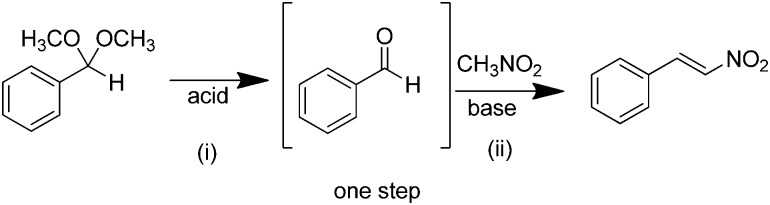
Tandem reaction showing (i) the deacetalisation of benzaldehyde dimethyl acetal and (ii) the Henry condensation of benzaldehyde with nitromethane.

One of the preferred tandem processes to evaluate the catalytic activity of MOFs comprising acid and basic single sites is the deacetalisation of benzaldehyde dimethyl acetal followed by the Henry condensation in which the resulting benzaldehyde couples with nitromethane (Scheme 1). Although the two individual steps are separately not very challenging and there are numerous Brønsted and Lewis acids and bases that can catalyse them independently, the point is that the coupling of the two reactions in a tandem process would require the simultaneous presence of an acid and a base that would become instantaneously neutralised in the homogeneous phase, precluding the tandem process. Control studies with homogeneous acids and bases clearly show that it is not possible to perform this tandem reaction with soluble acid and bases.

In contrast, MOFs as well as other solid supports provide a rigid network to attach acid and basic sites at sufficient distances to avoid their annihilation by neutralisation. The importance of the simultaneous presence of (weak) acid and basic sites in solids to activate nucleophiles on the basic sites and the substrate on the acid sites is well known in heterogeneous catalysis and has been reported for instance by Corma and co-workers to explain the high catalytic activity of aminated aluminophosphates (AlPOs), even though the strength of the acid and basic sites is relatively weak.^365^ The simultaneous presence of acid and basic sites is also possible in MOFs with the advantage that these porous materials offer different alternatives, including a rich variation in the nature of the acid and basic sites, their location at the linkers or on nodal positions, and the generation of active sites by post-synthetic modification, and all of them on a highly crystalline, well-characterisable material. Several of these possibilities have been realised already.

**Fig. 24:**
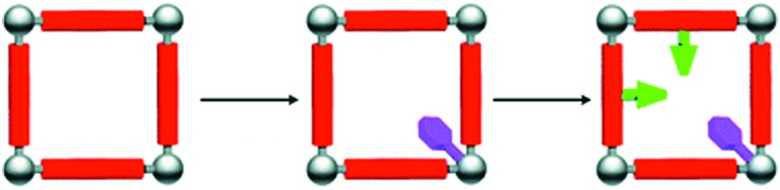
Cartoon illustrating the relative position of the sulphonic acid groups (green arrows) and basic amino groups (purple hexagons) in the lattice of MIL-101. Reproduced from ref. 366 with permission from the Royal Society of Chemistry, copyright 2012.

For instance, Shi and co-workers used MIL-101(Cr) and anchored basic sites (ethylendiamine) on the metal nodes and acid sites (sulphonic groups) on the organic linkers.^366^ In this way, the acid and basic sites are organic units and both of them were introduced sequentially by post-synthetic modification of the parent MIL-101(Cr) (see Fig. 24). Not surprisingly, the MIL-101(Cr)-SO_3_-NH_2_ was reusable – although only three consecutive runs were performed – and the inverse relationship between the size of the substrates and the yield of corresponding β-nitrostyrene suggests that the reaction should occur within the internal pores of the material.^366^ Importantly, control experiments using a mixture of *p*-toluenesulphonic acid and ethylenediamine, which are structurally closely related to the acid and basic sites in the MOF, show no conversion at all. Moreover, if any of these two organic compounds is added to MIL-101(Cr)-SO_3_-NH_2_, the tandem process is also impeded due to the neutralisation of the opposite site within the MOF pores.

**Fig. 25:**
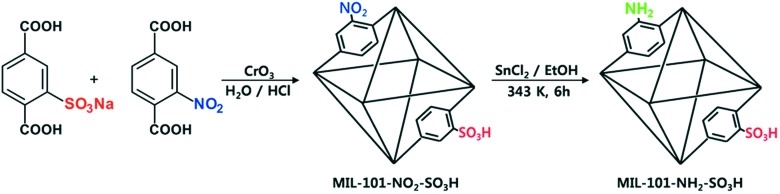
Preparation of mixed-ligand MIL-101(Cr) having isolated acid and base centres able to promote the deacetalisation/Henry condensation tandem process. Reproduced with permission from ref. 367 from the Royal Society of Chemistry, copyright 2014.

The same deacetalisation/Henry reaction tandem process has also been reported using a mixed-ligand MIL-101(Cr) as catalyst. This catalyst was synthesised using a mixture of two ligands, sulphonic terephthalate and nitro terephthalate, followed by SnCl_2_ reduction of the nitro to amino groups (Fig. 25).^367^ It was claimed that this synthesis was more convenient and led to a more efficient material than the one discussed above for MIL-101(Cr)-SO_3_-NH_2_, reaching somewhat higher nitrostyrene yields in shorter times and at lower temperatures.^367^

**Scheme 2:**
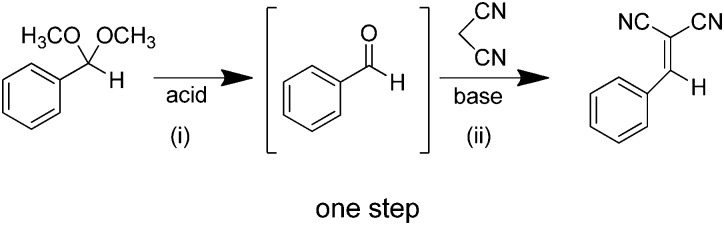
Tandem reaction showing (i) the deacetalisation of benzaldehyde dimethyl acetal forming benzaldehyde and (ii) the subsequent Knoevenagel condensation with malonitrile.

Another similarly preferred tandem test to evaluate the catalytic activity of solids having acid/base sites is the deacetalisation of benzaldehyde dimethyl acetal to form benzaldehyde, followed by the Knoevenagel condensation with malonitrile (Scheme 2). Also in this process, there are numerous soluble acids and bases that can promote each individual step separately and none of the two requires sites of strong acidity or basicity. In this context, Zhou and co-workers reported the synthesis of a copper paddlewheel-based MOF with 5,5′-(pyridine-3,5-dicarbonyl)bis(azanediyl)diisophthalate ligands (PCN-124).^368^ The exchangeable coordination positions at the Cu^2+^ sites and the pyridine and carboxyamide units are responsible for the acidity and basicity of the material with a self-interpenetrated 3D structure. The material did not lose activity in four consecutive uses and XRD characterisation of a four-times used sample shows the structural stability.

The same deacetalisation/Knoevenagel condensation tandem process has also been reported in MIL-101(Al)-NH_2_,^369^ whose activity was much higher than other solids with acid or base nature such as other MOFs, zeolite Y, and MgO, while a mixture of HCl and trimethylamine was inactive. In the case of MIL-101(Al)-NH_2_ the free –COOH groups of the linker present at structural defects or at the outer surface are believed to play the role of acid sites, while the basic sites are the amino groups of the linker. The superior activity of MIL-101(Al)-NH_2_ with respect to other MOFs (*e.g.*, MIL-53 or MIL-101(Cr), either with or without –NH_2_ groups) has been attributed to a combination of a high porosity (800 m^2^ g^–1^ with wide pore openings), with the simultaneous presence of –NH_2_ groups in the organic ligands and structural defects in the form of –COOH groups. MIL-101(Al)-NH_2_ was recycled three times, but showed neither a deterioration of its high catalytic activity nor a change in its selectivity.^369^

#### F.1.2. Acid-oxidation bifunctional catalysts

**Scheme 3:**
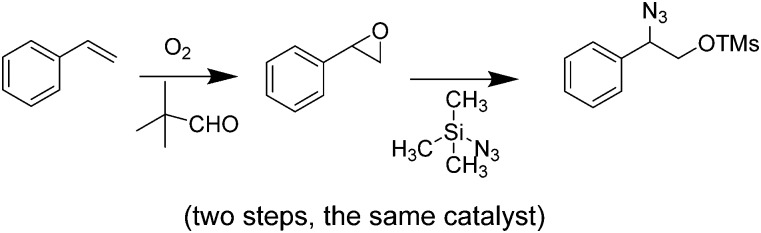
Conversion of styrene to the trimethylsilyl ether of phenyl azidohydrin using a tandem process.

Other typical tandem processes combine one acid- or base-catalysed step with an oxidation reaction. Thus, an iron-porphyrin MOF with Lewis acid Hf_6_ nodes has been reported to be active for the regioselective conversion of styrene to the trimethylsilyl ether of phenyl azidohydrin (Scheme 3).^370^ After MOF synthesis, chemical analysis established that the Fe/Hf atomic ratio was lower than the 2 : 6 according to the expected formula. To increase the iron content, the as-synthesised material was submitted to anhydrous FeCl_3_ treatment in DMF, but then the Fe/Hf ratio was consistently 4 : 6 and the characterisation data were compatible with two Fe^3+^ ions associated to the Hf_6_ nodes in addition to the iron-porphyrin. Interestingly, styrene epoxidation by molecular O_2_ using *tert*-butyraldehyde in the presence of azido trimethylsilane (TMSN_3_) affords the product of the tandem reaction with the opposite regioselectivity of the azido and trimethylsilyl (TMSO) groups than when styrene oxide is submitted to epoxide opening by TMSN_3_ with the same catalyst. This change in the regioselectivity depending on the nature of the starting materials, either styrene or styrene oxide, was intriguing, but confirmed with model compounds and known to originate in the first step of the tandem process. The change in the regioselectivity of the azido hydrin depending on whether the starting material is the styrene or styrene oxide remains unexplained and, certainly, requires further study.

**Scheme 4:**
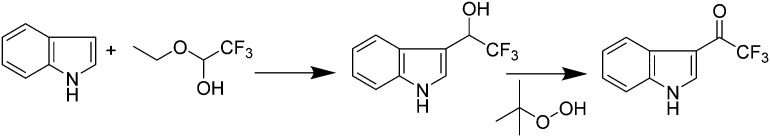
Tandem deacetalisation/Friedel–Crafts alkylation/oxidation.

In another tandem reaction having an oxidation step, a mixed metal MIL-100(Sc,Fe) was prepared and used as catalyst for the tandem deacetalisation/Friedel–Crafts alkylation/oxidation (Scheme 4).^371^ MIL-100(Sc) was found in previous work to be an excellent solid acid catalyst compared to other MIL-100(M) congeners, even if its activity does not correlate directly with the acid strength of the sites. The original feature of MIL-100(Sc,Fe) is that the two nodal metals have a different activity, scandium being the Lewis acid site promoting deacetalisation and alkylation, while iron, in its III oxidation state, is responsible for the oxidation of the benzylic alcohol to the heteroaromatic ketone when using TBHP as oxidant. It was noticed that the tandem process starting from indol and trifluoroacetaldehyde ethyl acetal affords the heteroaryl ketone in higher yield than when the presumed heteroaryl alcohol intermediate is used as starting material, a fact that was attributed to be higher efficiency of the oxidation when the intermediate is located already near the active sites as compared to the situation in which this compound has to diffuse to the active sites.^371^ This interesting observation shows that tandem processes may overcome the diffusion limitations normally posed by the intermediate, since they are already located near the active site in a tandem reaction, hence resulting in an improved catalyst performance.

**Fig. 26:**
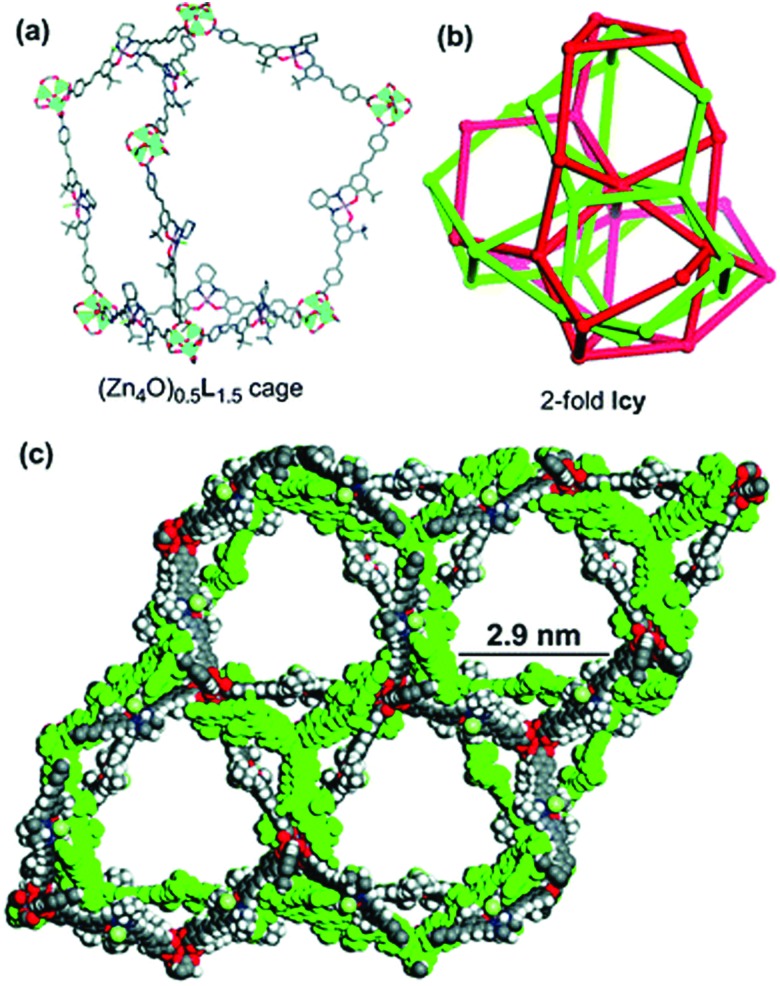
(a) Stick/polyhedra model of a chiral MOF showing the (Zn_4_O)_0.5_(Mn–salen)_1.5_ cage built from distorted octahedral secondary building units and dicarboxylate bridging Mn–salen complexes; (b) schematic showing the twofold interpenetrating networks of the **lcy** topology; (c) space-filling model of the structure of the double interpenetrated chiral MOF viewed perpendicular to the (001) plane. Reproduced from ref. 378 with permission from the Royal Society of Chemistry, copyright 2011.

**Scheme 5:**
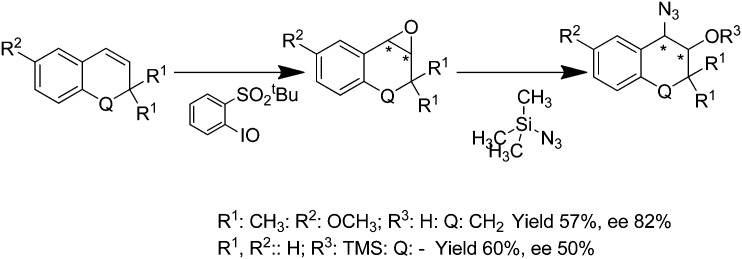
Alkene epoxidation-epoxide ring opening tandem process.

Perhaps one of the best examples of the versatility in the design and synthesis that MOFs offer in catalysis is their use as enantioselective catalysts. While most of the attempts to obtain homochiral zeolites or other porous solids that could be used as enantioselective catalysts have failed, there are ample precedents using MOFs in asymmetric catalysis.^372^,^373^ The simplest approach is to use an enantiometrically pure chiral linker as building block in the preparation of chiral MOFs.^374^ These homochiral MOFs have also been applied as catalysts of tandem reactions. The use of chiral manganese–salen complexes to promote the enantioselective epoxidation of simple alkenes by Jacobsen was a major achievement in the area since it expanded the initial enantioselective Sharpless epoxidation of allylic alcohols.^375^ Chiral metal–salen complexes are general catalysts for a series of asymmetric reactions including the asymmetric resolution of epoxides, the cyanosilylation of aldehydes, Diels–Alder cycloadditions, among many other reactions.^376^ Considering the advantages of heterogeneous catalysts in terms of recovery and recyclability of the catalysts, there was a considerable interest in anchoring or encapsulating these chiral complexes in organic polymers and inorganic solid supports.^377^ Not surprisingly, a MOF with a manganese–salen complex as building unit and Zn_4_O tetrahedra as inorganic nodes has been prepared (Fig. 26).^378^ The resulting chiral MOF is able to promote the enantioselective epoxidation of cyclic benzylic alkenes with a high to moderate enantiomeric excess (ee) using a substituted iodosylbenzene derivative as oxidant. In addition, the Lewis acidity of the Zn^2+^ ions is able to promote the regioselective epoxide ring opening using TMSN_3_ as nucleophile. Although no enantiomeric resolution of a racemic epoxide mixture was observed in this epoxide opening, the process was enantioselective and pure epoxide enantiomers afforded the corresponding azidohydrin with a high ee. Nevertheless, the chiral MOF based on the manganese–salen complex was able to perform the alkene epoxidation/epoxide ring opening tandem process (Scheme 5) with high to moderate enantioselectivity by the one-pot sequential addition of oxidising agent and, then, TMSN_3_.^378^

**Fig. 27:**
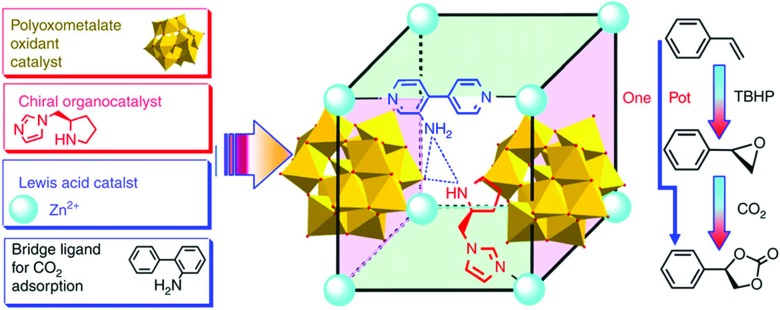
Components and their role, as well as a pictorial illustration, of the structure of the chiral zinc MOF that promotes the one-pot transformation of styrenes into the corresponding chiral cyclic carbonates with high ee values, reproduced from ref. 379 with permission from Nature Publishing Group, copyright 2015.

In another example, the enantioselective epoxidation of styrenes was followed by CO_2_ insertion to render the cyclic carbonate with a large to moderate enantiomeric excess.^379^ In this case, the MOF had several components that cooperated to the success of the tandem reaction. The active site for the oxidation reaction was an achiral Keggin polyoxometallate, ZnW_12_O_40_^6–^, and the asymmetric induction was performed by a chiral pyrazol pyrrolidine that was in close proximity to the oxidation centre mimicking in a certain way enzymes in which the protein backbone directs and establishes the interaction with the substrate in its approach to the prostetic active centre (Fig. 27). The MOF also contains an amino substituent in the 4,4′-bipyridine linker to increase the CO_2_ adsorption capacity of the material. Moreover, exchangeable positions around the Zn^2+^ nodal ions as well as tetrabutylammonium bromide act as Lewis acid and base to activate CO_2_ insertion. Interestingly, the opposite homochiral MOF, prepared in the same way but using the opposite enantiomer of the pyrazol pyrrolidine linker, gives rise to the opposite enantiomer of the cyclic phenyl carbonate in very similar ee, unveiling this linker as the unit responsible for the enantioselectivity of the process (Table 1).

**Table 1:** Yields and enantiomeric excess in the asymmetric epoxidation of the olefins, *a* in the coupling of CO_2_ to styrene oxide,^*b*^ and in the asymmetric auto-tandem catalysis of olefins to cyclic carbonates over ZnW-**PYI** catalysts.^*c*^ Reproduced from ref. 379 with permission from the Nature Publishing Group, copyright 2015

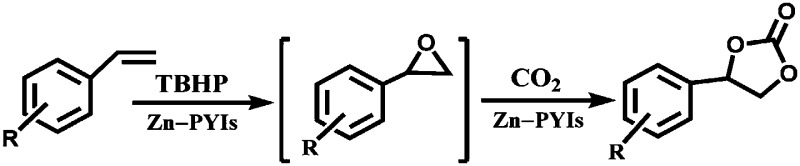				
Entry	Substrate	Product	Yield^*d*^ (%)	ee^*e*^ (%)
1	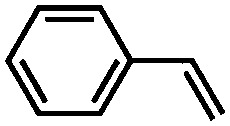	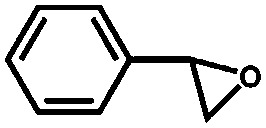	92(94)	79(–76)
2	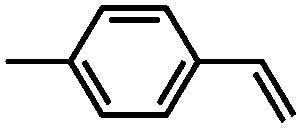	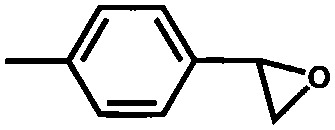	87(88)	75(–74)
3	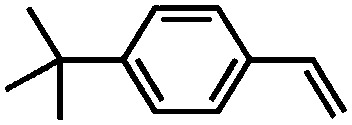	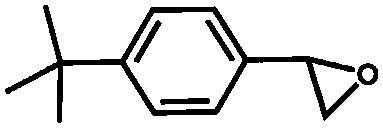	76(79)	75(–76)
4	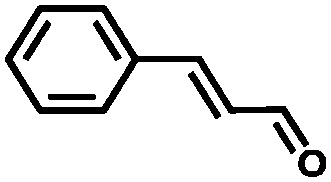	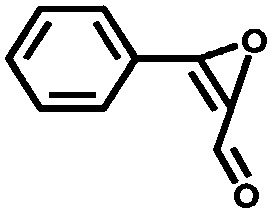	87(85)	93(–70)
5	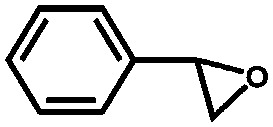	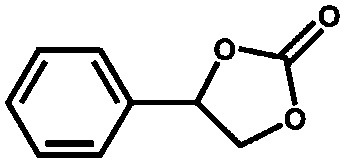	>99	Trace
6	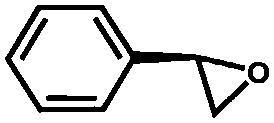	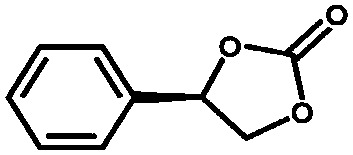	>99	90
7	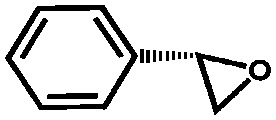	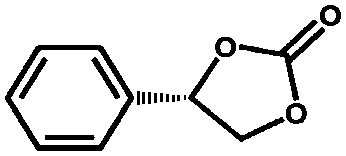	>99	–96
8	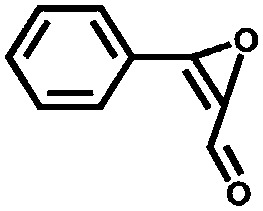	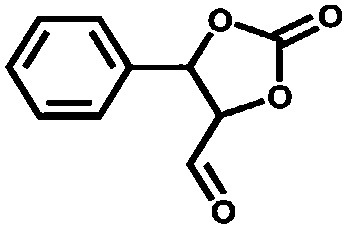	28	—
9	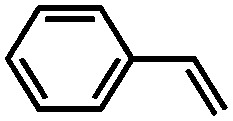	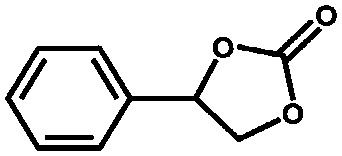	92(90)	80(–77)
10	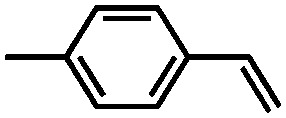	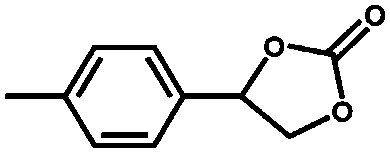	83(85)	70(–73)
11	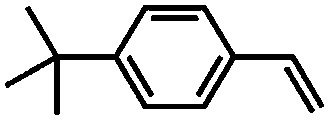	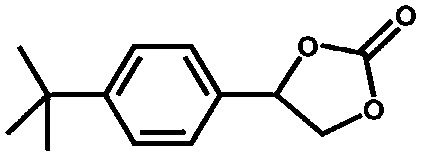	72(70)	55(–59)
12	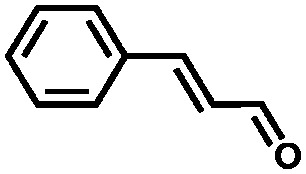	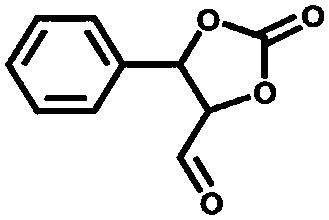	25	—

^*a*^Entries 1–4: conditions: olefin: 10 mmol, ZnW-**PYI**s: 0.01 mmol; TBHP (70% in decane): 20 mmol, 50 °C, 120 h.

^*b*^Entries 5–8: conditions: styrene oxide: 10 mol, catalyst: 0.01 mmol, TBABr 0.1 mmol, CO_2_, 0.5 MPa, 50 °C, 48 h.

^*c*^Entries 9–12: conditions: olefin: 10 mmol, catalyst: 0.01 mmol, TBHP 20 mmol, CO_2_, 0.5 MPa, 50 °C, 96 h.

^*d*^The yield was determined by ^1^H NMR spectroscopy of crude products. Yields catalysed by ZnW-**PYI**2 were listed in the parentheses.

^*e*^The ee value was determined by chiral HPLC on a Chiralcel OD-H column. The ee values catalysed by ZnW-**PYI**2 are listed in the parentheses.

#### F.1.3. Acid-reduction bifunctional catalysts

The reaction of salicylaldehyde with a pre-formed MOF containing free amino groups in its organic linkers (*e.g.*, aminoterephthalate-based MOFs such as IRMOF-3 or MIL-101-NH_2_) leads to the corresponding salicylidene-imine Schiff base under mild conditions. This simple post-synthetic modification strategy has been used successfully to introduce efficient chelating groups in MOFs. These chelating groups allow the coordination of additional metallic species that introduce desired new catalytic functions through a further metalation step.^380^,^381^ We recently used this approach to design bifunctional acid–metal catalysts by introducing palladium and platinum monoatomic salicylidene–imine complexes in a chromium aminoterephthalate MOF, MIL-101(Cr)-NH_2_, bearing coordinatively unsaturated Cr^3+^ sites with Lewis acid properties.^361^ The resulting bifunctional MOFs were used as catalysts in various tandem reactions consisting of the metal-mediated reduction of nitroarenes followed by the reductive amination of carbonyl compounds by the *in situ* formed aminoarene. These tandem processes catalysed by the bifunctional MOFs opened the door to the one-pot synthesis of important nitrogen-containing products, including secondary arylamines and nitrogen heterocyclic compounds (quinolines, pyrroles and pyrrolidines). The interplay between the Cr^3+^ Lewis acid sites of the MOF and the hydrogenation properties of the salicylidene metal complexes was found to be very convenient, in particular for tandem processes involving the reductive amination of ketones, Paal–Knorr synthesis of pyrroles, or Michael addition reactions, all requiring the assistance of acid sites with a moderate strength. In contrast, commercial catalysts based on palladium or platinum nanoparticles supported on carbon or alumina did not perform as well as the MIL-101 materials due to the lack of suitable acid sites to catalyse the above key reactions.^361^

**Fig. 28:**
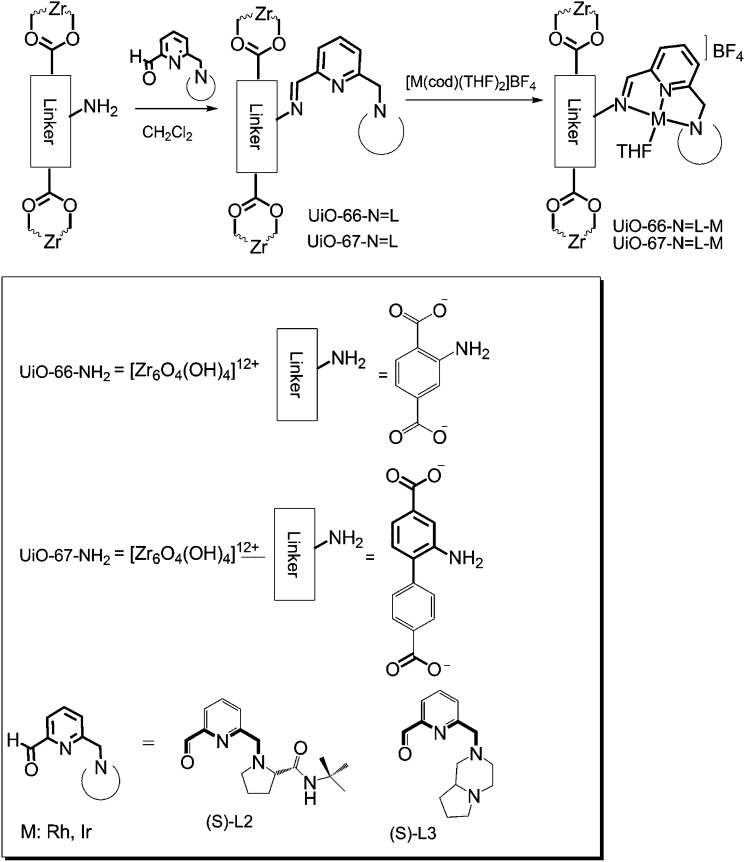
Post-synthetic modification of zirconium-MOFs with NNN pincer ligands. M = Rh, Ir. Reproduced from ref. 382 with permission from Wiley, copyright 2013.

A similar post-synthetic modification strategy was used by Rasero-Almansa *et al.* to modify the amino groups of the zirconium-containing MOFs UiO-66-NH_2_ and UiO-67-NH_2_, to produce various chiral NNN pincer aminopyridineimine ligands with chelating properties, followed by the introduction of iridium or rhodium (see Fig. 28).^382^ In this way, the authors developed a new family of multifunctional zirconium-based MOF catalysts containing (Rh, Ir) metallic centres, Zr^4+^ sites with Lewis acid properties, and base groups incorporated as ligands.

**Fig. 29:**
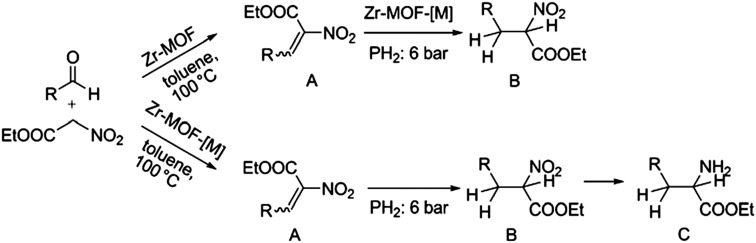
Tandem olefination-hydrogenation of aldehydes catalysed by multifunctional zirconium-MOF-[M] materials. Reproduced from ref. 382 with permission from Wiley, copyright 2013.

The post-synthetically modified multifunctional catalysts were tested in a number of reactions to evaluate the availability of the different incorporated functionalities, and were finally applied to a cascade process of olefination–hydrogenation of aldehydes. First, the basic groups of the ligands catalysed the Knoevenagel condensation of the aldehyde with nitroacetate to form product A (see Fig. 29), followed by the hydrogenation of the C=C bond of the intermediate product by rhodium (or iridium) sites to product B, and also catalysed the reduction of nitro to amino groups to product C at longer reaction times. However, it is worth mentioning that, in spite of using chiral NNN pincer ligands, the tandem process was found to be not enantioselective, even in the presence of a chiral diphosphine.^382^

### F.2. One-pot multicomponent coupling reactions

**Fig. 30:**
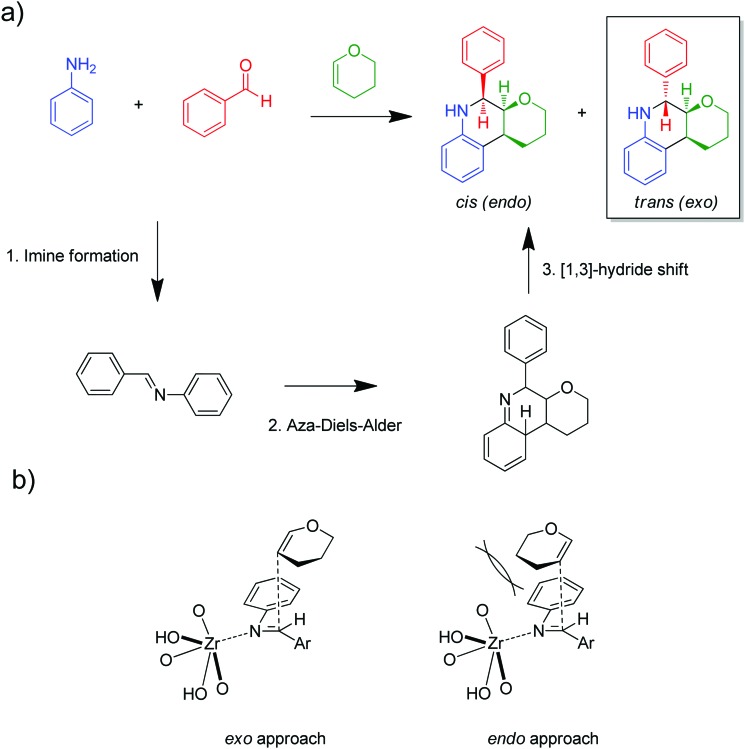
(a) Multicomponent Povarov coupling reaction of benzaldehyde, aniline, and dihydropyrane (DHP) leading to pyranoquinolines; (b) the zirconium-MOF acts as a “macroligand” favoring the *exo* addition of DHP to the coordinated imine, which results in the diastereoselective formation of the *trans* isomer.

Very recently, we have shown that the acidity of UiO-66 and the amino-modified derivative, UiO-66-NH_2_, renders these materials highly active and diastereoselective for the multicomponent coupling reaction between aldehydes, imines, and dihydropyrane (DHP).^383^ This so-called Povarov reaction leading to pyranoquinolines actually consists of a series of domino reactions starting from an inverse electron-demand aza-Diels–Alder cycloaddition of an aryl imine (formed *in situ* from the aldehyde and the amine) and an electron-rich dienophile (DHP), followed by a [1,3] hydride shift reaction (see Fig. 30).^384^ When UiO-66-type materials were used as catalysts, this cascade process was found to proceed smoothly at room temperature, yielding pyranoquinolines almost quantitatively after 20 h of reaction. Interestingly, the reaction proceeded with an excellent diastereoselectivity (diastereomeric excesses of 90–95%) to the corresponding *trans* isomer, stemming from the *exo* approach of DHP to the aryl imine during the aza-Diels–Alder cycloaddition. This high diastereoselectivity was attributed to the large steric effects of the MOF, preventing the *endo* addition and thus favouring the formation of the *trans* isomer. In this sense, the MOF was considered as a “macroligand” of the zirconium ions, which can drive the reaction pathway towards the formation of the less hindered product.

**Scheme 6:**
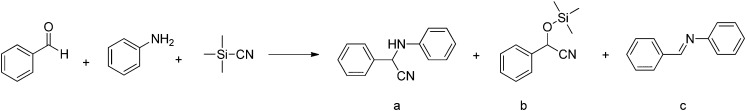
One-pot Strecker multicomponent reaction between benzaldehyde, aniline, and trimethylsilyl cyanide (TMSCN).

Very recently, Monge and co-workers reported on the synthesis and catalytic activity of mixed-metal MOFs with the general formula [In_*x*_Ga_1–*x*_(O_2_C_2_H_4_)_0.5_(HFIPBB)] (H_2_HFIPBB = 4,4′-(hexafluoroisopropylidene) bis(benzoic acid)).^385^ The series of monometallic Al, In, Ga, and bimetallic (In,Ga) compounds were all found to be active for the one-pot Strecker multicomponent reaction between benzaldehyde, aniline, and trimethylsilyl cyanide (TMSCN) to form the corresponding aminonitrile. Interestingly, the obtained products depended on the composition of the starting MOF (see Scheme 6): while AlPF-1 afforded the expected aminonitrile product (a), when GaPF-1 was used, the product coming from the aldehyde cyanosilylation (b) was obtained instead. Meanwhile, the imine product (c) was obtained over InPF-11, while the aminonitrile product formed quantitatively when the bimetallic (In,Ga) compound was used as catalyst. The rationalisation of the obtained results allowed the authors to suggest plausible reaction mechanisms, depending on the chemical composition of the used catalyst. Thus, activation of benzaldehyde at the Lewis acid sites lead to either cyanosilylation (GaPF-1) or imine formation, which in turn was followed by addition of TMSCN to the corresponding aminonitrile only in the case of AlPF-1 and bimetallic (In,Ga). This is a very nice example that illustrates the great potential of solid solution MOFs to address the various stages involved in the reaction mechanism, thus allowing to tune the final product.

## G. Towards enzyme mimics

The ultimate goal of implementing single catalytic sites into synthetic solids is to achieve the degree of sophistication displayed by enzymes. Reactivity in enzymes is usually defined by the chemical properties of the active site, typically composed of transition metal-based organometallic or inorganic compounds. However, selectivity and reaction rates are highly controlled through the interaction of the active site with the surrounding substructure of the enzyme and, in many cases, depend on cofactors. The typical hurdle to overcome is the implementation of all the subtle components that facilitate enzyme reactivity while, at the same time, controlling the design-reactivity in the synthetic target. In an aim to simplify the problem, different approaches have centred on chemically mimicking the structure of the active site whereas other approaches have gathered inspiration on inter- and intramolecular forces to construct similar reactive sites, hence focusing on the enzyme structure. Enzyme immobilisation is another strategy where the enzyme reactivity can be retained at a wider range of reaction conditions (*e.g.*, pH, solvent, concentration) by using an adequate support. However, in this contribution we will not touch upon enzyme immobilisation. In the literature, extensive reviews and perspectives can be found that account for enzyme-mimicking with MOFs.^386^–^389^ In the present contribution we highlight systems that: (1) mimic the chemical structure of enzyme active sites (2) mimic the metal-mediated reactivity by using different metal sites, and, lastly, (3) mimic the enzyme by exploiting the hydrogen bond sites.

### G.1. Hydrogenases

**Fig. 31:**
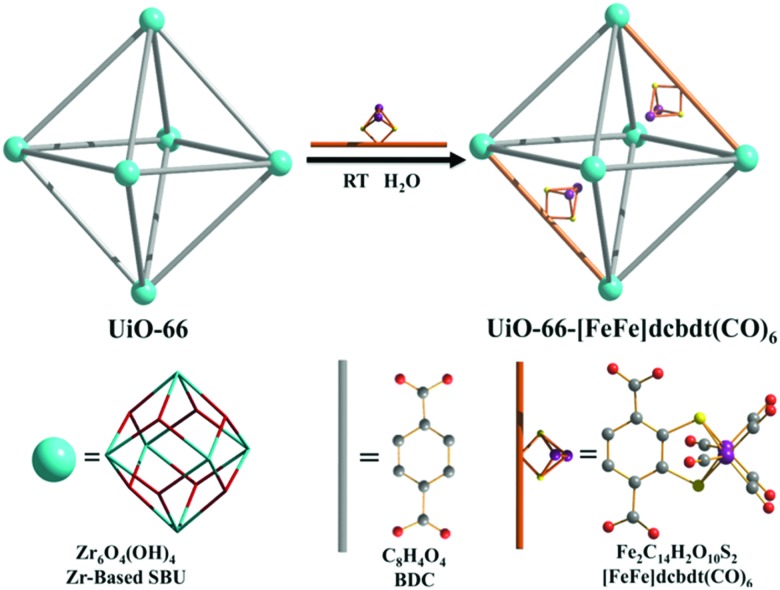
Schematic representation of the implementation of a hydrogenase mimicking active site by post-synthetic modification of a UiO-66-based MOF. Reproduced from ref. 392 with permission from the American Chemical Society, copyright 2013.

Hydrogen production from water is considered one of the most promising solutions to store solar energy in the form of chemical bonds. In nature, many micro-organisms benefit from the equilibrium between H_2_ and protons and use an enzyme-mediated process either to obtain energy from H_2_ or to use H_2_ as an electron sink. Hydrogenases are a large family of enzymes that are mostly classified based on their active centre. The [FeNi] hydrogenases are more involved in hydrogen oxidation processes, whereas the [FeFe] hydrogenases are more related to hydrogen production. The active structures of the [NiFe], [FeFe], and [Fe] hydrogenases are the core for inspiration of biomimetic models, and although there are hundreds of structural models, the mimetic models are still in their infancy. Among the synthetic models that have attracted the most attention are the diiron hexacarbonyl models. In these systems, fine-tuning the first and second coordination sphere and not only using carbonyl groups lead to hydrogen evolving activities closer to the performance of enzymes, although with greater overpotentials (0.5 V).^390^ It has also been demonstrated from mutagenic studies that even the third coordination sphere is crucial for the performance of the enzyme.^391^ Given these facts, the field of metallo-enzymes mimics can highly benefit from MOF-based models. Although there is still a long road to explore, there are already some examples that have mimicked the dimeric metal active site of the hydrogenases. In an early example, Cohen and co-workers reported the incorporation of a Fe–Fe dimeric hexacarbonyl complex by attaching it to UiO-66 as a modified BDC linker (Fig. 31).^392^ The resulting catalyst was shown to be active in the photocatalytic hydrogen evolution reaction (HER) by using [Ru(bpy)_3_]^2+^ as photosensitiser and ascorbate as electron donor.^392^ Whereas the synthesis of the MOF proved impossible by directly incorporating the Fe–Fe modified dicarboxylate during the solvothermal synthesis, the authors reported the ease of the synthesis by post-synthetic modification (PSM). They achieved to exchange about 16% of the nonfunctionalised linkers to their Fe–Fe functionalised counterparts and demonstrated their incorporation in the MOF structure (see Fig. 31). The Fe–Fe dimer metal site was also determined intact after the PSM with EXAFS. Moreover, the heterogeneous electron transfer (ET) between UiO-66-[FeFe](dcbdt)(CO)_6_ and [Ru(bpy)_3_]^2+^, estimated at ∼300 mV, proved the light-driven reduction of the iron sites. This example was the first proof of concept to demonstrate the possibilities that MOFs can offer to stabilise and enhance reactivity for enzyme-mimicking models. There are other examples where MOFs are used as a photosensitiser. Typical strategies use the incorporation of organic light harvesting units such as amino terephthalates, porphyrins or even the incorporation of coordination sites to anchor the photosensitiser/photocatalyst. The activity of porphyrins to act as an antenna was demonstrated in hydrogen production and this concept was used by Rosseinsky and co-workers.^393^,^394^ The concept of using photosensitised MOFs with porphyrins as linkers was further coupled with the supramolecular attachment of the Fe–Fe dimer *via* a pyridine-functionalised dithiolate ligand. Thus, the group of Feng *et al.* reported a system in which the zirconium–porphyrin MOF ZrPF, the photosensitiser, was coupled to the reduction [Fe_2_S_2_] catalyst [(i-SCH_2_)_2_NC(O)C_5_H_4_N]-[Fe_2_(CO)_6_] for light-driven HER. Once more, the resulting complex proved more efficient than its homogeneous counterpart.^395^ Remarkably, in the homogeneous version of the [Fe_2_S_2_] complex, the CO ligands are lost after 40 minutes of reaction and therefore the catalyst deactivates, whereas [Fe_2_S_2_]@ZrPF still shows the characteristic CO vibration in FTIR and displays stable activity after similar reaction times.

More recently, Nasalevich *et al.*^396^ developed a ship in a bottle approach for the efficient encapsulation of a derivative of a well-defined cobaloxime proton reduction catalyst within a photoresponsive MOF (NH_2_-MIL-125(Ti)). The resulting hybrid system is a fully recyclable and, to date, one of the most efficient noble metal-free catalyst system for light-driven hydrogen evolution from water under visible light illumination based on a MOF. In this case, the MOF is used to harvest light and as electron buffer for the actuation of the Co (electro)catalysts.

### G.2. Degradation of chemical warfare agents

Organophosphates are important molecules found in biochemical signalling processes as well as in enzymatic cofactors for a wide range of living organisms. Besides their natural occurrence, the interest in developing synthetic methodologies to hydrolyse the phosphate ester bond is due to the accumulation of synthetic organophosphates used for pesticides and insecticides in soils and aquatic bodies.^397^ Nerve agents are another class of organophosphates and are among the most dangerous chemical warfare agents (CWA). This subclass of CWA is mostly volatile and able to disrupt the nervous system by blocking the acetylcholinesterase (AChE) and causing accumulation of acetylcholine, the neurotransmitter to relay nerve impulses. Since the chemical signalling is localised in the neuromuscular junctions, its accumulation can lead to disruptions in the nervous system, generating perpetual signalling in the nerve, blocking muscular movement, and eventually leading to asphyxiation.^398^,^399^

Phosphotriesterase (PTE) is an enzyme able to catalyse the hydrolysis of a wide range of phosphotriesters.^398^ The active centre, containing bimetallic zinc clusters bridged by hydroxide anions and carbamylate functional groups, has served as an inspiration to mimic its reactivity. The cluster is surrounded by four histidine molecules, two for each zinc atom. The two zinc atoms differ from each other in their coordination: one is fully coordinated, by an aspartic acid residue, whereas the second is exposed to the solvent when the substrate is absent.^400^ The binuclear centre, bridged by a carboxylate, is found in similar enzymes able to hydrolyse the phosphate ester bond.^401^ Moreover, when replacing the zinc centres by other metals, the activity can be preserved.^402^ Therefore, in light of the wide tunability of metal clusters in MOFs, Peterson *et al.* investigated the activity of the undercoordinated “paddlewheel” copper site in HKUST-1 to hydrolyse venomous agent X (VX), soman (GD), and distilled mustard (HD).^403^ The copper-mediated hydrolysis was observed in all cases but with disappointingly low reaction rates, affording at most a half-life of 13 h for HD and over a day for VX and GD. The same group had already demonstrated the favourable reaction rates obtained by zirconium–metal hydroxides in the hydrolysis of HD, VX, and GD.^404^ This precedent was used as argument by the group of Hupp and Farha to explore the phosphate hydrolysis activity of zirconium-based MOFs. Although the activity of the UiO-66(Zr) framework is still lagging behind compared to the PTE in the hydrolysis of methyl paraxon (dimethyl 4-nitrophenyl phosphate, DMNP) and its aryl analogue (4.2 × 10^–6^ and 1.6 × 10^–6^ M s^–1^, respectively),^405^ it served as a platform to develop and apply similar structures to the UiO-66 with the same Zr_6_ node but longer linkers and with fewer linkers interconnecting the nodes, such as the NU-1000 and MOF-808.^406^–^408^ Although the catalytic efficiency of UiO-66 was already a good milestone, the authors found that, due to the relative large substrate molecules and therefore diffusion limitations of the substrate molecules into the pores of the framework, the observed catalytic activity resulted only from those zirconium clusters located at the surface of the crystallites. A large drop in surface area was found after reaction, but no deactivation was observed, nor any loss of crystallinity. By digesting the framework, some phosphorous-containing residues could be tracked, most likely blocking the pores. However, since the catalysis mostly took place on the surface, the change in porosity did not have an important role in the activity of these materials. The authors then focused on improving the activity by preserving the metal node but using instead a larger tetradentate linker.^407^ The resulting NU-1000 structure has an exceptionally wide channel size (31 Å) and this enables diffusion of the organophosphate ester into the pores, thus achieving greater activity per gram of catalysts. The activity when using a *N*-ethylmorpholine buffered solution of DMNP and 6 mol% of catalyst (based on zirconium equivalents) is three times higher in NU-1000 than in UiO-66. Moreover, although the Zr_6_ unit [Zr_6_(μ_3_-O)_4_(μ_3_-OH)_4_(H_2_O)_4_(OH)_4_] is preserved in NU-1000, there are important structural differences compared to the [Zr_6_(μ_3_-O)_4_(μ_3_-OH)_4_] in UiO-66 reflecting the changes in chemical reactivity. The NU-1000 nodes are coordinatively unsaturated because they are coordinated to only eight linkers instead of twelve in the defect-free UiO-66 structure.

In a further study, another zirconium-based MOF with the same hexamer was used, but with a different topology. In MOF-808, the metal node is only sixfold connected as a result of capping half of the metal node sites with formate ions during synthesis. The resulting MOF-808 can be activated by heating the MOF in a hot solvent and exchanging the six formate ions by six water molecules and six hydroxide ions. With this number of unsaturated active sites at the metal node, an impressive activity for the hydrolysis of dimethyl 4-nitrophenyl phosphate (DMNP) was achieved, increasing the TOF from 0.004, 0.14, and 0.09 s^–1^ for UiO-66, UiO-66-NH_2_, and dehydrated NU-1000, respectively, to TOF values of 1.4 s^–1^ for MOF-808.^408^

### G.3. Hydrogen bond donor catalysis

In an aim to exploit the highly ordered crystal structures that MOFs offer, the incorporation of catalysts that would normally suffer from deactivation as a result of clustering or self-aggregation is a promising avenue. In this regard, hydrogen bond donor catalysis has emerged as a biomimetic alternative to Lewis acid activation. Hydrogen bond donor catalysis relies on the use of hydrogen bonding interactions to accelerate and control organic reactions. Such mechanisms are commonly found in enzyme catalysis, but also a whole range of organocatalytic reactions make use of this approach.^409^–^411^ A common problem with these organocatalysts is that they tend to self-aggregate because, next to the hydrogen bond donor motifs, they contain hydrogen bond acceptor motifs as well.^412^–^414^ In practice, this means that activity is lost because active sites are occupied, but also that the activity of these catalysts depends very strongly on temperature, solvent polarity, and the presence of salts. In many cases, these reactions suffer from a loss of selectivity coinciding with the loss of activity, because there is a significant amount of nonselective background reactivity. In enzymes, the problem of self-aggregation does not exist because the active sites are isolated and a single binding motif in each active site can be achieved. The same principle can be achieved by the isolation of the catalytic unit in a solid support. The chymotrypsin enzyme, which hydrolyses peptides, can be seen as a mechanistically related example. In this case, cooperative hydrogen bonding plays a major role by reorienting the substrate into a suitable conformation, leading to lower activation barriers. The chymotrypsin achieves a high activity by attacking deactivated carboxylic groups with a strong nucleophile. Ureas,^415^ thioureas,^416^ and squarimides^417^ are motifs that proved very useful as hydrogen bond donor catalysts. They have been applied in a variety of reactions, like conjugate additions, Diels–Alder reactions, Henry reactions, and Friedel–Crafts reactions.

Farha, Hupp, and Scheidt managed to incorporate a urea unit in a zinc-based MOF, the NU-601, by using bis-(3-isophthalic)-urea as a linker.^418^ Interestingly, the NU-601 framework is not catalytically active immediately after synthesis, because the active sites are blocked by the DMF used in the synthesis. The usual procedure to remove the solvent, by heating, proved ineffective because the framework decomposes at these higher temperatures. However, solvent exchange with nitromethane allowed framework activation and the formation of an active catalyst for the Friedel–Crafts reaction of pyrroles with nitroalkenes, displaying a higher activity than diphenylurea. Having said this, the authors did not compare the activity of this NU-601 catalyst with that of other more electron-poor ureas. Moreover, they also showed that for large substrates the reaction is slowed down or even blocked, emphasising the importance of the pore size.

In the same line, Che and co-workers reported the functionalisation of MIL-101(Cr) with different ureas and the application to the Friedel–Crafts alkylation between *trans-b*-nitrostyrene and *N*-methyl pyrrole.^419^ Both electron-withdrawing and electron-donating groups in the phenyl ring were well-tolerated and afforded good yields. In addition, the group of Hupp incorporated ureas in the zirconium-MOF UiO-67 to catalyse the Henry reaction.^420^ In this case, the ureas are attached to BPDC linkers. Remarkably, when using the fully functionalised UiO-67-urea, the MOF was not catalytically active. It was concluded that the pores were too crowded. As a solution, they used a mixed-linker approach by combining the urea-modified BPDC with nonfunctionalised BPDC. This yielded a MOF with increased porosity, and indeed catalytic activity was obtained: the MOF was more active than a comparable homogeneous urea. In a third contribution, Hupp, Farha, and Mirkin incorporated squarimides in UiO-66, also following a mixed-linker approach, very similar to the previous example with UiO-67-urea.^421^ These MOFs are active in the Friedel–Crafts reaction between unsubstituted indol and *b*-nitrostyrene.

In another approach to decorate MOFs with urea-like functionalities by post-synthetic functionalisation, the group of Wang immobilised a thiourea functionality into IRMOF-3.^422^ The pending amino group yields a urea-modified MOF upon reaction with an isocyanate, while reaction with an isothiocyanate yields a thiourea-modified MOF. These frameworks were successfully used in Friedel–Crafts, acetalisation, and Morita–Baylis–Hillman reactions. The above examples show that hydrogen bond donor catalysts can be incorporated into MOFs to prevent their self-aggregation. It is shown in a few examples that these isolated catalysts are indeed more active than their homogeneous counterparts. Recycling is shown in one example as well.

## H. Outlook and future perspectives

Heterogeneous single-site catalysis has the potential to combine the best of homogeneous and heterogeneous catalysts into a single synthetic solid. Because of the almost unlimited design possibilities, both MOFs and POFs are ideal materials for the implementation of these sites in such a way that the distance between catalytic functions can be altered at will. This results in very important advantages such as a higher stability compared to homogeneous counterparts and the addition of extra functionalities such as shape selectivity or specific interactions.

The last few years have witnessed an impressive advancement in the development of new synthetic methods related to the implementation and maximisation of single-site catalytic functions in MOFs. Especially interesting are the controlled generation of defects and the application of post-synthetic cation exchange to generate sites with unprecedented reactivities. However, we believe that defect generation may become a victim of its own success, since the stability of the framework is clearly compromised upon creation of coordination vacancies. Moreover, most catalytic performances presented so far – except from some outstanding examples – are not overwhelming. In Section B.2 we have highlighted two terrific examples from Dincă's group on the post-synthetic cation exchange of nickel and iron in two different MOFs.^101^,^102^ In these examples, the MOF has to be seen as a macroligand that affects – or tunes – the electronic configuration of the active metal. Such an idea opens the door to a new design pathway where solid ligands become readily available and may be the start of a new research field in catalysis with unprecedented opportunities. We believe that over the next few years many more examples in this direction will appear.

From this review, it is obvious that catalysis with POFs and COFs is still in its infancy. What an infancy however! In a previous perspective article, some of us advocated for a fair comparison between new catalytic systems (based on MOFs in that case) and state-of-the-art catalysts as a way to convince the reader of the benefits of new materials.^32^ This practice has certainly been applied in the field of POF catalysis, demonstrating that, in most cases, activities similar to their homogeneous counterparts can be achieved upon immobilisation of single-site catalytic moieties in POFs. Moreover, the outstanding stability of most of these solids allows for their application under a wider range of conditions. Methane activation *via* Periana chemistry^167^–^169^ and formic acid decomposition^173^ – with the highest productivities reported to date for a solid catalysts – are two clear examples of the potential of these materials.

In order to achieve a faster progress in these topics, the level of understanding has to increase. To this task, modelling is going to play an instrumental role. Reaction profiles for catalytic reactions within MOFs have so far been obtained using static calculations as outlined in Section D.1.2. These static approaches hence only account for a limited number of points on the potential energy surface. For the schematic reaction profile shown in Fig. 13(a), this would boil down to calculating the electronic energies for the adsorbed reactants, transition state, adsorbed products, and desorbed state. However, at real operating conditions, chemical transformations taking place at the nanometre scale may be very complex in nature due to the interplay of several factors such as the number of particles present in the pores of the material, framework flexibility, competitive pathways, and entropic effects, among others. The textbook concept of a single transition state is far too simplistic in such cases, and is often insufficient to capture the complexity of the transformation. In these cases, one should construct the complex free energy surface (FES) along the important reaction coordinates of the system. Advanced sampling methods such as molecular dynamics techniques have been developed and allow exploring larger regions of the FES. Within the field of zeolites, these methods are making their entrance to describe complex reactive transformations.^260^,^423^,^424^ For MOFs, such an assessment would be a next promising step to bridge the gap between experiment and theoretical predictions.

An illustration of an active site where such advanced sampling methods might be useful concerns an undercoordinated brick of the UiO-66 material that may be hydrated or not (see Fig. 13). Active site 1 is a visual representation of a dehydrated brick, whereas active site 2 is the hydrated brick for which the metal is capped by water molecules. For selected case studies, an effect of adding water to the system was observed experimentally. To mimic such effect theoretically, various approaches may be followed. Statically, one may obtain first insights into the mechanism on the two active sites and construct a free energy profile on both the hydrated and dehydrated bricks. However, when the reaction is assisted by guest molecules, one needs to account for the dynamical state of the water or other guest species in the pores of the material. For reactions taking place in zeolites, such dynamical assessment of water on the reaction mechanism has been investigated, while a similar investigation on MOFs and POFs is still missing. Apart from obtaining detailed mechanistic insight into particular reactions, computational methods can also be used to rapidly screen or design *in silico* promising framework materials for specific catalytic reactions. This approach was recently followed by Vogiatzis *et al.* to identify framework materials that may act as hosts for high-valence iron(iv)–oxo species, which are known to activate strong C–H bonds.^425^ However, stabilising the high-spin state in molecular species used in homogeneous catalysis is difficult and thus alternative routes have been explored to stabilise the complex in a heterogeneous host. To find potentially interesting host materials for the high-valence iron complexes, computational screening procedures form a viable approach. It is impossible to synthesise and experimentally test the huge amount of MOFs that have been reported so far. Various high-throughput screening studies are available for applications such as gas storage and separation, but within catalysis such screening is less common.^426^ A screening study is only meaningful when it is much faster or cheaper than experimental synthesis and therefore it needs to rely on descriptors that may easily be obtained for a large set of materials. Vogiatzis *et al.* set up a multi-step approach based on both geometric and electronic criteria to screen materials with coordinatively unsaturated iron(ii) centres that can activate N_2_O and support a high-spin iron(iv)–oxo intermediate.^425^ The screening procedure, which started from a database containing more than 5000 known MOFs, resulted in three viable materials for this catalytic application. The computational screening study was performed on perfect materials. It is well known that defects are inherently present in MOFs and thus future computational screening studies should also account for defective materials. This is certainly a highly ambitious task. Screening studies are thus making their entrance to characterize heterogeneous single-site catalysts and are complementary to detailed insight into the reaction cycle obtained with computationally very expensive methods.

In Section F of our review, we have highlighted a number of studies on the application of MOFs in multifunctional catalysis. Indeed, this might be an excellent application niche for MOFs and POFs. By enclosing different single-site catalytic functions within a single framework, process intensification on the one hand and easier activation of substrates on the other hand may render superior catalytic systems. It goes without saying that we are not yet at the point where every catalytic function can be implemented and therefore the implementation of several functions is even trickier. However, looking at the pace at which synthetic methods advance, it would not be overly controversial to state that in a few years this dream may become a reality.

In our research proposals we always tend to claim that enzymes are used as source of inspiration for catalyst design, whether we actually try to mimic such exquisite systems or not being of a lower importance. From a scientific point of view, mimicking nature is as attractive as challenging and we do not need to convince the reader about how important it would be to develop synthetic tools able to replicate enzyme selectivities. In contrast, when it comes to stability and scope of application, as catalyst designers, we want to go much further than enzymes do. Freely quoting the recent Nobel laureate Ben Feringa: “*we took inspiration from birds to build airplanes even when at this moment we are not able to synthesize a single cell of a bird*”. We need therefore to learn much more from the way enzymes work to be able to translate this knowledge to synthetic solids that not necessarily need to be replicates of such enzymes. For instance, the facts that still make many enzymes unique are (i) the encapsulation of reactants in the vicinity of the reaction centre, *e.g.* promoting a given rebound mechanism and (ii) the non-covalent interactions with the protein matrix that help product removal and suppress side-reactions. While most research so far has focused on the exact replication of active sites (see *e.g.* the examples of hydrogenases mimics), far less attention has been paid to the design of the surroundings of the active site. In this sense, rational control over the framework flexibility of the material may also add great advances in terms of catalyst design and should certainly be considered in future developments where materials are capable of adapting their pore space by conformational changes of their building units.

Last but not least, although not touched upon in this review, before a large-scale application of these materials becomes feasible, synthesis scale-up – something that may be extremely tricky with materials such as CTFs – and catalyst shaping will be a must. When considering the relatively poor mechanical properties of these materials, traditional methods involving high pressure shaping will not be an option. Therefore either coatings or other methods towards self-supported agglomerates, like spray drying,^427^ electrochemical coatings,^428^ or the use of alternative binders will need further research.^176^

In summary, research into single-site catalysis on MOFs and POFs is already contributing to a much better understanding of heterogeneous catalysis and has the potential to be not only a game changer in catalyst design for a number of processes usually dominated by homogeneous catalysis, but also to open the door to new reactivity concepts with a plethora of potential applications.
